# Synthetic Route to Glycosyl β-1C-(phosphino)-phosphonates as Unprecedented Stable Glycosyl Diphosphate Analogs and Their Preliminary Biological Evaluation

**DOI:** 10.3390/molecules25214969

**Published:** 2020-10-27

**Authors:** Michaël Bosco, Su-Jin Paik, Patricia Busca, Stuart E. H. Moore, Christine Gravier-Pelletier

**Affiliations:** 1 Université de Paris, Faculté des Sciences, Campus Saint-Germain-des-Prés, UMR CNRS 8601, LCBPT, 45 rue des Saints Pères, F-75006 Paris, France; michael.bosco@u-paris.fr (M.B.); patricia.busca@u-paris.fr (P.B.); 2 Université de Paris, Faculté de Médecine Xavier Bichat, INSERM U1149, CRI, 16 rue Henri Huchard, F-75018 Paris, France; su-jin.paik@inserm.fr (S.-J.P.); stuart.moore@inserm.fr (S.E.H.M.)

**Keywords:** *C*-glycoside, diphosphate analogs, phosphinophosphonate, alkaline diphosphatase, glycochemistry

## Abstract

The synthesis of glycosyl-β-1*C*-(phosphino)-phosphonates is a challenge since it has not yet been described. In this paper, we report an innovative synthetic method for their preparation from Glc-, Man-, and Glc*N*Ac- lactone derivatives. The proposed original strategy involves the addition of the corresponding δ-hexonolactones onto the dianion of (methylphosphino) phosphonate as a key step, followed by dehydration and stereoselective addition of dihydrogen on the resulting double bond. Final deprotection provides the new glycosyl diphosphate analogs in 35%, 36%, and 10% yield over 6 steps from the corresponding δ-hexonolactones. The synthetized compounds were evaluated as inhibitors of phosphatase and diphosphatase activities and found to have complex concentration-dependent activatory and inhibitory properties on alkaline phosphatase. The synthetized tools should be useful to study other enzymes such as transferases.

## 1. Introduction

**Figure 1:**
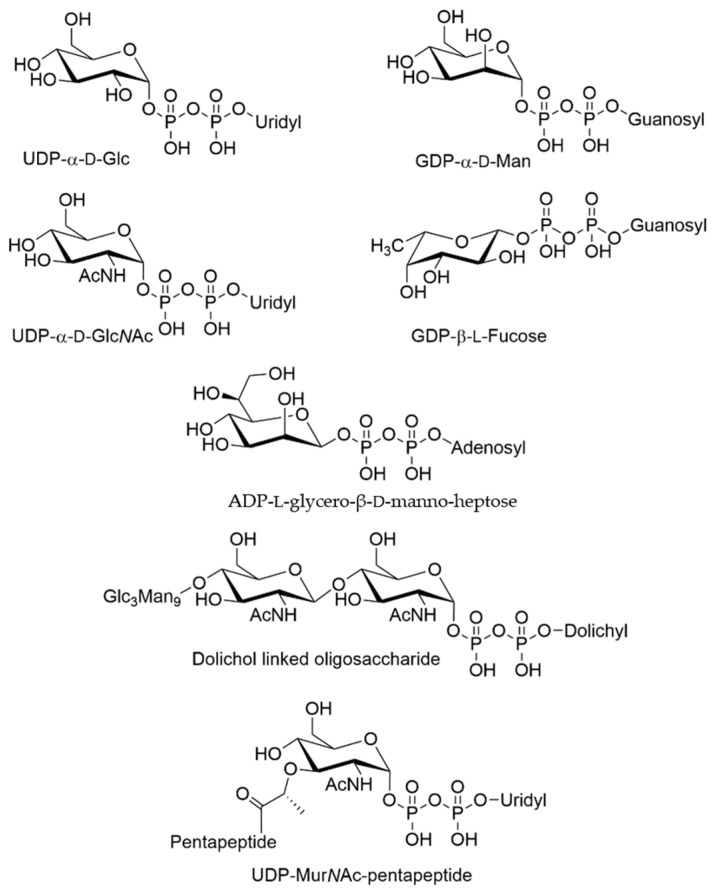
Biologically relevant glycosyl diphosphates**.**

Glycosyl diphosphates are key constituents of biologically-active compounds. In glycosylation reactions catalyzed by glycosyl transferases, monosaccharides are activated as α- or β-glycosyl esters of nucleotides, which are major glycosylating agents in the biosynthesis of oligosaccharides. Examples of nucleotide sugars such as UDP-α-d-Glc, GDP-α-d-Man, GDP-β-l-Fuc, or ADP-l-*glycero*-β-d-*manno*-heptose are shown in Figure 1. They are also involved as glycosyl donors in glycoconjugate biosynthesis [1,2] such as *N*-linked glycoproteins [3,4,5] resulting from the transfer of a polysaccharide from a dolichol linked oligosaccharide (DLO, Figure 1) onto polypeptides. Noteworthy, UDP-Glc*N*Ac (Figure 1) which is involved in this biosynthetic pathway is also a key intermediate in the biosynthesis of bacterial peptidoglycan precursors [6] such as UDP-Mur*N*Ac-pentapeptide (Figure 1), the nucleotidic substrate of the enzymatic reaction catalyzed by the bacterial transferase MraY [7,8], highlighting the analogies in lipopolysaccharide and glycoprotein biosynthesis [9].

**Figure 2:**
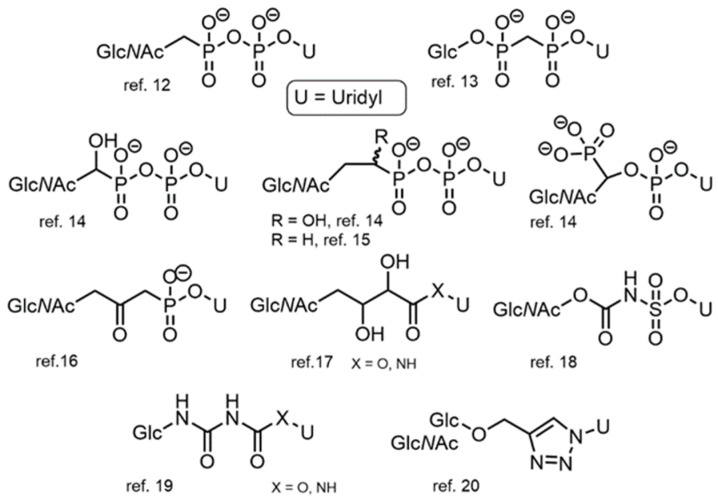
Examples of synthetic analogs of UDP-Glc or UDP-Glc*N*Ac**.**

Due to their central role in carbohydrate metabolism, much attention has been paid to the development of synthetic strategies towards non-hydrolysable diphosphate analogs of nucleotide sugars, notably towards potential inhibitors of enzymes involved in glycosyl phosphate metabolism. UDP-Glc or UDP-Glc*N*Ac were the most studied and research efforts led to the synthesis of several analogs with very variable structures. Representative synthetic analogs are outlined in Figure 2. On the one hand, phosphonate derivatives in which the oxygen atom in either the anomeric position of the sugar [10,11,12] or between the phosphorus atoms [13] has been replaced by a methylene group have been reported. On the other hand, the chain length between the sugar and the diphosphate has been increased [14,15]. In other diphosphate analogs, one of the phosphorus atoms has been replaced by a carbonyl group [16]. Various linkers lacking any phosphorus atoms have also been envisaged as diphosphate mimics such as an α,β-dihydroxy ketone [17] or an oxycarbonylaminosulfonyl [18]. A β-d-glycosyl residue has also been linked to uridine in allophanates and biuret derivatives [19]. Finally, the isosteric replacement of a diphosphate by a triazole linker [20] was also described. Noteworthy, diphosphate mimics involving other sugars or bases notably include a carbonyl linked to a *O*- or *N*-sulfamoyl uridine [21], tartaric [22], saccharidic [23,24], and squaryldiamide [25] linkers.

**Figure 3:**
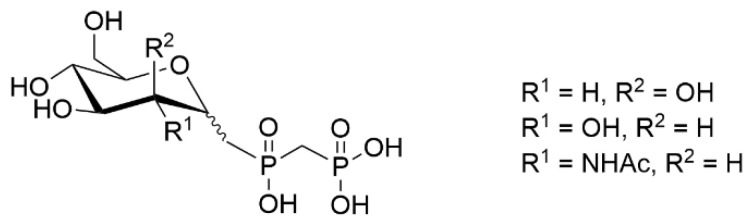
Structure of the targeted compounds**.**

In this context, our goal was to develop a straightforward access to original analogs of glycosyl diphosphate with an unprecedented β-1-*C*-(phosphino)-phosphonate structure. On the one hand, the replacement of an oxygen atom at the anomeric position of a saccharidic unit by a methylene group is a well-documented strategy [26,27] to prevent the cleavage of the aglycon part of the molecule during hydrolysis by glycosidases. On the other hand, the proposed diphosphate analog should have the property to chelate a divalent cation as diphosphates often do, while limiting the cleavage between the phosphorus atoms due to the poor leaving group character of a phosphonate compared to that of a phosphate. To extend the scope of the proposed method, the preparation of Man-, Glc-, and Glc*N*Ac-diphosphate analogs has been envisaged (Figure 3).

**Figure 4:**
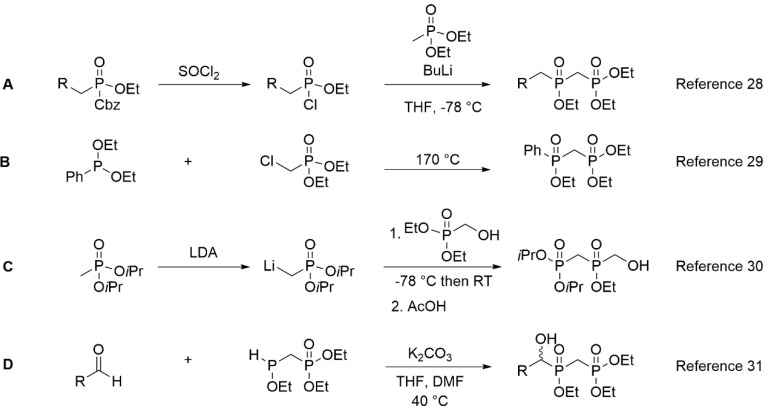
Phosphinophosphinate synthesis strategies already described on non-glycosidic molecules. (**A**) Nucleophilic substitution of the chlorine atom of a chlorophosphinate by a lithio phosphonate. (**B**) Michaelis-Arbuzov reaction. (**C**) Phospha-Claisen condensation. (**D**) Condensation of a *H*-(phosphonyl)phosphonate reagent onto an aldehyde.

The synthesis of a phosphino-phosphonate skeleton has never been described for monosaccharide derivatives. Nevertheless, this phosphorus scaffold was synthesized according to different pathways (Figure 4) by nucleophilic substitution of the chlorine atom of a farnesylchlorophosphinate by a lithio phosphonate [28] (Figure 4A) or by the Michaelis-Arbuzov reaction of a phenylphosphite on a (chloromethylene)phosphonate (Figure 4B). However, such a reaction involves rather drastic conditions [29] that might not be the most appropriate for sensitive molecules such as glycosides and would involve the sequential introduction of phosphorus derivatives on a glycosyl compound. A phospha-Claisen condensation involving the preparation of a lithio phosphonate that reacts with an (hydroxymethylene)phosphonate [30] (Figure 4C) presents the advantage of affording a phosphinophosphonate bearing an hydroxymethylene that can be subsequently functionalized. However, no functionalization of such a reagent with a glycosyl derivative has been reported, yet and preliminary assays were not successful in our hands. In a more straightforward manner, a *H*-(phosphonyl)phosphonate reagent [31] has been condensed onto an aldehyde in smooth conditions (Figure 4D) but its preparation requires several steps and this reagent displays limited stability. In summary, the synthesis of the targeted compounds represents a major challenge.

**Figure 5:**
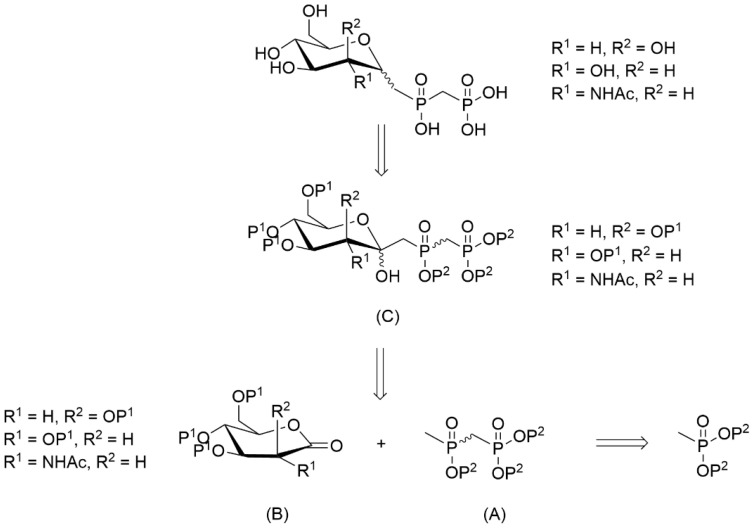
Retrosynthetic analysis.

We focused on developing a convergent strategy towards these compounds that relies on the retrosynthetic analysis outlined in Figure 5, allowing us to introduce simultaneously both phosphorus atoms in a single step according to a straightforward route. Access to the targeted phosphino-phosphonate would involve the addition of the anion of methyl-(phosphino)-phosphonate (A) onto the lactones (B) derived from commercially available α-methyl mannoside or glucoside and α,β-Glc*N*Ac, respectively (Figure 5) leading to the corresponding lactols (C). Dehydration followed by sequential hydrogenation of the resulting double bond and deprotections would afford the targeted compounds.

## 2. Results and Discussion

### 2.1. Chemistry

**Scheme 1:**
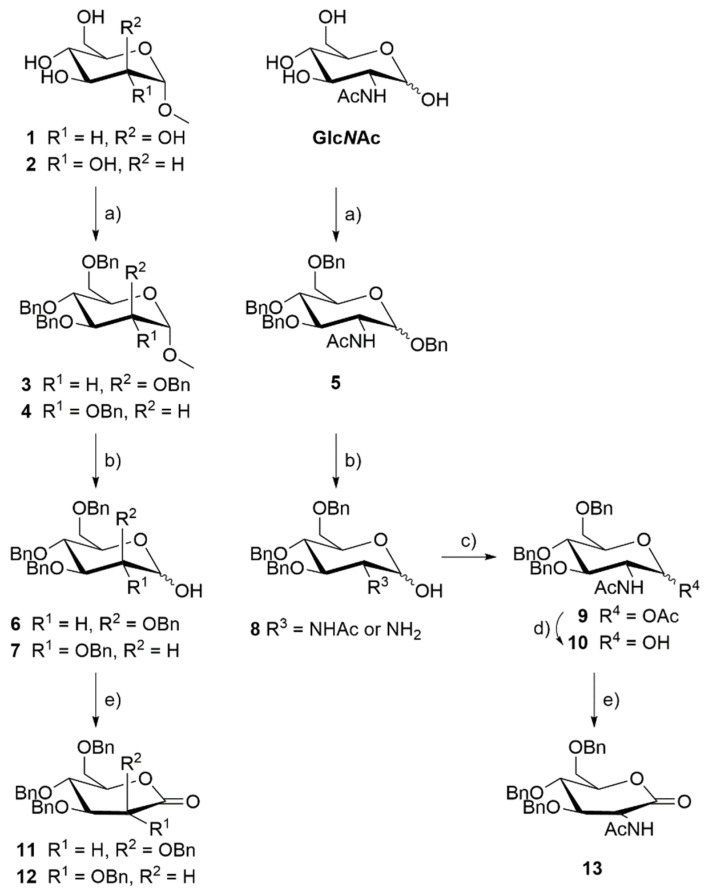
Reagents and Conditions: (**a**) BnBr, NaH, DMF, 0 °C to RT, 3 days, 84% for **3**, 92% for **4**, or 16 h, 67% for **5**. (**b**) HCl, CH_3_COOH, H_2_O, 100 °C, 3 h, 58% for **6** and 58% for **7**. (**c**) Ac_2_O, Et_3_N, DMAP, CH_2_Cl_2_, 0 °C to RT, 66% from 5 over two steps. (**d**) MeONa, MeOH, 1 h, RT, 77%. (**e**) I_2_, K_2_CO_3_, CH_2_Cl_2_, RT, 16 h, 73% for **11**, 67% for **12**, 36% for **13**.

We first turned our attention to the preparation of the δ-hexonolactones **11**–**13** which is outlined in Scheme 1. We aimed to develop a similar and simple pathway to access these three δ-hexonolactones. The synthesis of the δ-hexalactone **13** using the proposed pathway has not been reported previously. The perbenzylation of commercially available α-methyl mannoside **1** or glucoside **2** was carried out in DMF in the presence of sodium hydride and benzyl bromide in excess to afford the protected derivatives **3** and **4** in excellent yield. Similarly, perbenzylation of a commercial α,β-Glc*N*Ac mixture led to the benzyl acetal of perbenzylated Glc*N*Ac **5**. Deprotection at the anomeric position of compounds **3** and **4** was efficiently achieved by heating at 100 °C in the presence of hydrochloric acid in a 80% aqueous acetic acid solution giving the hemiketal **6** and **7**. It has to be noted that the deprotection at the anomeric position of compound **3** performed in the same acidic conditions was accompanied by partial deprotection of the acetamide into the corresponding amine, giving the hemiketal **8**. Nevertheless, a two-step sequence involving the acetylation of compound **8** by acetic anhydride in the presence of triethylamine and dimethylaminopyridine leading to the intermediate acetamide **9** (65% yield over two steps from **5**), followed by methanolysis at the anomeric position (77% yield) furnished the expected hemiketal **10**. Oxidation at the anomeric position of lactols **6**, **7** and **10** was efficiently carried out by molecular iodine [32] in the presence of potassium carbonate in dichloromethane to give the lactones **11** [33], **12** [34] and **13** [35], respectively.

**Scheme 2:**
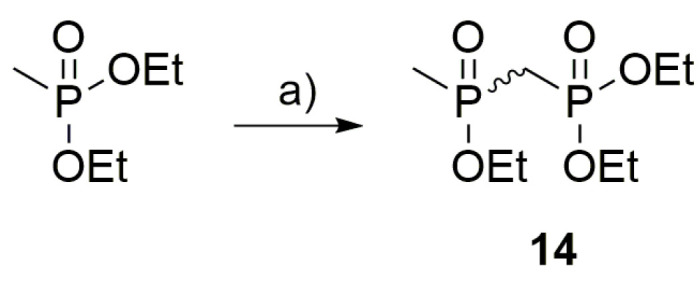
Reagents and Conditions: (**a**) *n*BuLi, THF, −78 °C to RT, 3 h, 91%.

We next turned to the preparation of diethyl ethoxy(methyl)phosphinylmethyl phosphonate **14** (Scheme 2) that was obtained in 91% yield by autocondensation of commercially available diethyl methyl phosphonate in the presence of butyl lithium in THF [36].

**Scheme 3:**
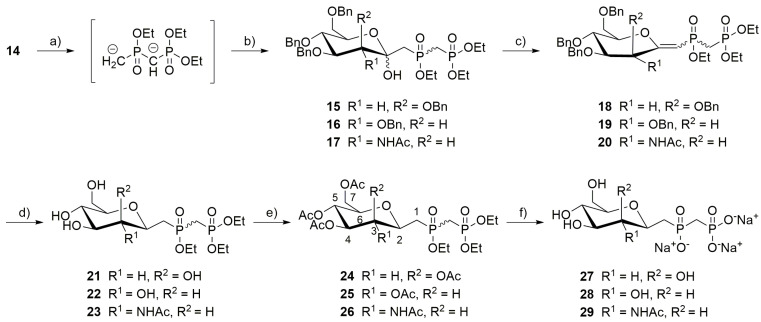
Reagents and conditions: (**a**) *n*BuLi, THF, −78 °C, 1 h. (**b**) **11**, **12** or **13**, THF, −78 °C, 2 h, 58% for **15**, 58% for **16**, 25% for **17**. (**c**) (CF_3_CO)_2_O, pyridine, CH_2_Cl_2_, 0 °C, 3 h, 80% for **18**, 70% for **19**, 63% for **20**. (**d**) H_2_, Pd/C 10%, EtOH, RT, 16 h. (**e**) Ac_2_O, pyridine, 80% for **24**, 96% for **25**, 73% for **26**. (**f**) i. TMSBr, CH_2_Cl_2_, 0 °C to RT, 16h; ii. NaOH, H_2_O, RT, 16 h, 95% for **27**, 92% for **28**, 85% for **29**.

The synthesis of the targeted glycophosphinophosphonates is outlined in Scheme 3. First, the dianion of the methylphosphinyl phosphinate **14** was generated by the addition of a strong base, in THF, prior to the addition of the lactones **11**–**13**. Different bases and temperature conditions were tested to efficiently perform this reaction. They notably include the sequential use of NaH at room temperature to remove the most acidic proton of the methylene between phosphorus atoms, followed by *n*BuLi addition at −78 °C [37] to generate the second anion from the methyl group or the single use of *n*BuLi at −78 °C. These latter conditions revealed to be the most efficient. The addition of the lactones **11**–**13** onto the resulting dianion, at −78 °C, afforded the corresponding 1-*C*-phosphinyl phosphonates lactols **15**–**17** in moderate yields [38]. Compounds **15** and **16** were present as complex mixtures of diastereoisomers resulting from two elements of chirality, α and β anomers and a stereogenic phosphorus atom. For compound **17**, in addition to the same diastereoisomers, rotamers, resulting from the *N*HAc moiety, are also observed. Then, the β-elimination of a water molecule was carried out by treatment with trifluoroactetic anhydride in the presence of pyridine in dichloromethane [39] to afford the substituted *exo-*glycals **18**–**20** in good yields and as mixtures of four possible diastereoisomers due to the presence of both the double bond and the stereogenic phosphorus atom. The stereoselective reduction of the double bond by hydrogenation [40,41,42,43] in the presence of 10% palladium on charcoal in EtOH was accompanied by benzyl ether hydrogenolysis to give the deprotected glycosyl phosphinophosphonate **21**–**23**. These latter compounds show a single R configuration for the stereogenic centre C2 with characteristic coupling constants (*J*_2,3_ = 9.0 Hz for **22** and 9.5 Hz for **26** resulting from **23**) and are present as mixtures of two diastereoisomers (stereogenic phosphorus atom). For compound **21**, the R configuration for the stereogenic centre C2 was confirmed by NOESY experiments after acetylation. Peracetylation of compounds **21**–**23** by acetic anhydride in pyridine was then performed to avoid side reactions during phosphinophosphonate deprotection and afforded the corresponding compounds **24**–**26** in high yield. The R configuration for the stereogenic centre C2 of compounds **24** and **25** could be unambiguously assigned thanks to the observed correlation between H_2_ and H_4_ and H_2_ and H_6_ as demonstrated by the superimposition of COSY and NOESY spectra (see Supplementary Materials S22 and S24). Finally, the targeted compounds **27**–**29** were obtained according to a two-step sequence involving the deprotection of the phosphinophosphonate moiety by trimethylsilyl bromide in dichloromethane followed by saponification of the acetate protecting groups to afford the corresponding targeted compounds as single stereoisomers.

### 2.2. Biological Studies

With these tools in hand, we next turned to their biological evaluation. Compounds **27**, **28**, and **29** were evaluated for their ability to inhibit di and monophosphatase activities. Dolichol linked oligosaccharide diphosphatase was used as an example of diphosphatase because we have been studying its activity for a long time [44]. Alkaline phosphatase was chosen as a representative example of monophosphatase.

**Figure 6:**
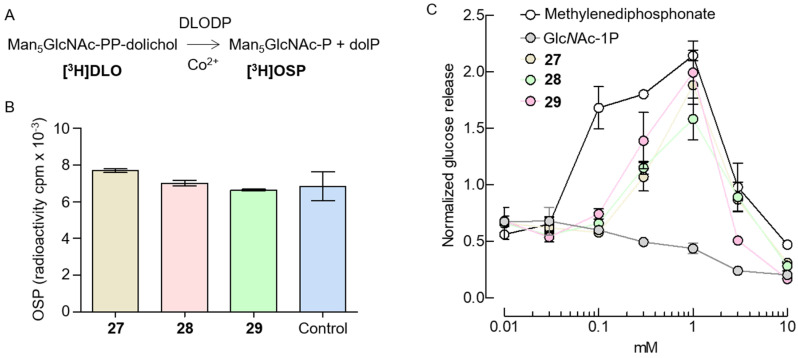
Evaluation of the capacity of compounds to inhibit DLO diphosphatase and alkaline phosphatase. (**A**) Dolichol linked oligosaccharides (DLO) are hydrolysed by oligosaccharyl diphosphodolichol diphosphatase (DLODP) to yield oligosaccharyl phosphates (OSP) and dolichyl phosphate (dolP). (**B**) DLODP activity was measured, as detailed in Methods, in the presence of 1 mM **27**, **28**, and **29** (*n* = 2, mean +/S.D.). (**C**) Alkaline phosphatase was assayed by quantitating the dephosphorylation of [^3^H]Glc-1P as described in Methods. Compounds **27**, **28**, and **29** (as well as sodium methylenediphosphonate and Glc*N*Ac-1-phosphate (Glc*N*Ac-1P) were included in incubation mixtures at the indicated concentrations. Alkaline phosphatase activity is expressed as a fraction of that observed in control incubations conducted in the absence of added compounds (*n* = 2, mean +/S.D.).

Oligosaccharyl diphospho dolichol diphosphatase (DLODP) cleaves between the two phosphate residues of DLO that are required for protein *N-*glycosylation in the lumen of the endoplasmic reticulum (ER) [44]. The physiological function of this activity is not known, but oligosaccharylphosphates (OSP), which are products of DLODP action on DLO, are seen in increased quantities in cells derived from patients with congenital disorders of glycosylation (CDG) where truncated DLO accumulate. The proteins and genes responsible for this activity have not been identified and at present there are no known selective DLODP inhibitors. Little is known about the biochemistry of the DLODP activity but in vitro experiments using rat liver microsomes indicate that cleavage of both radioactive DLO ([^3^H]Man_9-5_Glc*N*Ac_2_-PP-dolichol, [44]) and fluorescent bacterial lipid II (Glc*N*Ac-Mur*N*Ac(dansyl-pentapeptide)-PP-undecaprenol, [45] requires detergent and Co^2+^. Compounds **27**, **28**, and **29** were tested at a concentration of 1 mM in the standard DLODP assay using detergent-solubilized rat liver microsomes in the presence of 40 nM [^3^H]Man_5_Glc*N*Ac_2_-PP-dolichol substrate (Figure 6A). The reaction is followed by the liberation of OSP ([^3^H]Man_5_Glc*N*Ac_2_-P, Figure 6A). As shown in Figure 6B, at this concentration the 3 compounds had no significant effect on DLODP activity.

Next, we investigated the effects of compounds **27**, **28**, and **29** on the ability of alkaline phosphatase to dephosphorylate [^3^H]Glc-1P. Data presented in Figure 6C indicate complex concentration-dependent effects of these molecules on AP activity towards [^3^H]Glc-1P. In fact, all 3 compounds behaved similarly, and at concentrations below 0.05 mM weakly inhibited dephosphorylation of the substrate whereas between 0.1 and 1.0 mM they provoked 1.5-2-fold increases in the reaction. At higher concentrations, inhibition of the reaction occurred. In order to understand the origin of this complex behavior, we tested the effects of sodium methylene diphosphonate on AP-mediated dephosphorylation of [^3^H]Glc-1P. Although the potentiation phase of the dose response curve seems to occur at lower concentrations and reach a higher maximum, the data show that this compound has very similar effects to those of **27**, **28**, and **29** (Figure 6C). Finally, the effects of Glc*N*Ac-1-phosphate (Glc*N*Ac-1P) were tested in the assay and as shown in Figure 6C this compound, in contrast to **27**, **28**, and **29**, did not reveal an activatory capacity over the same concentration range.

## 3. Materials and Methods

### 3.1. Chemical Synthesis

MS and/or analytical data were obtained using chromatographically homogeneous samples. 

^1^H NMR (500 MHz), ^13^C NMR (126 MHz), and ^31^P (202 MHz) spectra were recorded a Bruker Avance or Avance II (Bruker BioSpin, Wissembourg, France) in the given solvents unless otherwise indicated. Chemical shifts (*δ*) are reported in ppm and coupling constants are given in Hz. Low resolution mass spectra (LRMS) were recorded with an ion trap mass analyzer (LCQ Advantage, ThermoFisher, Bremen, Germany) under electrospray ionization (ESI) in positive and negative ionization mode detection. High resolution mass sprectra (HRMS) were recorded with an Orbitrap mass analyzer (Exactive, ThermoFisher, Bremen, Germany) under electrospray ionization (ESI) in positive or negative ionization mode detection, atmospheric pressure chemical ionization. All reactions were carried out under an argon atmosphere, and were monitored by thin-layer chromatography with Merck 60F-254 precoated silica (0.2 mm, Merck, Darmstadt, Germany) on glass. Flash chromatography was performed with Merck Kieselgel 60 (200–500 µm, VWR, Leuven, Belgium); the solvent systems were given *v*/*v*.

#### 3.1.1. Diethyl {[ethoxy(methyl)phosphoryl]methyl}phosphonate **14**

A solution of diethyl methylphosphonate (7.6 g, 49.6 mmol) in anhydrous THF (15 mL) under argon atmosphere was cooled by a dry ice/acetone bath. To this solution was added dropwise a solution of *n*-butyllithium (1.6 M in hexanes, 32 mL, 51.2 mmol) during 30 min. Then the reaction mixture was allowed to warm to room temperature. After 2 h of stirring, the reaction was quenched by addition of an aqueous solution of hydrochloric acid (3N, 25 mL). The reaction mixture was extracted two times with dichloromethane (2 × 75 mL). The organic layers were combined, dried over anhydrous sodium sulfate, filtered, and concentrated under vacuo to obtain the crude product (5.74 g, 22.2 mmol, 89%) as a colorless oil. ^1^H NMR (500 MHz, CDCl_3_) *δ* 4.22–3.99 (m, 6H, 3 × OC*H_2_*CH_3_), 2.38 (dd, *J* = 20.7, 16.9 Hz, 2H, PC*H_2_*P), 1.66 (d, *J* = 15.0 Hz, 3H, C*H_3_*P), 1.32 (t, *J* = 7.1 Hz, 6H, 2 × OCH_2_C*H_3_*), 1.31 (t, *J* = 7.1 Hz, 3H, OCH_2_C*H_3_*); ^13^C NMR (126 MHz, CDCl_3_) *δ* 62.6 (d, *J* = 6.4 Hz, O*C*H_2_CH_3_), 62.5 (d, *J* = 6.5 Hz, O*C*H_2_CH_3_), 60.8 (d, *J* = 6.4 Hz, O*C*H_2_CH_3_), 29.0 (dd, *J* = 134.5, 81.2 Hz, P*C*H_2_P), 16.6 (d, *J* = 6.4 Hz, OCH_2_*C*H_3_), 16.4 (d, *J* = 6.3 Hz, OCH_2_*C*H_3_), 16.3 (d, *J* = 6.3 Hz, OCH_2_*C*H_3_), 15.8 (d, *J* = 100.7 Hz, *C*H_3_P); ^31^P NMR (202 MHz, CDCl_3_) *δ* 44.5 (d, *J* = 3.4 Hz, CH_3_*P*CH_2_P), 19.9 (d, *J* = 3.4 Hz, CH_3_PCH_2_*P*); ESI-MS: Calcd for C_8_H_20_NaO_5_P_2_: 281.1 [M + Na]^+^ Found: 281.1; ESI-HRMS: Calcd for C_8_H_21_O_5_P_2_: 259.0864 [M + H]^+^ Found: 259.0865.

#### 3.1.2. 3,4,5,7-Tetra-*O*-benzyl-1-deoxy-1-{[(diethoxyphosphoryl)methyl](ethoxy)phosphoryl}-α-d-*manno*-2-heptulopyranose **15**

A solution of diethyl {[ethoxy(methyl)phosphoryl]methyl}phosphonate **14** (1.725 g, 6.68 mmol) in anhydrous THF (9 mL) under argon atmosphere was cooled by a dry ice/acetone bath. To this solution was added dropwise a solution of *n*-butyllithium (1.6 M in hexanes, 7 mL, 11.2 mmol) during 20 min. After stirring during two hours at −78 °C, a solution of 2,3,4,5-tetra-*O*-benzyl-*mannono*-1,5-lactone **11** (1.2 g, 2.23 mmol) in anhydrous THF (6 mL) was added dropwise to the reaction mixture. After stirring during two hours at −78 °C, the reaction was quenched by addition of a solution of acetic acid in THF (33% *v*/*v* in THF, 6 mL). The reaction mixture was diluted with 30 mL of ethyl acetate and 45 mL of water. To this mixture was added 12 g of sodium chloride. The organic layer was collected and the aqueous layer was extracted with 45 mL of ethyl acetate. The organic layers were combined, dried over anhydrous sodium sulfate, filtered, and concentrated. Column chromatography (silica gel 125 mL, EtOAc then EtOAc/EtOH, 98/2, *v*/*v*) of the residue afforded the product **15** as a mixture of stereoisomers (1.04 g, 1.30 mmol, 58%) as a colorless oil. ^1^H NMR (500 MHz, CDCl_3_) *δ* 7.47–7.10 (m, 20H, H aromatic), 5.01–4.42 (m, 8H, 4 × OC*H_2_*Ph), 4.25–3.99 (m, 8H, H-4, 3 × POC*H_2_*CH_3_, H-6), 3.94, 3.91 (2t, *J* = 9.7 Hz, 1H, H-5), 3.77, 3.76 (2d, *J* = 2.7 Hz, 1H, H-3), 3.74–3.67 (m, 1H, H-7), 3.67, 3.65 (2dd, *J* = 11.7, 2.2 Hz, *J* = 10.8, 1.8 Hz, 1H, H-7′), 2.92, 2.66 (2td, *J* = 20.5, 15.8 Hz, *J* = 20.0, 15.8 Hz, 1H, P*CH_2_*P), 2.85, 2.74 (2dd, *J* = 15.1, 11.5 Hz, *J* = 15.3, 13.6 Hz, 1H, H-1), 2.52–2.30 (m, 1H, P*CH_2_*P), 2.23, 1.77 (2dd, *J* = 15.3, 11.5 Hz, *J* = 19.8, 15.2 Hz, 1H, H-1′), 1.33–1.17 (m, 9H, 3 × POCH_2_CH_3_); ^13^C NMR (126 MHz, CDCl_3_) *δ* 138.75, 138.72, 138.66, 138.64, 138.59, 138.50, 138.46 (4 × Cq aromatic), 128.56, 128.54, 128.42, 128.39, 128.3, 128.13, 128.08, 127.91, 127.88, 127.8, 127.71, 127.69, 127.60 (20 × CH aromatic), 98.2, 97.8 (2d, *J* = 8.8 Hz, *J* = 5.5 Hz, C-2), 81.5, 81.4 (2d, *J* = 2.5 Hz, *J* = 2.3 Hz, C-4), 78.9, 78.4 (2d, *J* = 9.7 Hz, *J* = 9.2 Hz, C-3), 75.2 (O*C*H_2_Ph), 75.0, 74.9, 74.8, 73.3, 73.2, 73.03, 72.97 (4 × O*C*H_2_Ph), 75.1 (C-5), 72.4, 71.9 (C-6), 69.8, 69.7 (C-7), 63.0, 62.8, 62.7, 62.6, 62.44, 61.39 (6d, *J* = 6.4 Hz, *J* = 6.3 Hz, *J* = 6.5 Hz, *J* = 6.6 Hz, *J* = 6.4 Hz, *J* = 6.7 Hz, 3 × O*C*H_2_CH_3_), 37.3, 35.2 (2d, *J* = 92 Hz, *J* = 94 Hz, C-1), 30.2, 29.1 (2dd, *J* = 135, 87 Hz, *J* = 134, 84 Hz, P*C*H_2_P), 16.58–16.29 (m, 3 × POCH_2_*C*H_3_); ^31^P NMR (202 MHz, CDCl_3_) *δ* 48.3, 46.7 (2d, *J* = 11.5 Hz, *J* = 3.4 Hz, CH_2_*P*CH_2_P), 20.0, 19.6 (2d, *J* = 11.5 Hz, *J* = 3.4 Hz, 1P, CH_2_PCH_2_*P*); ESI-MS: Calcd for C_42_H_54_NaO_11_P_2_: 819.8 [M + Na]^+^ Found: 819.2; ESI-HRMS: Calcd for C_42_H_54_NaO_11_P_2_: 819.3034 [M + Na]^+^ Found: 819.3035.

#### 3.1.3. 3,4,5,7-Tetra-*O*-benzyl-1-deoxy-1-{[(diethoxyphosphoryl)methyl](ethoxy)phosphoryl} -α-d-*gluco*-2-heptulopyranose **16**

A solution of diethyl {[ethoxy(methyl)phosphoryl]methyl}phosphonate **14** (2.87 g, 11.1 mmol) in anhydrous THF (15 mL) under argon atmosphere was cooled by a dry ice/acetone bath. To this solution was added dropwise a solution of *n*-butyllithium (1.6 M in hexanes, 11.6 mL, 18.6 mmol) during 20 min. After stirring during two hours at −78 °C, a solution of 2,3,4,5-tetra-*O*-benzyl-*glucono*-1,5-lactone **12** (2 g, 3.71 mmol) in anhydrous THF (10 mL) was added dropwise to the reaction mixture. After stirring during two hours at −78 °C, the reaction was quenched by addition of a solution of acetic acid in THF (28% *v*/*v* in THF, 7 mL). The reaction mixture was diluted with 50 mL of ethyl acetate and 75 mL of water. To this mixture was added 20 g of sodium chloride. The organic layer was collected and the aqueous layer was extracted with 75 mL of ethyl acetate. The organic layers were combined, dried over anhydrous sodium sulfate, filtered, and concentrated. Column chromatography (silica gel 200 mL, EtOAc) of the residue afforded the product **16** as a mixture of stereoisomers (1.736 g, 2.17 mmol, 58%) as a colorless oil. ^1^H NMR (500 MHz, CDCl_3_) *δ* 7.41–7.12 (m, 20H, H aromatic), 4.98, 4.96, 4.92, 4.91, 4.89, 4.88, 4.84, 4.83, 4.69, 4.65, 4.57, 4.55, 4.53, 4.50, 4.45, 4.44 (16d, *J* = 11.7 Hz, *J* = 11.4 Hz, *J* = 11.0 Hz, *J* = 10.9 Hz, *J* = 10.9 Hz, *J* = 11.0 Hz, *J* = 11.0 Hz, *J* = 11.0 Hz, *J* = 11.4 Hz, *J* = 11.7 Hz, *J* = 9.8 Hz, *J* = 9.8 Hz, *J* = 11.8 Hz, *J* = 11.8 Hz, *J* = 11.8 Hz, *J* = 11.8 Hz, 8H, 4 × OC*H_2_*Ph), 4.22–3.94 (m, 8H, 3 × OC*H_2_*CH_3_, H-6, H-4), 3.75–3.56 (m, 3H, H-7, H-7′, H-5), 3.34, 3.27 (2d, *J* = 9.5 Hz, 1H, H-3), 2.92–2.02 (m, 4H, H-1, PC*H_2_*P, H-1′), 1.37–1.20 (m, 9H, 3 × OCH_2_C*H_3_*); ^13^C NMR (126 MHz, CDCl_3_) *δ* 138.8, 138.7, 138.4, 138.3, 138.2, 138.1 (4 × Cq aromatic), 128.62, 128.58, 128.56, 128.53, 128.52, 128.51, 128.49, 128.04, 128.03, 128.00, 127.94, 127.92, 127.90, 127.87, 127.86, 127.82, 127.80, 127.76, 127.70 (20 × *C*H aromatic), 97.7, 97.3 (2d, *J* = 8.4 Hz, *J* = 6.8 Hz, C-2), 83.7, 83.6 (2d, *J* = 11.1 Hz, *J* = 10.0 Hz, C-3), 83.2 (2d, *J* = 3.0 Hz, *J* = 3.6 Hz, C-4), 79.0, 78.6 (C-5), 75.8, 75.7, 75.6, 75.5, 75.0, 73.5, 73.2 (4 × O*C*H_2_Ph), 71.2, 70.6 (C-6), 69.4, 69.1 (C-7), 62.8, 62.7, 62.6, 62.3, 62.1, 61.4 (6d, *J* = 6.4 Hz, *J* = 6.4 Hz, *J* = 6.4 Hz, *J* = 6.5 Hz, *J* = 6.3 Hz, *J* = 6.6 Hz, 3 × O*C*H_2_CH_3_), 37.5, 34.8 (2d, *J* = 91.7 Hz, *J* = 91.3 Hz, C-1), 30.2, 29.3 (2dd, *J* = 133.8, 86.3 Hz, *J* = 134.1, 85.4 Hz, P*C*H_2_P), 16.6–16.3 (m, 3 × OCH_2_*C*H_3_); ^31^P NMR (202 MHz, CDCl_3_) *δ* 48.3, 45.8 (2d, *J* = 11.6 Hz, *J* = 6.6 Hz, CH_2_*P*CH_2_P), 19.6, 19.5 (2d, *J* = 11.6 Hz, *J* = 6.6 Hz, CH_2_PCH_2_*P*); ESI-MS: Calcd for C_42_H_54_NaO_11_P_2_: 819.8 [M + Na]^+^ Found: 819.1; ESI-HRMS: Calcd for C_42_H_55_O_11_P_2_: 797.3214 [M + H]^+^ Found: 797.3228.

#### 3.1.4. 3-Acetamido-4,5,7-tri-*O*-benzyl-1,3-di-deoxy-1-{[(diethoxyphosphoryl)methyl](ethoxy) phosphoryl}-α-d-*gluco*-2-heptulopyranose **17**

A solution of diethyl {[ethoxy(methyl)phosphoryl]methyl}phosphonate **14** (1.845 g, 7.15 mmol) in anhydrous THF (9 mL) under argon atmosphere was cooled by a dry ice/acetone bath. To this solution was added dropwise a solution of *n*-butyllithium (1.6 M in hexanes, 7.6 mL, 12.25 mmol) during 20 min. After stirring during two hours at −78 °C, a solution of 2-acetamido-3,4,5-tri-*O*-benzyl-2-deoxy-*glucono*-1,5-lactone **13** (1 g, 2.04 mmol) in anhydrous THF (6 mL) was added dropwise to the reaction mixture. After stirring during two hours at −78 °C, the reaction was quenched by addition of a solution of acetic acid in THF (11% *v*/*v* in THF, 4.5 mL). The reaction mixture was diluted with 45 mL of ethyl acetate and 45 mL of water. To this mixture was added 12 g of sodium chloride. The organic layer was collected and the aqueous layer was extracted with 45 mL of ethyl acetate. The organic layers were combined, dried over anhydrous sodium sulfate, filtered, and concentrated. Column chromatography (silica gel 150 mL, EtOAc/EtOH, 95/5 to 9/1, *v*/*v*) of the residue afforded the product **17** as a mixture of stereoisomers (387 mg, 517 µmol, 25%) as a colorless oil. ^1^H NMR (500 MHz, CDCl_3_) *δ* 7.39–7.14 (m, 15H, H aromatic), 6.42, 6.35 (2s, 1H, C-O*H*), 5.58, 5.44 (2d, *J* = 10.0 Hz, *J* = 10.1 Hz, 1H, N*H*COCH_3_), 4.83, 4.82, 4.81, 4.64, 4.56, 4.55, 4.55, 4.49, 4.48, 4.45 (10d, *J* = 10.9 Hz, *J* = 11.5 Hz, *J* = 10.9 Hz, *J* = 11.5 Hz, *J* = 10.9 Hz, *J* = 10.9 Hz, *J* = 12.0 Hz, *J* = 12.0 Hz, *J* = 12.0 Hz, *J* = 12.0 Hz, 6H, 3 × OC*H_2_*Ph), 4.21–3.98 (m, 8H, 3 × OC*H_2_*CH_3_, H-6, H-3), 3.77–3.59 (m, 4H, H-7, H-7′, H-4, H-5), 2.92–2.32 (m, 3H, PC*H_2_*P, H-1), 2.21, 2.12 (t, dd, *J* = 14.7 Hz, *J* = 19.7, 15.3 Hz, 1H, H-1′), 1.88, 1.86 (2s, 3H, NHCOC*H_3_*), 1.38–1.23 (m, 9H, 3 × OCH_2_C*H_3_*); ^13^C NMR (126 MHz, CDCl_3_) *δ* 170.3, 170.1 (NH*C*OCH_3_), 138.7, 138.6, 138.3, 138.21, 138.17, 138.1 (3 × Cq aromatic), 128.62, 128.57, 128.56, 128.55, 128.52, 128.2, 128.13, 128.11, 127.93, 127.89, 127.81, 127.77 (15 × *C*H aromatic), 98.1, 97.4 (2d, *J* = 9.2 Hz, *J* = 6.5 Hz, C-2), 80.9, 80.8 (2d, *J* = 3.7 Hz, *J* = 3.0 Hz, C-4), 78.7, 75.2, 75.0, 73.4, 73.3 (3 × O*C*H_2_Ph), 71.3, 71.0 (C-6), 69.3, 69.1 (C-7), 63.0, 62.8, 62.6, 62.4, 61.7 (5d, *J* = 6.3 Hz, *J* = 6.5 Hz, *J* = 6.5 Hz, *J* = 6.3 Hz, *J* = 6.3 Hz, O*C*H_2_CH_3_), 57.4, 57.2 (2d, *J* = 11.2 Hz, *J* = 11.7 Hz, C-3), 37.0, 35.6 (2d, *J* = 90.3 Hz, *J* = 94.0 Hz, C-1), 30.1, 29.2 (2dd, *J* = 134.7, 88.3 Hz, *J* = 133.7, 83.4 Hz, P*C*H_2_P), 23.63, 23.61 (NHCO*C*H_3_), 16.6–16.3 (m, 3 × OCH_2_*C*H_3_); ^31^P NMR (202 MHz, CDCl_3_) *δ* 48.2, 46.5 (2d, *J* = 12.5 Hz, *J* = 1.8 Hz, CH_2_*P*CH_2_P), 19.4, 19.1 (2d, *J* = 12.5 Hz, *J* = 1.8 Hz, CH_2_PCH_2_*P*); ESI-MS: Calcd for C_37_H_51_NNaO_11_P_2_: 770.3 [M + Na]^+^ Found: 770.1; ESI-HRMS: Calcd for C_37_H_52_NO_11_P_2_: 748.3010 [M + H]^+^ Found: 748.3036.

#### 3.1.5. 2,6-Anhydro-3,4,5,7-tetra*-O*-benzyl-1-deoxy-1-{[(diethoxyphosphoryl)methyl](ethoxy) phosphoryl}-d-*manno*-hept-1-enitol **18**

To a solution of heptulopyranose **15** (0.9 g, 1.13 mmol) in anhydrous dichlormethane (35 mL) under argon atmopshere and cooled by an ice bath, was added dropwise pyridine (3.5 mL, 43.3 mmol) then trifluoroacetic anhydride (0.66 mL, 4.75 mmol). After stirring three hours at 0 °C, the reaction is quenched by the addition of an aqueous saturated solution of sodium bicarbonate (70 mL). The organic layer was collected and the aqueous layer was extracted two times with 50 mL of dichloromethane. The organic layers were combined, washed with 70 mL of an aqueous solution of hydrochloric acid 1N, then 70 mL of brine, dried over anhydrous sodium sulfate, filtered, and concentrated. Column chromatography (silica gel 100 mL, EtOAc/EtOH, 98/2 to 9/1, *v*/*v*) of the residue afforded three fractions of diastereoisomers **18** isolated as colorless oils (94 mg, 120 µmol, 10%; 77 mg, 99 µmol, 9%; 537 mg, 689 µmol, 61%). The physicochemical characteristics of the major fraction are reported. ^1^H NMR (500 MHz, CDCl_3_) *δ* 7.43–7.16 (m, 20H, H aromatic), 5.20 (2d, *J* = 11.9 Hz, *J* = 9.7 Hz, 1H, H-1), 4.79–4.45 (m, 8H, 4 × OC*H_2_*Ph), 4.21–4.06 (m, 1H, H-3), 4.16–3.91 (m, 9H, 3 × POC*H_2_*CH_3_, H-2, H-5, H-6), 3.85–3.66 (m, 3H, H-4, H-7, H-7′), 2.85–2.52 (m, 2H, P*CH_2_*P), 1.30–1.23 (m, 9H, 3 × POCH_2_C*H_3_*); ^13^C NMR (126 MHz, CDCl_3_) *δ* 164.6, 164.0 (s, d, *J* = 1.3 Hz, C-2), 138.2, 138.1, 138.0, 137.95, 137.91, 137.8, 137.42, 137.36 (4 × Cq aromatic), 128.64, 128.60, 128.55, 128.54, 128.50, 128.2, 128.1, 128.03, 128.00, 127.94, 127.92, 127.82, 127.78 (20 × CH aromatic), 100.5, 97.7 (2d, *J* = 136.4 Hz, *J* = 137.5 Hz, C-1), 79.7, 78.9 (C-6), 78.8, 77.9 (C-4), 75.2, 74.6 (2d, *J* = 12.0 Hz, *J* = 12.2 Hz, C-3), 74.9, 74.4 (C-5), 74.2, 73.52, 73.48, 73.4, 72.5, 72.1, 71.6, 71.0 (4 × O*C*H_2_Ph), 69.5 (C-7), 62.5, 62.44, 62.39, 62.3, 61.00, 60.98 (6d, *J* = 6.3 Hz, *J* = 6.3 Hz, *J* = 6.5 Hz, *J* = 6.2 Hz, *J* = 6.1 Hz, *J* = 5.8 Hz, 3 xO*C*H_2_CH_3_), 29.2, 28.9 (2dd, *J* = 134, 92.5 Hz, *J* = 134, 94 Hz, P*C*H_2_P), 16.62, 16.56, 16.48, 16.47, 16.45 (5d, *J* = 7.9 Hz, *J* = 7.1 Hz, *J* = 6.3 Hz, *J* = 5.9 Hz, *J* = 6.4 Hz, OCH_2_*C*H_3_); ^31^P NMR (202 MHz, CDCl_3_) *δ* 31.1, 30.4 (2d, *J* = 3.6 Hz, *J* = 7.8 Hz, CH_2_*P*CH_2_P), 20.5, 20.4 (2d, *J* = 3.6 Hz, *J* = 7.8 Hz, CH_2_*P*CH_2_P); ESI-MS: Calcd for C_42_H_52_NaO_10_P_2_: 801.8 [M + Na]^+^ Found: 801.1; ESI-HRMS: Calcd for C_42_H_53_O_10_P_2_: 779.3108 [M + H]^+^ Found: 779.3140.

#### 3.1.6. 2,6-Anhydro-3,4,5,7-tetra-*O*-benzyl-1-deoxy-1-{[(diethoxyphosphoryl)methyl](ethoxy) phosphoryl}-d-*gluco*-hept-1-enitol **19**

To a solution of heptulopyranose **16** (1.1 g, 1.38 mmol) in anhydrous dichloromethane (41 mL) under argon atmosphere and cooled by an ice bath, was added dropwise pyridine (4.1 mL, 50.7 mmol) then trifluoroacetic anhydride (0.77 mL, 5.52 mmol). After stirring three hours at 0 °C, the reaction is quenched by the addition of an aqueous saturated solution of sodium bicarbonate (80 mL). The organic layer was collected and the aqueous layer was extracted two times with 60 mL of dichloromethane. The organic layers were combined, washed with 80 mL of an aqueous solution of hydrochloric acid 1N, then 80 mL of brine, dried over anhydrous sodium sulfate, filtered, and concentrated. Column chromatography (silica gel 100 mL, EtOAc/EtOH, 98/2 to 9/1, *v*/*v*) of the residue afforded the product **19** as a mixture of stereoisomers (0.75 g, 0.96 mmol, 70%) as a colorless oil. ^1^H NMR (500 MHz, CDCl_3_) *δ* 7.52–6.98 (m, 20H, H aromatic), 5.29, 5.18 (2d, *J* = 11.2 Hz, *J* = 10.1 Hz, 1H, H-1), 4.79–4.49 (m, 8H, 4 × OC*H_2_*Ph), 4.27–3.97 (m, 7H, 3 × OC*H_2_*CH_3_, H-6), 3.97–3.93 (m, 1H, H-3), 3.84–3.76 (m, 3H, H-4, H-5, H-7), 3.73, 3.71 (2dd, *J* = 12.3, 4.1 Hz, *J* = 12.2, 4.0 Hz, 1H, H-7′), 2.93–2.47 (m, 2H, PC*H_2_*P), 1.31–1.25 (m, 9H, 3 × OCH_2_CH_3_); ^13^C NMR (126 MHz, CDCl_3_) *δ* 164.36, 164.2 (d, s, *J* = 1.1 Hz, C-2), 138.0, 137.92, 137.86, 137.8, 137.7, 137.3, 137.2 (4 × Cq aromatic), 128.7, 128.62, 128.56, 128.55, 128.53, 128.50, 128.47, 128.2, 128.13, 128.09, 128.08, 128.05, 128.02, 128.00, 127.98, 127.96, 127.91, 127.90, 127.83, 127.81 (20C, CH aromatic), 99.3, 99.0 (2d, *J* = 136.8 Hz, *J* = 136.9 Hz, C-1), 83.6, 82.9 (C-4), 78.7, 77.9 (2d, *J* = 11.9 Hz, *J* = 12.1 Hz, C-3), 78.2, 77.6 (C-6), 77.4, 77.10 (C-5), 74.3, 73.90, 73.85, 73.56, 73.51, 73.49, 72.8, 72.2 (4 × O*C*H_2_Ph), 68.5, 68.4 (C-7), 62.5, 62.42, 62.35, 62.33, 61.00, 60.98 (6d, *J* = 6.2 Hz, *J* = 6.2 Hz, *J* = 6.3 Hz, *J* = 6.2 Hz, *J* = 6.1 Hz, *J* = 6.1 Hz, 3 × O*C*H_2_*C*H_3_), 29.1 (dd, *J* = 134.4, 93.1 Hz, CHP*C*H_2_P), 16.7–16.4 (m, 3 × OCH_2_*C*H_3_); ^31^P NMR (202 MHz, CDCl_3_) *δ* 30.8, 30.6 (2d, *J* = 7.7 Hz, *J* = 6.5 Hz, CH_2_*P*CH_2_P), 20.5, 20.5 (2d, *J* = 7.7 Hz, *J* = 6.5 Hz, CH_2_PCH_2_*P*); ESI-MS: Calcd for C_42_H_53_O_10_P_2_: 779.8 [M + H]^+^ Found: 779.0, Calcd for C_42_H_54_KO_10_P_2_: 819.9 [M + 2H + K]^+^ Found: 819.2; ESI-HRMS: Calcd for C_42_H_53_O_10_P_2_: 779.3108 [M + H]^+^ Found: 779.3127.

#### 3.1.7. 3-Acetamido-2,6-Anhydro-4,5,7-tri-*O*-benzyl-1,3-di-deoxy-1-{[(diethoxyphosphoryl) methyl](ethoxy)phosphoryl}-d-*gluco*-hept-1-enitol **20**

To a solution of heptulopyranose **17** (340 mg, 455 µmol) in anhydrous dichlormethane (14 mL) under argon atmosphere and cooled by an ice bath, was added dropwise pyridine (1.4 mL, 17.3 mmol) then trifluoroacetic anhydride (250 µL, 1.82 mmol). After stirring two hours at 0 °C, the reaction is quenched by the addition of an aqueous saturated solution of sodium bicarbonate (26 mL). The organic layer was collected and the aqueous layer was extracted two times with 26 mL of dichloromethane. The organic layers were combined, washed with 26 mL of an aqueous solution of hydrochloric acid 1N, then 26 mL of brine, dried over anhydrous sodium sulfate, filtered, and concentrated. Column chromatography (silica gel 30 mL, EtOAc/EtOH, 9/1 to 8/2, *v*/*v*) of the residue afforded the product **20** as a mixture of stereoisomers and rotamers (210 mg, 288 µmol, 63%) as a colorless oil. ^1^H NMR (500 MHz, CDCl_3_) δ 7.52–6.92 (m, 30H, H aromatic), 5.14, 5.09, 5.03, 4.94 (4d, *J* = 11.3 Hz, *J* = 11.4 Hz, *J* = 9.3 Hz, *J* = 11.7 Hz, 1H, H-1), 4.84–4.34 (m, 6H, 3 × OC*H_2_*Ph), 4.29–3.55 (m, 12H, H-6, 3 × OC*H_2_*CH_3_, H-3, H-4, H-5, H-7, H-7′), 3.27–2.23 (m, 2H, PC*H_2_*P), 2.00, 1.97, 1.94, 1.92, 1.91, 1.90, 1.87, (7 s, 3H, NHCOC*H_3_*), 1.36–1.15 (m, 9H, 3 × OCH_2_C*H_3_*); ^31^P NMR (202 MHz, CDCl_3_) δ 31.3, 30.8 (2broad s, CH_2_*P*CH_2_P), 20.7, 20.6, 20.2, 20.1, 19.8, 19.6 (6d, *J* = 4.7 Hz, *J* = 5.7 Hz, *J* = 2.1 Hz, *J* = 2.9 Hz, *J* = 5.8 Hz, *J* = 3.6 Hz, CH_2_PCH_2_*P*); ESI-MS: Calcd for C_37_H_49_NNaO_10_P_2_: 752.7327 [M + Na]^+^ Found: 752.2; ESI-HRMS: Calcd for C_37_H_49_NNaO_10_P_2_: 752.2724 [M + Na]^+^ Found: 752.2730.

#### 3.1.8. 2,6-Anhydro-1-deoxy-1-{[(diethoxyphosphoryl)methyl](ethoxy)phosphoryl}-d-*glycero*-d-*galacto-*heptitol **21**

To a solution of a mixture of the previous d-*manno*-hept-1-enitol **18** (476 mg, 611 µmol) in ethanol (28 mL) under argon atmosphere was added a catalytic amount of palladium 10% on charcoal (140 mg, 132 µmol). Then, the reaction mixture was purged with hydrogen gas and stirred overnight under hydrogen atmosphere. The resulting solution was degassed with argon atmosphere and it was filtered through a celite© (ThermoFisher, Kandel, Germany) patch which was rinsed with 10 mL of ethanol. The concentration under vacuum furnished the product **21** as a mixture of stereoisomers with a R configuration for the C2 stereogenic centre (253 mg, 602 µmol, quant.) as a white solid. ^1^H NMR (500 MHz, MeOD) δ 4.24–4.10 (m, 6H, 3 × POCH_2_CH_3_), 3.94–3.86 (m, 1H, H-1), 3.87, 3.86 (2dd, *J* = 11.7, 2.3 Hz, *J* = 11.3, 2.4 Hz, 1H, H-7), 3.72, 3.71 (2d, *J* = 3.0 Hz, 1H, H-3), 3.66, 3.65 (2dd, *J* = 11.3, 6.7 Hz, *J* = 11.7, 6.3 Hz, 1H, H-7′), 3.53 (2t, *J* = 9.4 Hz, 1H, H-5), 3.49, 3.48 (2dd, *J* = 9.4, 3.0 Hz, 1H, H-4), 3.26, 3.23 (2ddd, *J* = 9.4, 6.3, 2.3 Hz, *J* = 9.4, 6.7, 2.4 Hz, 1H, H-6), 2.97–2.75 (m, 2H, PCH_2_P), 2.61–2.51 (m, 1H, H-1), 2.20–2.02 (m, 1H, H-1′), 1.35, 1.34 (2t, *J* = 7.0 Hz, 9H, 3 × POCH_2_CH_3_); ^13^C NMR (126 MHz, MeOD) δ 82.33, 82.28 (C-6), 76.3 (C-4), 74.7, 74.5 (2d, *J* = 3.7 Hz, *J* = 4.6 Hz, C-2), 73.3, 73.2 (d, *J* = 12.2 Hz, *J* = 11.4 Hz, C-3), 68.6, 68.5 (C-5), 64.2, 64.08, 64.02, 63.98, 62.9, 62.8 (6d, *J* = 6.5 Hz, POCH_2_CH_3_), 63.06, 63.00 (C-7), 33.5, 33.4 (2d, *J* = 99 Hz, C-1), 28.6, 28.4 (2dd, *J* = 135, 83 Hz, PCH_2_P), 16.83, 16.79, 16.7, 16.6 (4d, *J* = 6.3 Hz, 3 × POCH_2_CH_3_); ^31^P NMR (202 MHz, MeOD) δ 48.8, 47.7 (2d, *J* = 11.4 Hz, *J* = 7.5 Hz, CH_2_PCH_2_P), 21.2, 21.0 (2d, *J* = 7.5 Hz, *J* = 11.4 Hz, CH_2_PCH_2_P); ESI-MS: Calcd for C_28_H_59_NaO_20_P_4_: 862.7 [2M – H + Na]^+^ Found: 862.7; ESI-HRMS: Calcd for C_14_H_30_NaO_10_P_2_: 443.1206 [M + Na]^+^ Found: 443.1223.

#### 3.1.9. 2,6-Anhydro-1-deoxy-1-{[(diethoxyphosphoryl)methyl](ethoxy)phosphoryl}-d-*glycero*-d-*gulo*-heptitol **22**

To a solution of a mixture of the d-*gulo*-hept-1-enitol **19** (720 mg, 925 µmol) in ethanol (42 mL) under argon atmosphere was added a catalytic amount of palladium 10% on charcoal (210 mg, 190 µmol). Then, the reaction mixture was purged with hydrogen gas and stirred overnight under hydrogen atmosphere. The resulting solution was degassed with argon atmosphere and it was filtered through a celite© patch. The concentration under vacuum furnished the product **22** as a mixture of stereoisomers with a R configuration for the C2 stereogenic centre (377 mg, 897 µmol, 97%) as a white solid. ^1^H NMR (500 MHz, MeOD) *δ* 4.24–4.08 (m, 6H, 3 × OC*H_2_*CH_3_), 3.87, 3.86 (2dd, *J* = 11.9, 2.2 Hz, *J* = 11.8, 1.4 Hz, 1H, H-7), 3.64–3.50 (m, 2H, H-7′, H-2), 3.38–3.22 (m, 3H, H-4, H-6, H-5), 3.11, 3.08 (2t, *J* = 9.0 Hz, *J* = 9.1 Hz, 1H, H-3), 2.99–2.74 (m, 2H, PC*H_2_*P), 2.57–2.40 (m, 1H, H-1), 2.26–2.16 (m, 1H, H-1′), 1.41–1.28 (m, 9H, 3 × OCH_2_C*H_3_*); ^13^C NMR (126 MHz, MeOD) *δ* 82.04, 81.96 (C-6), 79.44, 79.42 (d, *J* = 3,8 Hz, *J* = 4.0 Hz, C-4), 76.2, 76.1 (d, *J* = 5.3 Hz, *J* = 6.0 Hz, C-2), 75.9, 75.7 (d, *J* = 13.7 Hz, *J* = 12.7 Hz, C-3), 71.9, 71.8 (C-5), 64.1, 64.03, 63.99, 63.65, 62.81, 62.75 (6d, *J* = 6.4 Hz, *J* = 6.3 Hz, *J* = 6.3 Hz, *J* = 6.3 Hz, *J* = 6.5 Hz, *J* = 6.6 Hz, O*C*H_2_CH_3_), 63.0, 62.9 (C-7), 33.3, 32.9 (2d, *J* = 99.1 Hz, *J* = 99.2 Hz, C-1), 29.1, 28.5 (2dd, *J* = 135.0, 24.4 Hz, *J* = 135.0, 24.9 Hz, P*C*H_2_P), 16.9–16.53 (m, 3 × OCH_2_*C*H_3_); ^31^P NMR (202 MHz, MeOD) *δ* 47.2, 46.3 (2d, *J* = 11.4 Hz, *J* = 7.9 Hz, CH_2_*P*CH_2_P), 19.8, 19.6 (d, *J* = 7.9 Hz, *J* = 11.4 Hz, CH_2_PCH_2_*P*); ESI-MS: Calcd for C_28_H_59_NaO_20_P_4_: 862.7 [2M – H + Na]^+^ Found: 862.7; ESI-HRMS: Calcd for C_14_H_31_O_10_P_2_: 421.1387 [M + H]^+^ Found: 421.1394.

#### 3.1.10. 3-Acetamido-2,6-anhydro-1,3-di-deoxy-1-{[(diethoxyphosphoryl)methyl](ethoxy) phosphoryl}-d-glycero-d-gulo-heptitol 23

To a solution of a mixture of the d-*gluco*-hept-1-enitol **20** (210 mg, 288 µmol) in ethanol (14 mL) under argon atmosphere was added a catalytic amount of palladium 10% on charcoal (70 mg, 66 µmol). Then, the reaction mixture was purged with hydrogen gas and stirred overnight under hydrogen atmosphere. The resulting solution was degassed with argon atmosphere and it was filtered through a celite© patch. The concentration under vacuum furnished the product **23** as a mixture of stereoisomers with a R configuration for the C2 stereogenic centre (132 mg, 288 µmol, quant.) as a white solid. ^1^H NMR (500 MHz, MeOD) *δ* 4.23–4.10 (m, 6H, 3 × OC*H_2_*CH_3_), 3.91–3.85 (m, 1H, H-7), 3.70–3.56 (m, 3H, H-7′, H-3, H-2), 3.45–3.39 (m, 1H, H-4), 3.34–3.25 (m, 1H, H-5), 3.26 (ddd, *J* = 9.7, 5.8, 2.2 Hz, 1H, H-6), 3.05–2.71 (m, 2H, PC*H_2_*P), 2.30–2.18 (m, 1H, H-1), 2.15–2.06 (m, 1H, H-1′), 2.00 (s, 3H, NHCOC*H_3_*), 1.37–1.32 (m, 9H, 3 × OCH_2_C*H_3_*); ^13^C NMR (126 MHz, MeOD) *δ* 173.93, 173.89 (NH*C*OCH_3_), 82.0, 81.9 (C-6), 76.8 (2d, *J* = 2.7 Hz, *J* = 2.4 Hz, C-4), 75.4, 75.0 (2d, *J* = 5.0 Hz, *J* = 6.3 Hz, C-2), 72.23, 72.15 (C-5), 64.1, 64.04, 63.98, 63.93, 62.85, 62.84 (6d, *J* = 6.3 Hz, *J* = 6.9 Hz, *J* = 6.3 Hz, *J* = 6.1 Hz, *J* = 7.7 Hz, *J* = 7.7 Hz, 3 × O*C*H_2_CH_3_), 62.92, 62.88 (C-6), 57.7, 57.6 (d, *J* = 13.6 Hz, *J* = 14.2 Hz, C-3), 33.8, 33.4 (2d, *J* = 99.4 Hz, *J* = 100.2 Hz, C-1), 28.7, 28.5 (2dd, *J* = 134.8, 82.5 Hz, *J* = 134.8, 83.2 Hz, P*C*H_2_P), 23.0, 22.9 (NHCO*C*H_3_), 16.81, 16.76, 16.64, 16.63 (4d, *J* = 6.2 Hz, *J* = 6.3 Hz, *J* = 6.3 Hz, *J* = 6.1 Hz, 3 × OCH_2_*C*H_3_); ^31^P NMR (202 MHz, MeOD) *δ* 48.2, 47.1 (2d, *J* = 12.0 Hz, *J* = 7.1 Hz, CH_2_*P*CH_2_P), 21.3, 21.0 (2d, *J* = 7.1 Hz, *J* = 12.0 Hz, CH_2_PCH_2_*P*); ESI-MS: Calcd for C_32_H_65_N_2_NaO_20_P_4_: 944.8 [2M − H + Na]^+^ Found: 944.7, Calcd for C_16_H_33_NNaO_10_P_2_: 484.4 [M + Na]^+^ Found: 484.1; ESI-HRMS: Calcd for C_16_H_34_O_10_NP_2_: 462.1662 [M + H]^+^ Found: 462.1667.

#### 3.1.11. 3,4,5,7-Tetra-*O*-acetyl-2,6-anhydro-1-deoxy-1-{[(diethoxyphosphoryl)methyl](ethoxy) phosphoryl}-d-*glycero*-d-*galacto*-heptitol **24**

To a solution of a mixture of the previous d-*glycero*-d-*galacto*-heptitol **21** (235 mg, 559 µmol) in pyridine (9 mL) cooled by an ice bath, was added dropwise acetic anhydride (4.5 mL, 48 mmol). The reaction mixture was allowed to warm to room temperature. After stirring overnight at room temperature, the reaction is stopped by evaporation of the reactants. Column chromatography (silica gel 60 mL, CH_2_Cl_2_/EtOH, 95/5 to 9/1, *v*/*v*) of the residue afforded **24** as a mixture of stereoisomers with a R configuration for the C2 stereogenic centre (265 mg, 450 µmol, 80%) as a colorless oil. ^1^H NMR (500 MHz, CDCl_3_) *δ* 5.32, 5.31 (2broad d, *J* = 3.4 Hz, *J* = 3.6 Hz, 1H, H-3), 5.17, 5.15 (2t, *J* = 10.0 Hz, 1H, H-5), 5.07, 5.06 (2dd, *J* = 10.0, 3.6 Hz, *J* = 10.0, 3.4 Hz, 1H, H-4), 4.25–4.07 (m, 9H, H-2, 3 × POC*H_2_*CH_3_, H-7, H-7′), 3.75, 3.71 (2ddd, *J* = 10.0, 7.0, 2.3 Hz, *J* = 10.0, 6.3, 2.4 Hz, 1H, H-6), 2.70–2.35 (m, 3H, 1.5 × PC*H_2_*P, H-1), 2.17, 2.08, 2.04, 1.96 (4s, 12H, 4 × C*H_3_*CO), 2.06–1.77 (m, 1H, H-1′), 1.38–1.28 (m, 9H, 3 × POCH_2_C*H_3_*); ^13^C NMR (126 MHz, CDCl_3_) *δ* 170.7, 170.49, 170.47, 170.1, 170.0, 169.92, 169.85 (4 × CH_3_*C*O), 76.6, 76.4 (C-6), 72.4, 72.16 (2d, *J* = 2.7 Hz, *J* = 3.4 Hz, C-2), 72.17, 72.10 (2d, *J* = 1.7 Hz, *J* = 2.0 Hz, C-4), 70.62, 70.55 (2d, *J* = 12.9 Hz, *J* = 12.4 Hz, C-3), 66.1, 66.0 (C-5), 63.2, 63.1 (C-7), 62.8, 62.62, 62.60, 62.36, 61.58, 61.56 (6d, *J* = 6.5 Hz, 3 × PO*C*H_2_CH_3_), 32.2, 31.1 (2d, *J* = 98.5 Hz, *J* = 99.7 Hz, C-1), 29.3, 28.9 (2dd, *J* = 134.5, 83.1 Hz, *J* = 134.6, 81.9 Hz, P*C*H*_2_*P), 20.86, 20.84, 20.83, 20.69, 20.68 (4 × *C*H_3_CO), 16.8–16.4 (m, 3 × POCH_2_*C*H_3_); ^31^P NMR (202 MHz, CDCl_3_) *δ* 44.1, 42.2 (2d, *J* = 9.8 Hz, *J* = 5.9 Hz, CH_2_*P*CH_2_P), 19.9, 19.7 (d, *J* = 9.8 Hz, *J* = 5.9 Hz, CH_2_PCH_2_*P*); ESI-MS: Calcd for C_44_H_75_NaO_28_P_4_: 1198.9 [2M – H + Na]^+^ Found: 1198.4; ESI-HRMS: Calcd for C_22_H_39_O_14_P_2_: 589.1810 [M + H]^+^ Found: 589.1818.

#### 3.1.12. 3,4,5,7-Tetra-*O*-acetyl-2,6-anhydro-1-deoxy-1-{[(diethoxyphosphoryl)methyl](ethoxy) phosphoryl}-d-*glycero*-d-*gulo*-heptitol **25**

To a solution of a mixture of the previous d-*glycero*-d-*gulo*-heptitol **22** (372 mg, 885 µmol) in pyridine (15 mL) cooled by an ice bath, was added dropwise acetic anhydride (7.5 mL, 79 mmol). The reaction mixture was allowed to warm to room temperature. After stirring overnight at room temperature, the reaction is stopped by evaporation of the reactants. Column chromatography (silica gel 80 mL, CH_2_Cl_2_/EtOH, 95/5 to 9/1, *v*/*v*) of the residue afforded **25** as a mixture of stereoisomers with a R configuration for the C2 stereogenic centre (503 mg, 854 µmol, 96%) as a colorless oil. ^1^H NMR (500 MHz, CDCl_3_) *δ* 5.14, 5.13 (2t, *J* = 9.6 Hz, 1H, H-4), 4.98, 4.96 (2t, *J* = 9.6 Hz, 1H, H-5), 4.87, 4.86 (2t, *J* = 9.6 Hz, 1H, H-3), 4.20–4.00 (m, 8H, 3 × OC*H_2_*CH_3_, H-7, H-7′), 3.99–3.81 (m, 1H, H-2), 3.74, 3.69 (2ddd, *J* = 10.0, 6.3, 2.2 Hz, 1H, *J* = 10.0, 5.6, 2.2 Hz, H-6), 2. 70–2.39 (m, 2H, PC*H_2_*P), 2.32–1.86 (m, 2H, H-1, H-1′), 2.03, 2.01, 2.00, 1.98, 1.95, 1.94 (6s, 12H, 4 × C*H_3_*CO), 1.34–1.25 (m, 9H, 3 × OCH_2_C*H_3_*); ^13^C NMR (126 MHz, CDCl_3_) *δ* 170.5, 170.3, 170.2, 169.7, 169.6, 169.5 (4 × CH_3_*C*O), 76.0, 75.8 (C-6), 74.0, 73.9 (2d, *J* = 2.2 Hz, *J* = 1.7 Hz, C-4), 73.4, 73.2 (2d, *J* = 4.0 Hz, *J* = 6.5 Hz, C-2), 72.2, 72.1 (d, *J* = 14.6 Hz, *J* = 13.8 Hz, C-3), 68.6, 68.5 (C-5), 62.7, 62.5, 62.4, 62.3, 61.4 (5d, *J* = 6.3 Hz, *J* = 6.1 Hz, *J* = 4.5 Hz, *J* = 6.4 Hz, Hz, *J* = 6.5 Hz, 3 × O*C*H_2_CH_3_), 62.5, 62.4 (C-7), 32.4, 31.5 (2d, *J* = 98.0 Hz, *J* = 100.0 Hz, C-1), 29.5, 28.9 (2dd, *J* = 134.5, 83.8 Hz, *J* = 134.9, 81.7 Hz, P*C*H_2_P), 20.7, 20.6 (4 × *C*H_3_CO), 16.6–16.3 (m, 3 × OCH_2_*C*H_3_); ^31^P NMR (202 MHz, CDCl_3_) *δ* 43.9, 42.1 (2d, *J* = 9.0 Hz, *J* = 3.1 Hz, CH_2_*P*CH_2_P), 19.9, 19.7 (2d, *J* = 9.0 Hz, *J* = 3.1 Hz, CH_2_PCH_2_*P*); ESI-MS: Calcd for C_44_H_75_NaO_28_P_4_: 1198.9 [2M – H + Na]^+^ Found: 1198.4; ESI-HRMS: Calcd for C_22_H_39_O_14_P_2_: 589.1810 [M + H]^+^ Found: 589.1820.

#### 3.1.13. 3-Acetamido-4,5,7-tri-*O-*acetyl-2,6-anhydro-1,3-di-deoxy-1-{[(diethoxyphosphoryl) methyl](ethoxy)phosphoryl}-d-*glycero*-d-*gulo*-heptitol **26**

To a solution of a mixture of the previous d-*glycero*-d-*gulo*-heptitol **23** (132 mg, 288 µmol) in pyridine (5 mL) cooled by an ice bath, was added dropwise acetic anhydride (2.5 mL, 26.4 mmol). The reaction mixture was allowed to warm to room temperature. After stirring overnight at room temperature, the reaction is stopped by evaporation of the reactants. Column chromatography (silica gel 30 mL, CH_2_Cl_2_/EtOH, 95/5 to 9/1, *v*/*v*) of the residue afforded **26** as a mixture of stereoisomers with a R configuration for the C2 stereogenic centre (124 mg, 211 µmol, 73%) as a colorless oil. ^1^H NMR (500 MHz, CDCl_3_) *δ* 6.27, 6.20 (2d, *J* = 9.3 Hz, *J* = 9.5 Hz, 1H, N*H*COCH_3_), 5.09–4.98 (m, 2H, H-5, H-4), 4.25–3.99 (m, 9H, 3 × OC*H_2_*CH_3_, H-7, H-7′, H-3), 3.92, 3.86 (2ddd, *J* = 11.9, 9.5, 2.2 Hz, *J* = 12.1, 9.3, 2.2 Hz, 1H, H-2), 3.75–3.67 (m, 1H, H-6), 2.66–2.01 (m, 4H, H-1, H-1′, PC*H_2_*P), 2.07, 2.06, 2.02, 2.01, 2.00 (5s, 9H, 3 × OCOC*H_3_*), 1.94 (s, 3H, NHCOC*H_3_*), 1.38–1.30 (m, 9H, 3 × OCH_2_C*H_3_*); ^13^C NMR (126 MHz, CDCl_3_) *δ* 171.4, 171.3, 170.75, 170.74, 170.67, 170.6, 169.5 (3 × O*C*OCH_3_, NH*C*OCH_3_), 76.1, 75.9 (C-6), 74.7, 74.0 (2d, *J* = 3.6 Hz, *J* = 5.8 Hz, C-2), 74.33, 74.29 (2d, *J* = 2.4 Hz, *J* = 3.4 Hz, C-4), 68.7, 68.6 (C-5), 63.0, 62.64, 62.56, 62.54, 61.5, 61.4 (6d, *J* = 6.5 Hz, 3 × O*C*H_2_CH_3_), 62.8, 62.52 (C-7), 54.31, 54.25 (2d, *J* = 13.5 Hz, *J* = 14.8 Hz, C-3), 32.8, 32.0 (d, *J* = 98.1 Hz, *J* = 99.4 Hz, C-1), 29.3, 28.7 (2dd, *J* = 134.5, 83.6 Hz, *J* = 135.1, 81.6 Hz, CH_2_P*C*H_2_P), 23.3 (NHCO*C*H_3_), 20.84, 20.81, 20.79, 20.74, 20.73 (3 × OCO*C*H_3_), 16.7–16.4 (3 × O*C*H_2_*C*H_3_); ^31^P NMR (202 MHz, CDCl_3_) *δ* 44.8, 43.0 (2d, *J* = 8.0 Hz, *J* = 1.4 Hz, CH_2_*P*CH_2_P), 19.8, 19.6 (2d, *J* = 8.0 Hz, *J* = 1.4 Hz, CH_2_PCH_2_*P*); ESI-MS: Calcd for C_22_H_39_NNaO_13_P_2_: 610.5 [M + Na]^+^ Found: 610.1, Calcd for C_44_H_77_N_2_NaO_26_P_4_: 1197.0 [2M − H + Na]^+^ Found: 1196.5; ESI-HRMS: Calcd for C_22_H_40_NO_13_P_2_: 588.1969 [M + H]^+^ Found: 588.1991.

#### 3.1.14. Sodium {[oxido(2,6-anhydro-1-deoxy-d-*glycero*-d-*galacto*-heptityl)phosphoryl]methyl} phosphonate **27**

To a solution of a mixture of the previous d-*glycero*-d-*galacto*-heptitol **24** (53.5 mg, 90 µmol) in anhydrous dichloromethane (2 mL) cooled by an ice bath, was added dropwise bromotrimethylsilane (142 µL, 1.08 mmol). The reaction mixture was allowed to warm to room temperature. After stirring overnight at room temperature, the reaction is stopped by evaporation of the reactants. Then the residue was dissolved in water (4 mL). Sodium hydoxide (28.8 mg, 720 µmol) was added to the reaction mixture. After 16 h of stirring at room temperature, the reaction was stopped with addition of cation exchange resin (DOWEX© 50WX8 resin, H^+^ form, ACROS, Geel, Belgium). The resin was removed by filtration through a millipore filter (45 µm, AIT, Houilles, France). The aqueous solution was freeze dried. The resulting solid (48 mg) was solubilized in water (0.48 mL). To this solution was added absolute ethanol (4.8 mL). After 16 h at room temperature, the resulting solid was recovered and rinsed with absolute ethanol. It was solubilized in water and freeze dried to afford the phosphonate **27** as a white solid (34.6 mg, 86 µmol, 95%). ^1^H NMR (500 MHz, CDCl_3_) *δ* 4.03 (td, *J* = 9.3, 3.9 Hz, 1H, H-2), 3.97 (dd, *J* = 12.3, 2.0 Hz, 1H, H-7), 3.94 (d, *J* = 3.2 Hz, 1H, H-3), 3.78 (dd, *J* = 12.3, 6.3 Hz, 1H, H-7′), 3.76 (dd, *J* = 9.6, 3.2 Hz, 1H, H-4), 3.63 (t, *J* = 9.6 Hz, 1H, H-5), 3.45 (ddd, *J* = 9.6, 6.3, 2.0 Hz, 1H, H-6), 2.57–2.34 (m, 3H, PC*H_2_*P, H-1), 2.14 (td, *J* = 16.3, 3.9 Hz, 1H, H-1′); ^13^C NMR (126 MHz, CDCl_3_) *δ* 80.0 (C-6), 74.2 (C-4), 73.6 (d, *J* = 2.8 Hz, C-2), 71.8 (d, *J* = 10.5 Hz, C-3), 67.0 (C-5), 61.3 (C-7), 32.2 (d, *J* = 96.5 Hz, C-1), 30.9 (dd, *J* = 122.1, 80.7 Hz, P*C*H_2_PO); ^31^P NMR (202 MHz, CDCl_3_) δ 36.05 (d, *J* = 4.5 Hz, CH_2_*P*CH_2_P), 11.75–11.65 (m, CH_2_PCH_2_*P*); ^31^P NMR non decoupled (202 MHz, CDCl_3_) *δ* 36.50–35.73 (m, CH_2_*P*CH_2_P), 11.70 (td, *J* = 18.5, 4.5 Hz, CH_2_PCH_2_*P*); ESI-MS (mobile phase gradient ACN/H_2_O + 0.1% formic acid): Calcd for C_8_H_19_O_10_P_2_: 337.0 [M + H]^+^ Found: 337.0; ESI-HRMS: Calcd for C_8_H_19_O_10_P_2_: 337.0453 [M + H]^+^ Found: 337.0443.

#### 3.1.15. Sodium {[oxido(2,6-anhydro-1-deoxy-d-*glycero*-d-*gulo*-heptityl)phosphoryl]methyl} phosphonate **28**

To a solution of a mixture of the previous d-*glycero*-d-*gulo*-heptitol **25** (63.5 mg, 116 µmol) in anhydrous dichloromethane (2 mL) cooled by an ice bath, was added dropwise bromotrimethylsilane (183 µL, 1.39 mmol). The reaction mixture was allowed to warm to room temperature. After stirring overnight at room temperature, the reaction is stopped by evaporation of the reactants. Then the residue was dissolved in water (4 mL). Sodium hydoxide (37 mg, 925 µmol) was added to the reaction mixture. After 16 h of stirring at room temperature, the reaction was stopped with addition of cation exchange resin (DOWEX© 50WX8 resin, H^+^ form). The resin was removed by filtration through a millipore filter (45 µm). The aqueous solution was freeze dried. The resulting solid (62 mg) was solubilized in water (0.62 mL). To this solution was added absolute ethanol (6.2 mL). After 16 h at room temperature, the resulting solid was recovered and rinsed with absolute ethanol. It was solubilized in water and freeze dried to afford the phosphonate **28** as a white solid (43 mg, 107 µmol, 92%). ^1^H NMR (500 MHz, D_2_O) *δ* 3.93 (d, *J* = 12.3 Hz, 1H, H-7), 3.74 (dd, *J* = 12.3, 4.3 Hz, 1H, H-7′), 3.68 (qd, *J* = 9.4, 2.5 Hz, 1H, H-2), 3.52 (t, *J* = 9.4 Hz, 1H, H-4), 3.48–3.40 (m, 2H, H-5, H-6), 3.26 (t, *J* = 9.4 Hz, 1H, H-3), 2.33–2.01 (m, 4H, H-1, H-1′, PC*H_2_*P); ^13^C NMR (126 MHz, D_2_O) *δ* 79.5 (C-6), 77.2 (C-4), 75.6 (d, *J* = 4.3 Hz, C-2), 74.5 (d, *J* = 11.2 Hz, C-3), 69.8 (C-5), 60.9 (C-7), 33.71 (d, *J* = 96.0 Hz, C-1), 32.38 (dd, *J* = 119.5, 79.8 Hz, P*C*H_2_PO); ^31^P NMR (202 MHz, D_2_O) *δ* 34.72 (d, *J* = 6.1 Hz, CH_2_*P*CH_2_P), 15.25 (d, *J* = 6.1 Hz, CH_2_PCH_2_*P*); ^31^P NMR non decoupled (202 MHz, D_2_O) *δ* 35.97–32.61 (m, CH_2_*P*CH_2_P), 15.22 (td, *J* = 19.3, 6.1 Hz, CH_2_PCH_2_*P*); ESI-MS (mobile phase gradient ACN/H_2_O + 0.1% formic acid): Calcd for C_8_H_19_O_10_P_2_: 337.0 [M + H]^+^ Found: 337.0; ESI-HRMS: Calcd for C_8_H_19_O_10_P_2_: 337.0453 [M + H]^+^ Found: 337.0448.

#### 3.1.16. Sodium {[oxido(3-acetamido-2,6-anhydro-1,3-di-deoxy-d-*glycero*-d-*gulo*-heptityl) phosphoryl]methyl}phosphonate **29**

To a solution of a mixture of the previous d-*glycero*-d-*gulo*-heptitol 26 (51 mg, 87 µmol) in anhydrous dichloromethane (2 mL) cooled by an ice bath, was added dropwise bromotrimethylsilane (137 µL, 1.04 mmol). The reaction mixture was allowed to warm to room temperature. After stirring overnight at room temperature, the reaction is stopped by evaporation of the reactants. Then the residue was dissolved in water (4 mL). Sodium hydoxide (27.8 mg, 695 µmol) was added to the reaction mixture. After 16 h of stirring at room temperature, the reaction was stopped with addition of cation exchange resin (DOWEX© 50WX8 resin, H^+^ form). The resin was removed by filtration through a millipore filter (45 µm). The aqueous solution was freeze dried. The resulting solid (51 mg) was solubilized in water (0.51 mL). To this solution was added absolute ethanol (5.1 mL). After 16 h at room temperature, the resulting solid was recovered and rinsed with absolute ethanol. It was solubilized in water and freeze dried to afford the phosphonate **29** as an orange solid (32.9 mg, 74 µmol, 85%). ^1^H NMR (500 MHz, D_2_O) *δ* 3.95 (d, *J* = 12.4 Hz, 1H, H-7), 3.78 (dd, *J* = 12.4, 5.1 Hz, 1H, H-7′), 3.77–3.71 (m, 1H, H-2), 3.67 (t, *J* = 9.3 Hz, 1H, H-3), 3.56 (t, *J* = 9.3 Hz, 1H, H-4), 3.50 (t, *J* = 9.3 Hz, 1H, H-5), 3.45 (dd, *J* = 9.3, 5.1 Hz, 1H, H-6), 2.27 (dt, *J* = 19.2, 15.7 Hz, 1H, 0.5 × PC*H_2_*P), 2.19–2.05 (m, 1H, 0.5 × PC*H_2_*P), 2.10 (s, 3H, NHCOC*H_3_*), 2.05–1.89 (m, 2H, H-1, H-1′); ^13^C NMR (126 MHz, D_2_O) *δ* 174.7 (NH*C*OCH_3_), 79.4 (C-6), 75.4 (C-4), 74.5 (d, *J* = 5.1 Hz, C-2), 69.9 (C-5), 60.8 (C-7), 56.5 (d, *J* = 13.2 Hz, C-3), 33.4 (d, *J* = 96.4 Hz, C-1), 32.0 (dd, *J* = 119.8, 79.3 Hz, P*C*H_2_PO), 22.3 (NHCO*C*H_3_); ^31^P NMR (202 MHz, D_2_O) *δ* 34.08 (d, *J* = 7.5 Hz, CH_2_*P*CH_2_P), 15.76 (d, *J* = 7.5 Hz, CH_2_PCH_2_*P*); ^31^P NMR non decoupled (202 MHz, D_2_O) *δ* 34.3–33.8 (m, CH_2_*P*CH_2_P), 15.74 (td, *J* = 19.4, 7.5 Hz, CH_2_PCH_2_*P*); ESI-MS (mobile phase gradient ACN/H_2_O + 0.1% formic acid): Calcd for C_10_H_22_NO_10_P_2_: 378.1 [M + H]^+^ Found: 378.1; ESI-HRMS: Calcd for C_10_H_22_NO_10_P_2_: 378.0719 [M + H]^+^ Found: 378.0709.

### 3.2. Enzyme Assays

#### 3.2.1. DLODP Assay

Microsomal membranes containing DLODP were prepared from rat liver (APAFIS#20275-2019041612268204 v4) as previously described [46]. Membranes (8.5 mg) were solubilized in 20 mM MES pH 6.2, 200 mM NaCl, 1 mM CoCl_2_, 10% glycerol, 12.5 mg of NP-40 for 1 h at 4 °C. The solution was centrifuged at 100,000 gAV for 45 min at 4 °C. The supernatant containing solubilized DLODP was collected and stored at –150 °C. DLODP assay was performed in a final mixture of 20 mM MES pH 5.5, 150 mM NaCl, 1 mM CoCl_2_, 10% glycerol, 0.2% NP-40, 20 µg of solubilized membrane, metabolically radiolabeled dolichol-linked oligosaccharide (3.0 × 10^4^ cpm), 0.1 mM kifunensin and 0.1 mM swainsonine for 20 min at 37 °C [44]. The reaction was stopped by adding 10 reaction volumes of cold distilled water. The solution was added to coupled Dowex 50WX4 (H^+^ form, Sigma Aldrich, SARL, Saint-Quentin Fallavier, France) and Dowex 1 × 8 (formate form, Sigma Aldrich SARL, Saint-Quentin Fallavier, France) ion-exchangers. The exchangers were washed with 12 column volumes of distilled water, then the Dowex 1 × 8 was eluted with 5 column volumes of 3 M formic acid. The eluate was dried and radioactivity determined by scintillation counting.

#### 3.2.2. Alkaline Phosphatase Assay

Alkaline phosphatase (524572, Sigma Aldrich SARL, Saint-Quentin Fallavier, France) activity was determined by measuring the quantity of [^14^C]glucose released from [^14^C]glucose-1-phosphate (295 mCi/mmol, PerkinElmer Life Sciences, Boston, MA, USA). The assay was performed in a final mixture of 50 mM Tris-HCl pH 8.5, 1 mM MgCl_2_, 0.1 mM ZnC_4_H_6_O_4_, 5 µM [^14^C]glucose-1-phosphate (3.5 × 10^4^ cpm) and 0–10 mM of water soluble compounds. After incubation at 37 °C for 30 min, the mixture was kept on ice and 10 mM sodium orthovanadate was added to stop the reaction. The solution was then applied to combined Dowex 50WX4 (H^+^ form) and Dowex 1 × 8 (formate) columns, and the elution and water wash fractions containing [^14^C]glucose were dried and quantified by scintillation counting.

## 4. Conclusions

New non hydrolysable glycosyl diphosphate mimics displaying an original β-1*C*-(phosphino)-phosphonate structure were synthetized according to a general convergent strategy. The key steps of the synthesis involved the addition of conveniently protected δ-hexonolactones derived from Man, Glc and Glc*N*Ac onto the dianion of diethyl ethoxy(methyl)phosphinylmethyl phosphonate, followed by dehydration and subsequent reduction of the resulting double bond leading to pure β stereochemistry. The synthetized compounds were tested for their ability to inhibit DLO diphosphatase and phosphatase activities. Whereas the compounds had no effect on the Co^2+^-activated DLO diphosphatase at a concentration of 1 mM, complex concentration-dependent activatory and inhibitory effects on alkaline phosphatase were noted. These compounds are undergoing further exploration as potential modifiers of the enzymatic metabolism of phospho-compounds.

## Supplementary Materials

The following are available online. Experimental procedures for compounds **11**–**13**; NMR spectra for compounds **11**–**29**.
