# Supplementary material for: Synthetic Route to Glycosyl β-1C-(phosphino)-phosphonates as Unprecedented Stable Glycosyl Diphosphate Analogs and Their Preliminary Biological Evaluation
Source: Molecules. 2020 Oct 27;25(21):4969. doi: 10.3390/molecules25214969 (PMC7663146; doi:10.3390/molecules25214969)

Supplementary Materials

Synthetic Route to Glycosyl β-1C-(phosphino)-phosphonates as Unprecedented Stable Glycosyl Diphosphate Analogs and Their Preliminary Biological Evaluation

Michaël Bosco [1], Su-Jin Paik [2], Patricia Busca [1], Stuart E. H. Moore [2] and Christine Gravier-Pelletier [1,*]

[1] Université de Paris, Faculté des Sciences, Campus Saint-Germain-des-Prés, UMR CNRS 8601, LCBPT, 45 rue des Saints Pères, F-75006 Paris, France; michael.bosco@u-paris.fr (M.B.); patricia.busca@u-paris.fr (P.B.)

[2] Université de Paris, Faculté de Médecine Xavier Bichat, INSERM U1149, CRI, 16 rue Henri Huchard, F-75018 Paris, France; su-jin.paik@inserm.fr (S.-J.P.), stuart.moore@inserm.fr (S.E.H.M.)

* Correspondence: christine.gravier-pelletier@u-paris.fr; Tel.: +33-176-534-228

Academic Editor: László Somsák

Received: 2 October 2020; Accepted: 26 October 2020; Published: 26 October 2020

Experimental procedures for compounds **11**, **12**, **13** S2

NMR Spectra of compound **11** S4

NMR Spectra of compound **12** S5

NMR Spectra of compound **13** S6

NMR Spectra of compound **14** S7

NMR Spectra of compound **15** S8

NMR Spectra of compound **16** S10

NMR Spectra of compound **17** S11

NMR Spectra of compound **18** S13

NMR Spectra of compound **19** S14

NMR Spectra of compound **20** S16

NMR Spectra of compound **21** S17

NMR Spectra of compound **22** S18

NMR Spectra of compound **23** S20

NMR Spectra of compound **24** S21

NMR Spectra of compound **25** S23

NMR Spectra of compound **26** S25

NMR Spectra of compound **27** S27

NMR Spectra of compound **28** S29

NMR Spectra of compound **29** S31

Preparation of lactones **11**, **12**, **13** was realized by oxidation of lactols by iodine inspired by the methodology developed by Fusaro *et al* [1].

2,3,4,5-tetra-*O*-benzyl-D-*mannono*-1,5-lactone 11

To a solution of 2,3,4,5-tetra-*O*-benzyl-D-*manno*-pyrannose **6** (5.67 g, 10.5 mmol) in anhydrous dichloromethane (106 mL) were added potassium carbonate (4.35 g, 31.5 mmol) and iodine (8 g, 31.5 mmol). After stirring for 14 h, the reaction was stopped by addition of 100 mL of an aqueous solution of sodium thiosulfate (10% in weight). The solution was extracted with dichloromethane (3 x 100 mL). The combined organic layers were washed with brine (400 mL), dried over sodium sulfate anhydrous, filtered and concentrated under reduced pressure. Purification of the residue by column chromatography (silica gel 300 mL, EtOAc/cyclohexane, 7/3, v/v) afforded the product **11** (4.15 g, 7.7 mmol, 73%) as a white solid.

NMR spectra were in accordance with previously published data [2].

^{1}H NMR (500 MHz, $CDCl_3$) δ 7.44 – 7.27 (m, 18H, H aromatic), 7.14 – 7.09 (m, 2H, H aromatic), 5.07 (d, *J* = 12.0 Hz, 1H, OCH_2Ph), 4.85 (d, *J* = 12.2 Hz, 1H, OCH_2Ph), 4.65 (d, *J* = 12.2 Hz, 1H, OCH_2Ph), 4.61 (d, *J* = 12.0 Hz, 1H, OCH_2Ph), 4.56 (d, *J* = 12.0 Hz, 1H, OCH_2Ph), 4.53 (d, *J* = 12.0 Hz, 1H, OCH_2Ph), 4.37 (d, *J* = 2.8 Hz, 1H, H-2), 4.35 (d, *J* = 12.0 Hz, 1H, CH_2 benz), 4.27 (d, *J* = 12.0 Hz, 1H, CH_2 benz), 4.25 (dt, *J* = 7.1, 4.6 Hz, 1H, H-5), 4.06 (dd, *J* = 2.8, 1.6 Hz, 1H, H-3), 3.80 (dd, *J* = 7.1, 1.6 Hz, 1H, H-4), 3.65 (d, *J* = 4.6 Hz, 2H, H-6, H-6′);
^{13}C NMR (126 MHz, $CDCl_3$) δ 169.5 (C-1), 137.88, 137.86, 137.4, 137.0 (4C, Cq aromatic), 128.65, 128.63, 128.54, 128.53, 128.31, 128.30, 128.11, 128.08, 128.05, 128.01, 127.96, 127.9 (20C, CH aromatic), 78.8 (C-5), 76.9 (C-3), 76.3 (C-4), 75.7 (C-2), 73.7, 73.12, 73.10, 72.03 (4C, OCH_2Ph), 69.4 (C-6).

2,3,4,5-tetra-*O*-benzyl-D-*glucono*-1,5-lactone 12

To a solution of 2,3,4,5-tetra-*O*-benzyl-D-*gluco*-pyrannose **6** (7.22 g, 13.35 mmol) in anhydrous dichloromethane (135 mL) were added potassium carbonate (5.53 g, 40.1 mmol) and iodine (10.2 g, 40.2 mmol). After stirring for 16 h, the reaction was stopped by addition of 200 mL of an aqueous solution of sodium thiosulfate (10% in weight). The solution was extracted with dichloromethane (3 x 100 mL). The combined organic layers were washed with brine (400 mL), dried over sodium sulfate anhydrous, filtered and concentrated under reduced pressure. Purification of the residue by column chromatography (silica gel 400 mL, EtOAc/cyclohexane, 7/3, v/v) afforded the product **11** (4.82 g, 8.94 mmol, 67%) as a white solid.

NMR spectra were in accordance with previously published data [1].

^{1}H NMR (500 MHz, $CDCl_3$) δ 7.55 – 7.03 (m, 20H, H aromatic), 5.01 (d, *J* = 11.4 Hz, 1H, OCH_2Ph), 4.75 (d, *J* = 11.7 Hz, 1H, OCH_2Ph), 4.73 (d, *J* = 11.6 Hz, 1H, OCH_2Ph), 4.66 (d, *J* = 11.4 Hz, 1H, OCH_2Ph), 4.61 (d, *J* = 11.7 Hz, 1H, OCH_2Ph), 4.58 (d, *J* = 12.0 Hz, 1H, OCH_2Ph), 4.54 (d, *J* = 11.6 Hz, 1H, OCH_2Ph), 4.50

[1] M. B. Fusaro, V. Chagnault, S. Josse, D. Postel, Metal-free oxidative lactonization of carbohydrates using molecular iodine, Tetrahedron 69 (2013), 5880-5883.
[2] Xie J., Molina A., Czernecki S., Alkylidenation of sugar lactones and further transformation to C-glycosides, J. Carbohydr. Chem. 18 (1999), 481-498.

(d, *J* = 12.0 Hz, 1H, OC*H*$_2$Ph), 4.48 (ddd, *J* = 8.2, 3.3, 2.4 Hz, 1H), 4.14 (d, *J* = 6.5 Hz, 1H, H-2), 3.97 (dd, *J* = 8.2, 6.5 Hz, 1H, H-4), 3.93 (t, *J* = 6.5 Hz, 1H, H-3), 3.75 (dd, *J* = 11.0, 2.4 Hz, 1H, H-6), 3.69 (dd, *J* = 11.0, 3.3 Hz, 1H, H-6');
^{13}C NMR (126 MHz, $CDCl_3$) δ 169.4 (C-1), 137.8, 137.68, 137.66, 137.1 (4C, Cq aromatic), 128.61, 128.58, 128.5, 128.2, 128.14, 128.11, 128.10, 128.07, 128.0 (20C, CH aromatic), 81.1 (C-3), 78.3 (C-5), 77.6 (C-2), 76.2 (C-4), 74.1, 73.85, 73.84, 73.7 (4C, OCH$_2$Ph), 68.4 (C-6).

2-Acetamido-3,4,5-tetra-*O*-benzyl-2-deoxy-D-*glucono*-1,5-lactone 13

To a solution of 2-acetamido-3,4,5-tetra-*O*-benzyl-2-deoxy-D-*manno*-pyrannose **6** (1.925 g, 3.91 mmol) in anhydrous dichloromethane (120 mL) were added potassium carbonate (1.62 g, 11.8 mmol) and iodine (2.98 g, 11.8 mmol). After stirring for 16 h, the reaction was stopped by addition of 100 mL of an aqueous solution of sodium thiosulfate (10% in weight). The solution was extracted with dichloromethane (100 mL). The combined organic layers were brine dried over sodium sulfate anhydrous, filtered and concentrated under reduced pressure. Recrystallization from diethyl ether gave the desired product **13** (701 mg, 1.43 mmol, 36%) as a white solid.

NMR spectra were in accordance with previously published data [3].

^{1}H NMR (500 MHz, $CDCl_3$) *δ* 7.42 – 7.14 (m, 15H, H aromatic), 5.98 (d, *J* = 7.0 Hz, 1H, N*H*Ac), 4.82 (d, *J* = 11.8 Hz, 1H, OC*H*$_2$Ph), 4.81 (d, *J* = 11.1 Hz, 1H, OC*H*$_2$Ph), 4.67 (d, *J* = 11.1 Hz, 1H, OC*H*$_2$Ph), 4.62 (d, *J* = 12.1 Hz, 1H, OC*H*$_2$Ph), 4.59 (d, *J* = 12.1 Hz, 1H, OC*H*$_2$Ph), 4.50 (d, *J* = 11.8 Hz, 1H, OC*H*$_2$Ph), 4.47 – 4.42 (m, 1H, H-5), 4.07 – 3.96 (m, 3H, H-2, H-4, H-3), 3.79 (dd, *J* = 11.0, 2.8 Hz, 1H, H-6), 3.76 (dd, *J* = 11.0, 2.7 Hz, 1H, H-6'), 1.85 (s, 3H, COC*H*$_3$);
^{13}C NMR (126 MHz, $CDCl_3$) δ 170.5 (C-1), 168.7 (*C*OCH$_3$), 138.1, 137.72, 137.69 (3C, Cq aromatic), 128.72, 128.67, 128.6, 128.4, 128.22, 128.18, 128.1, 128.04, 128.02 (15C, CH aromatic), 79.8 (C-3), 78.8 (C-5), 76.3 (C-4), 74.7 (2C, OCH$_2$Ph), 73.8 (OCH$_2$Ph), 68.0 (C-6), 55.6 (C-2), 22.8 (COCH$_3$).

[3] Granier T., Vasella A., Synthesis and Some Transformations of 2-Acetamido-5-amino-3,4,6-tri-*O*-benzyl-2,5-dideoxy-D-glucono-1,5-lactam, Helv. Chim. Acta 81 (1998), 865-880.

^{1}H NMR, 500 MHz ($CDCl_3$), compound **11**

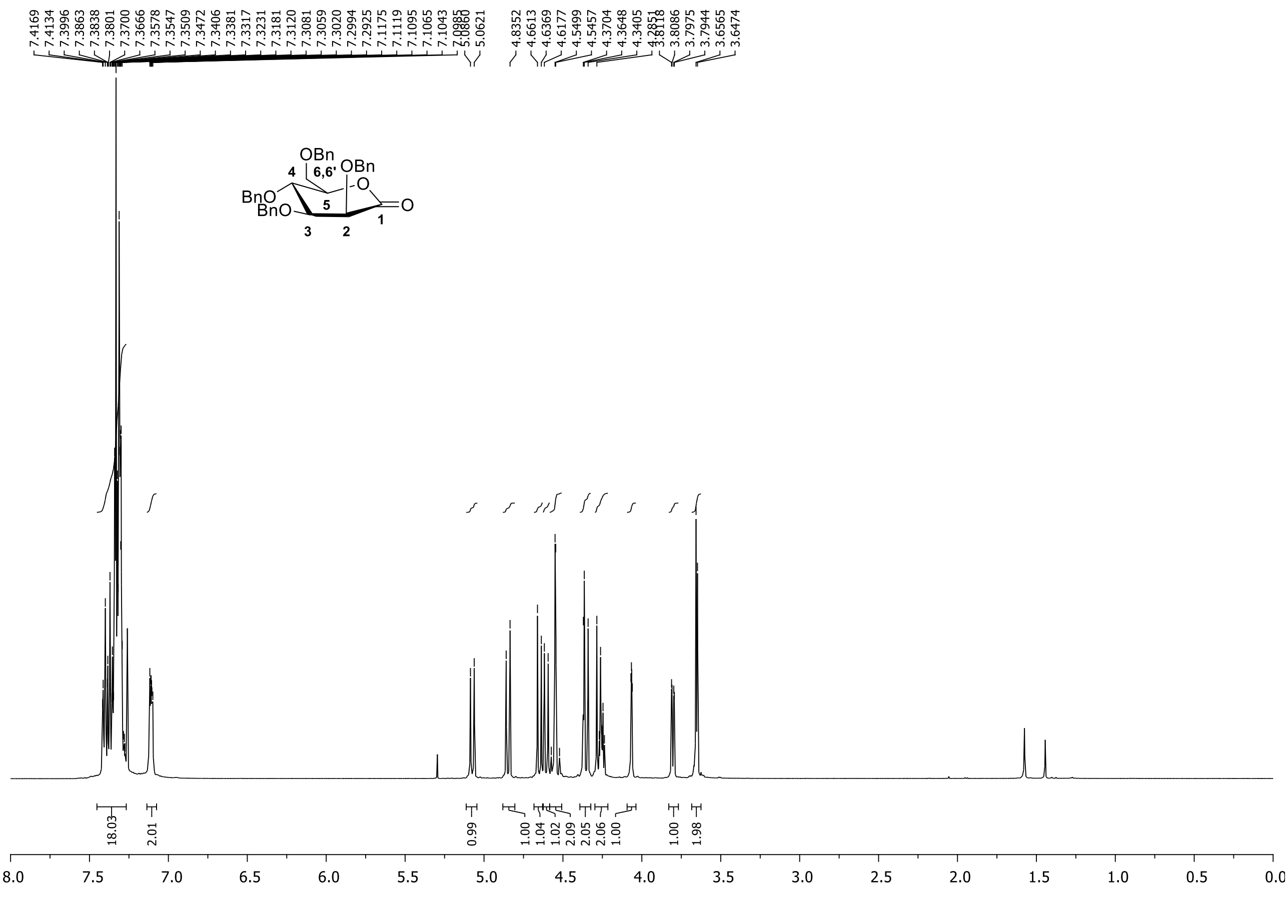

^{13}C NMR, 125 MHz ($CDCl_3$), compound **11**

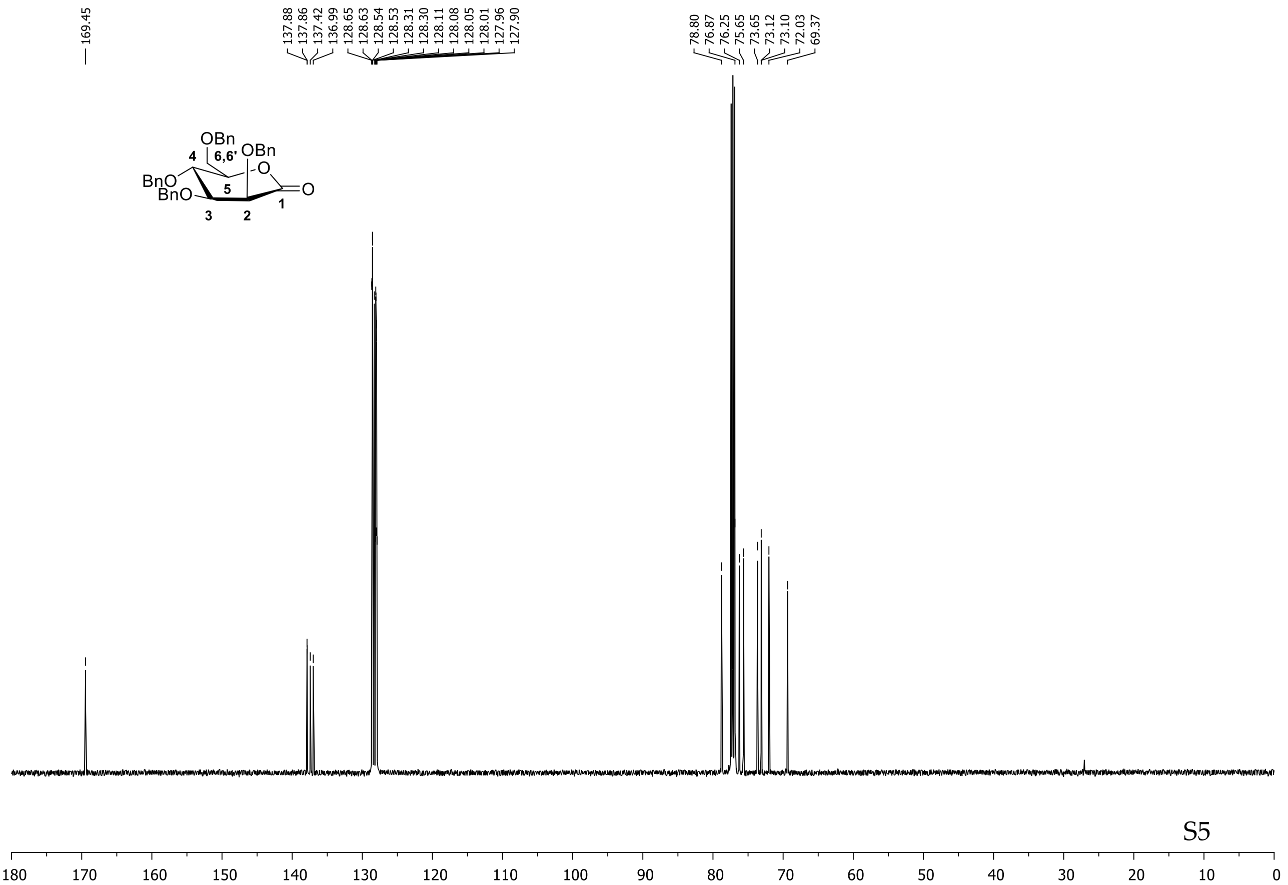

^{1}H NMR, 500 MHz ($CDCl_3$), compound **12**

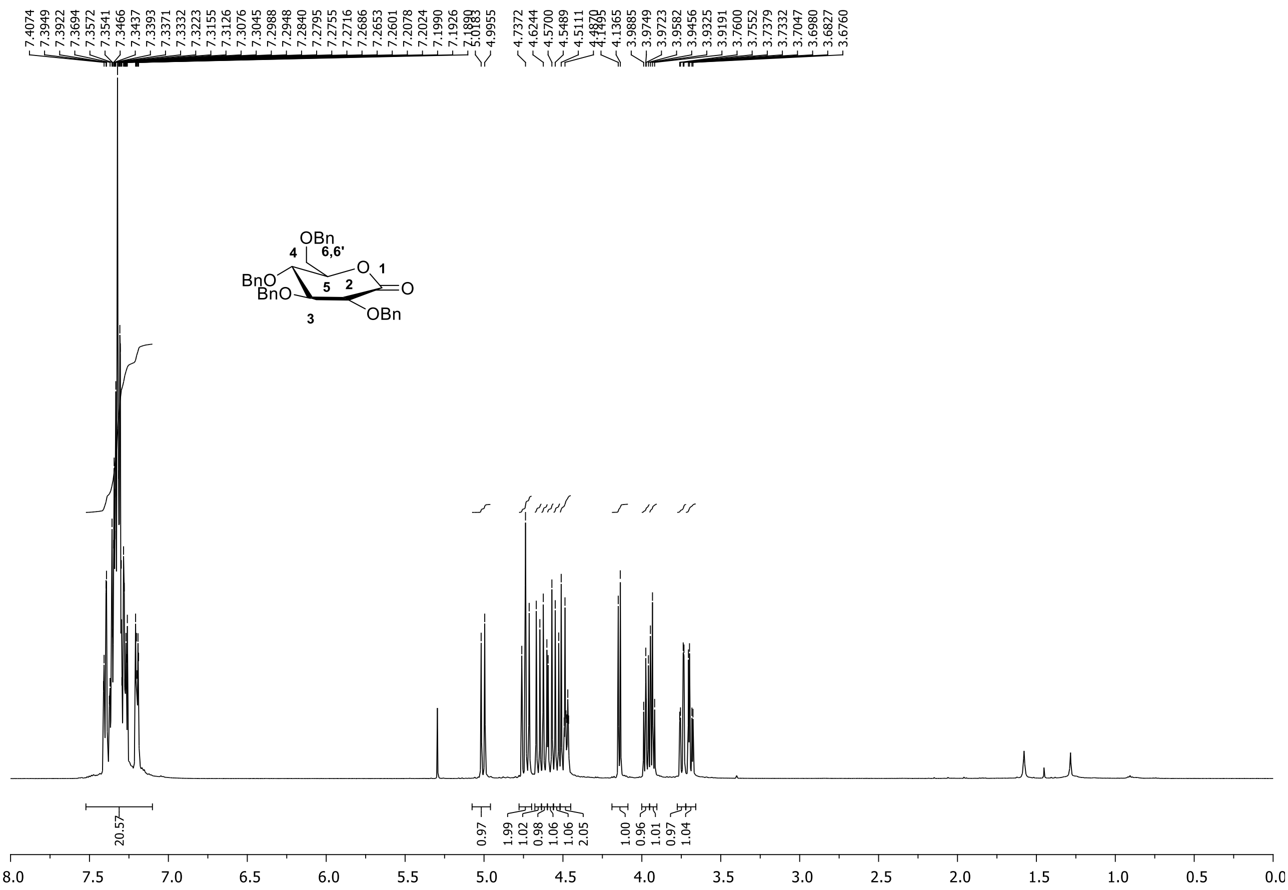

^{13}C NMR, 125 MHz ($CDCl_3$), compound **12**

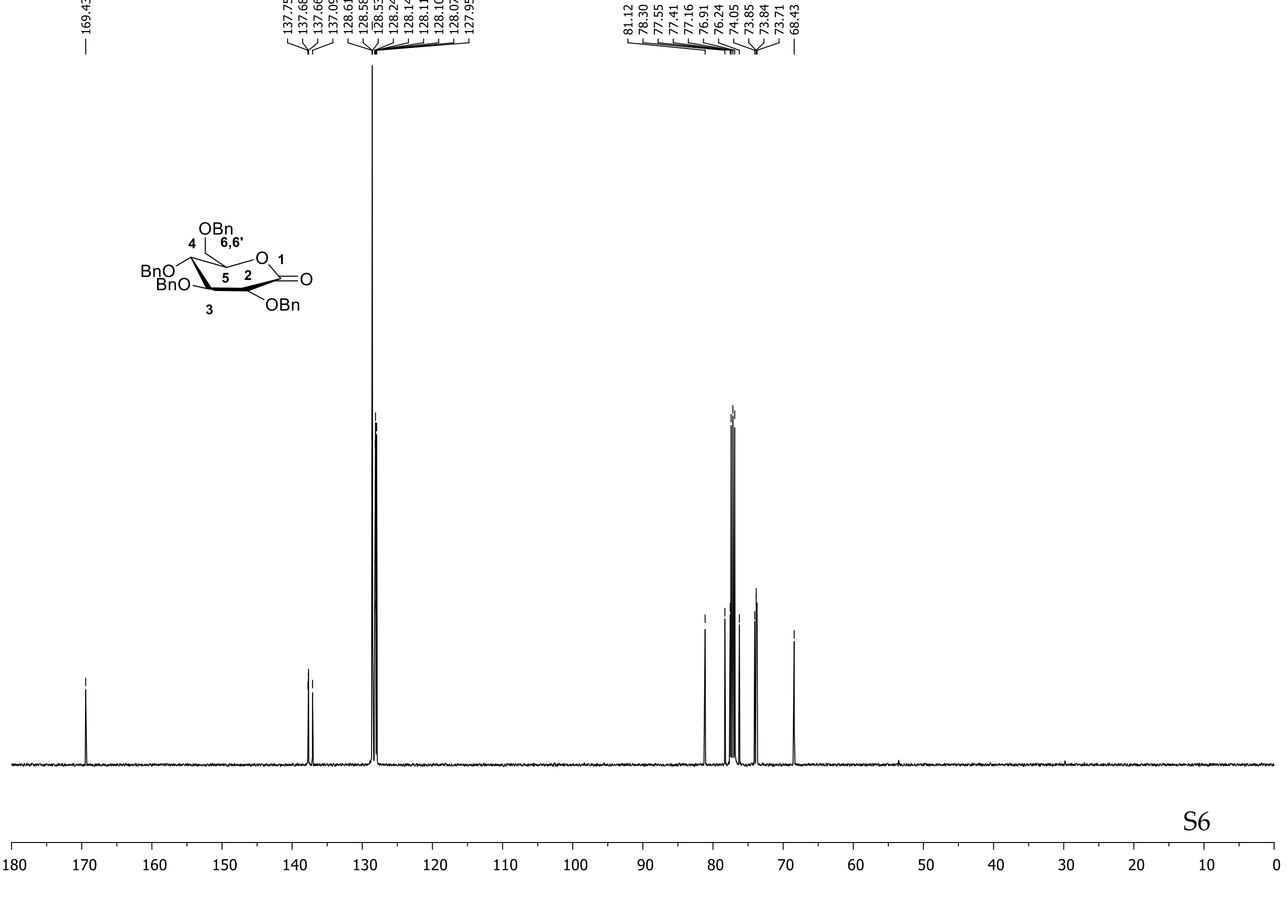

^{1}H NMR, 500 MHz ($CDCl_3$), compound **13**

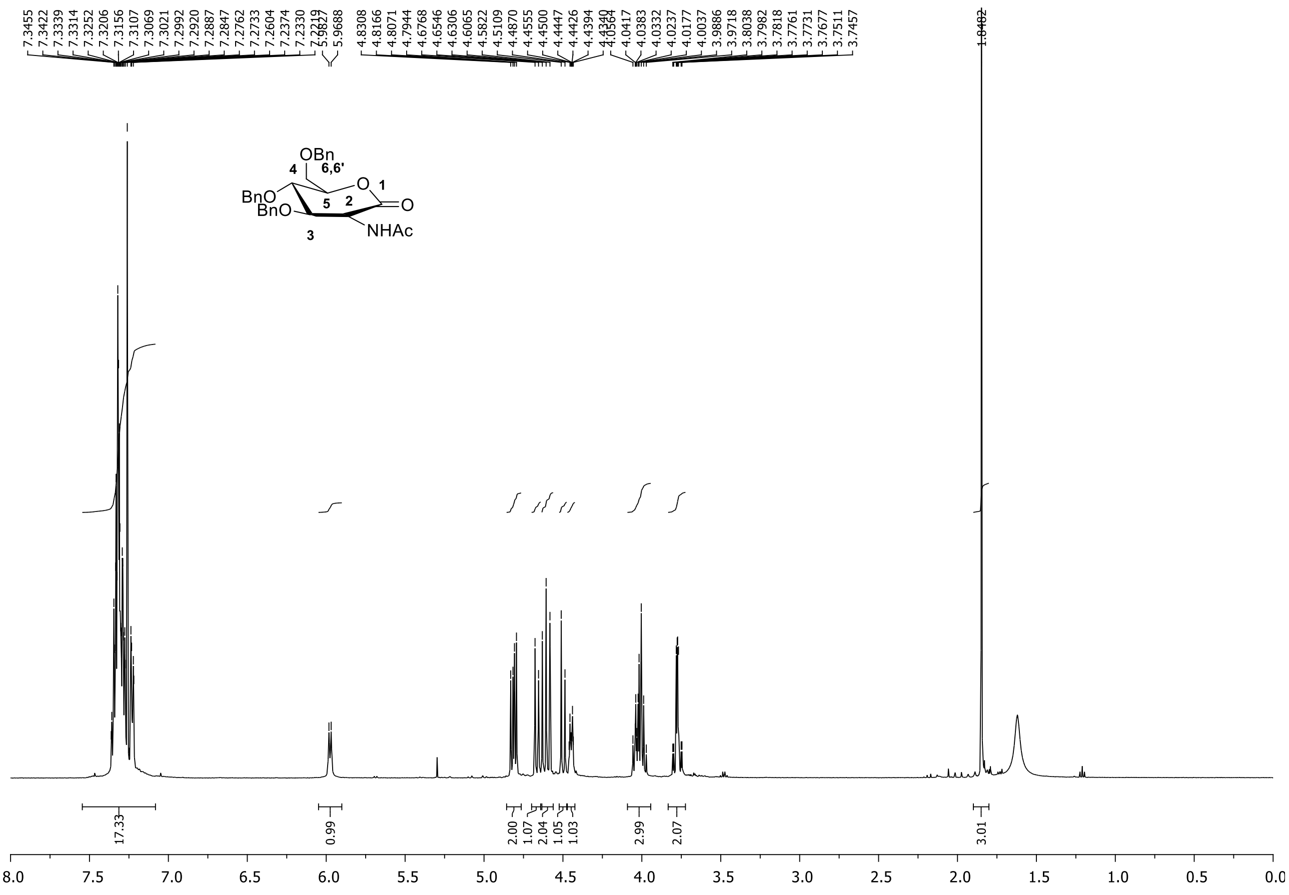

^{13}C NMR, 125 MHz ($CDCl_3$), compound **13**

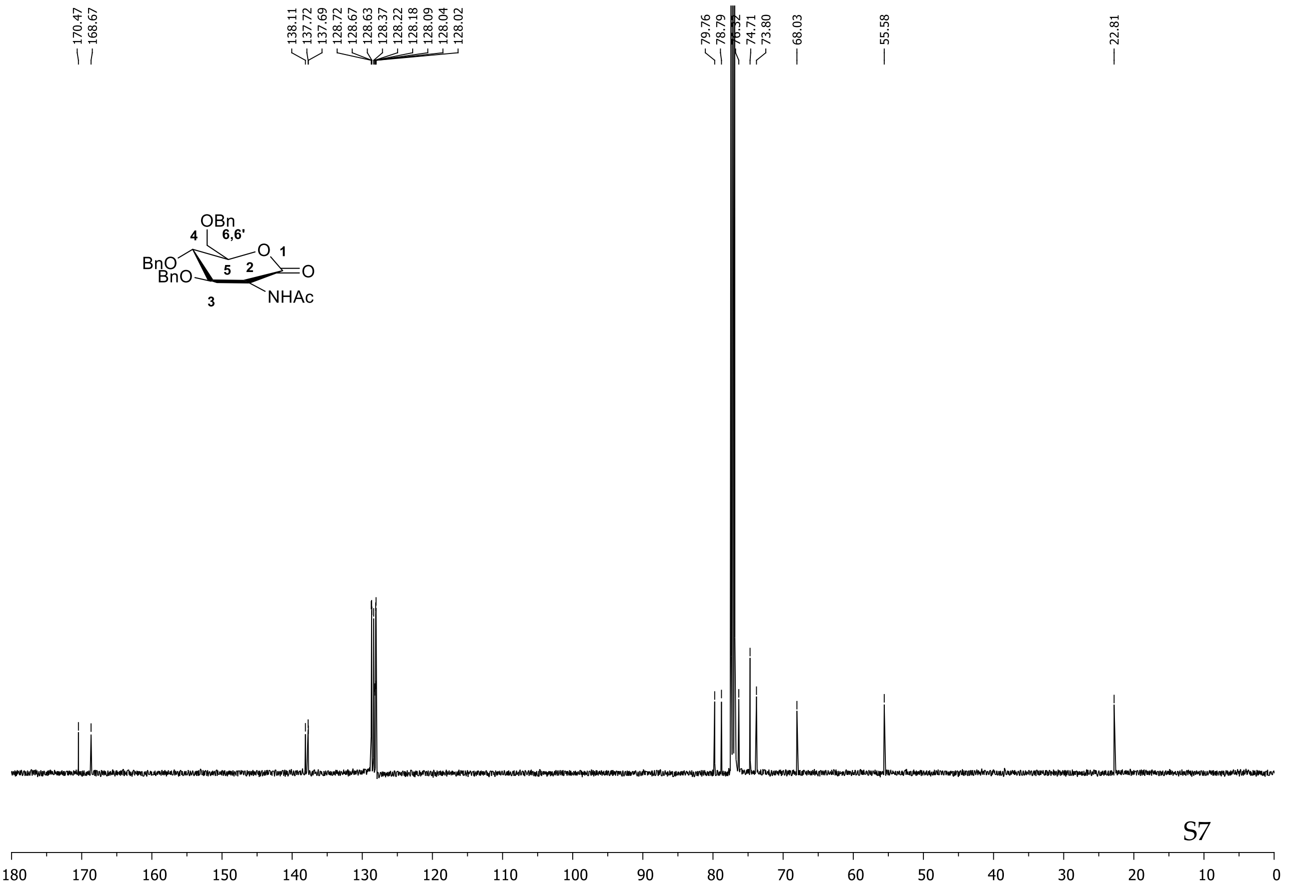

^{1}H NMR, 500 MHz ($CDCl_3$), compound **14**

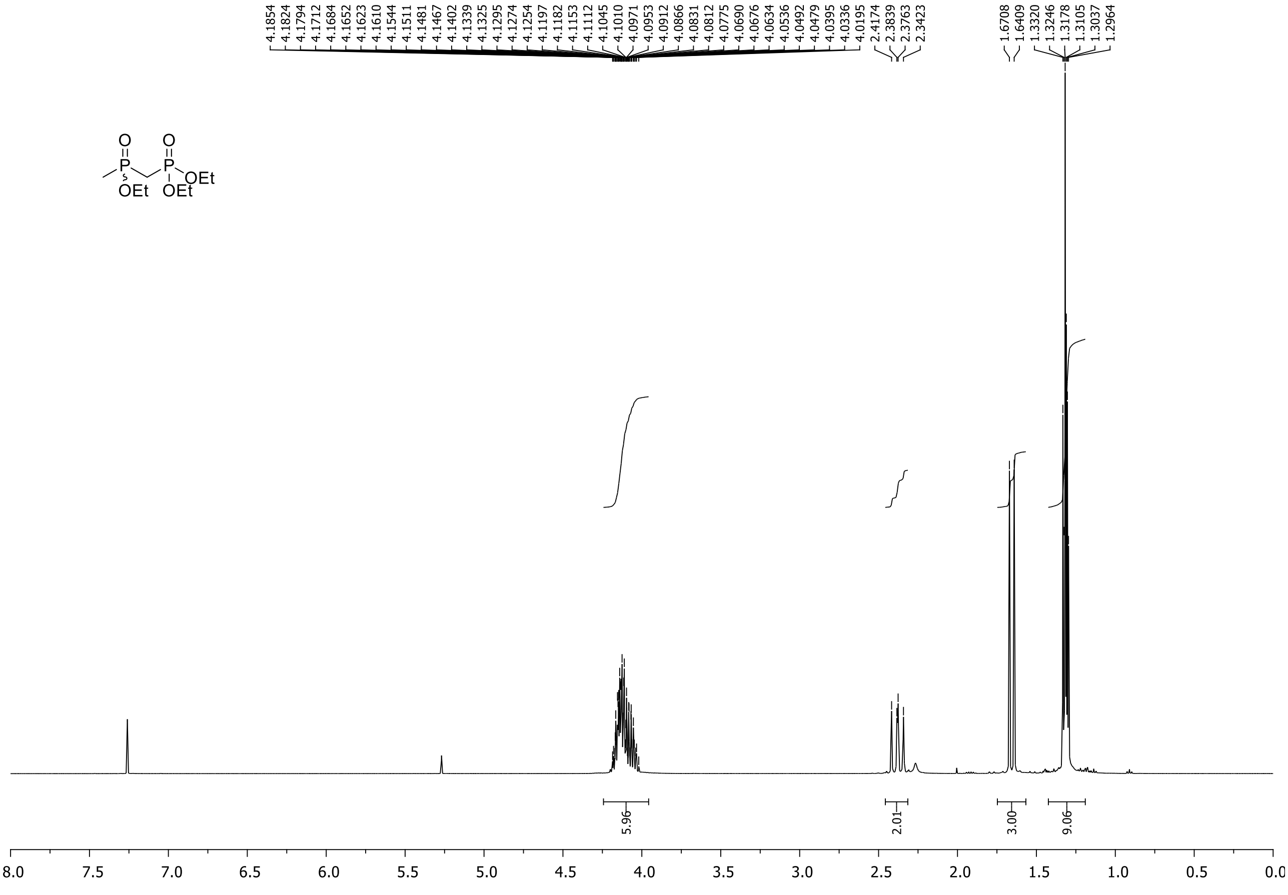

^{13}C NMR, 125 MHz ($CDCl_3$), compound **14**

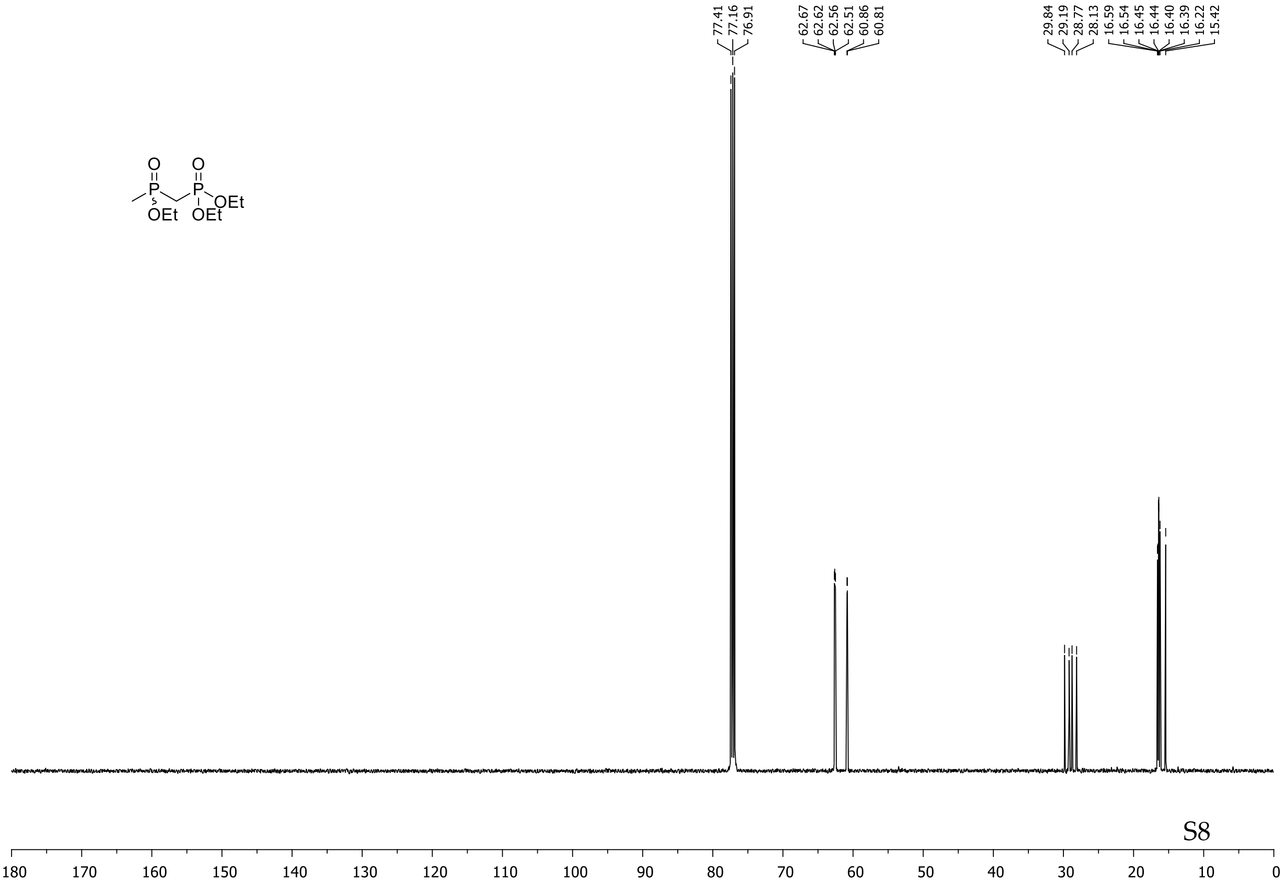

31P NMR, 202 MHz (CDCl3), compound **14**

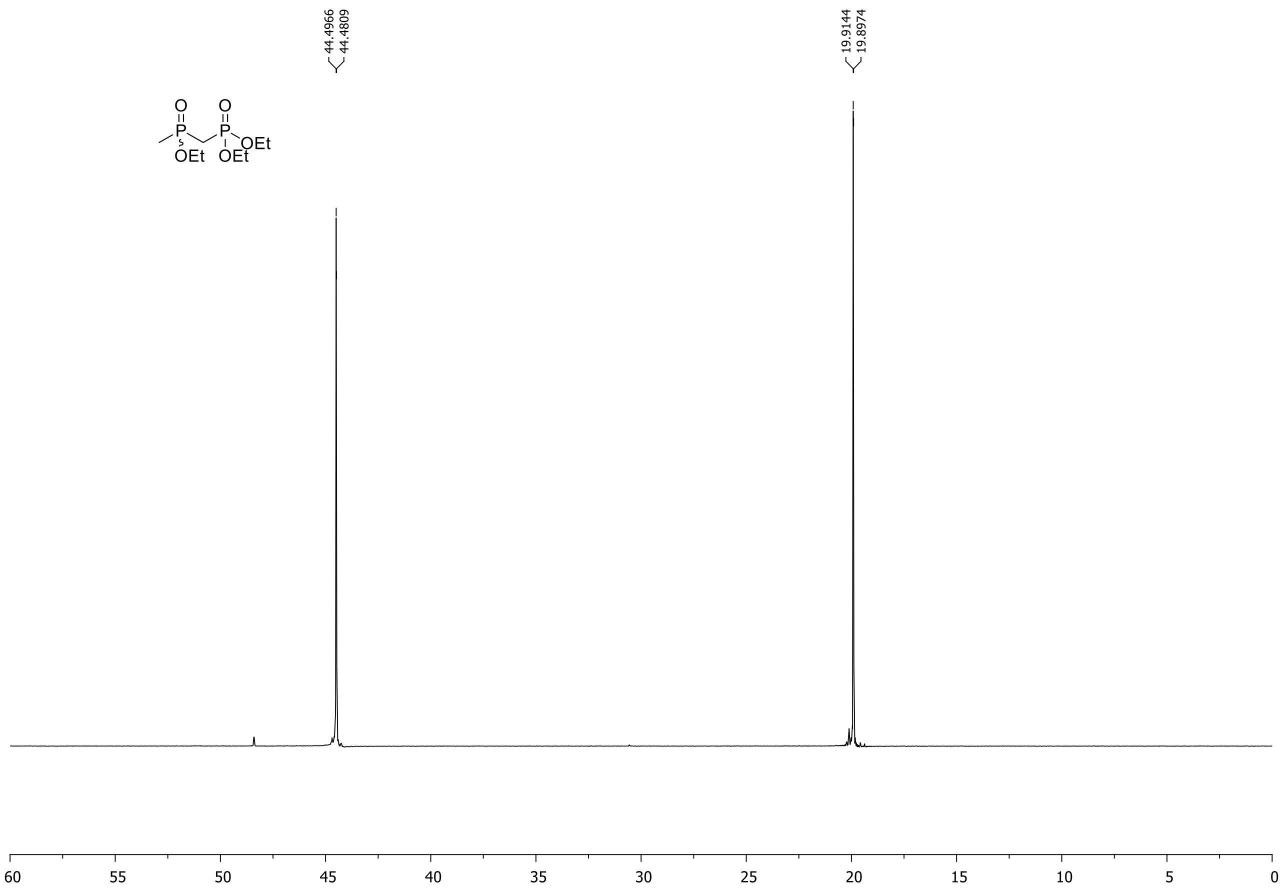

1H NMR, 500 MHz (CDCl3), compound **15**

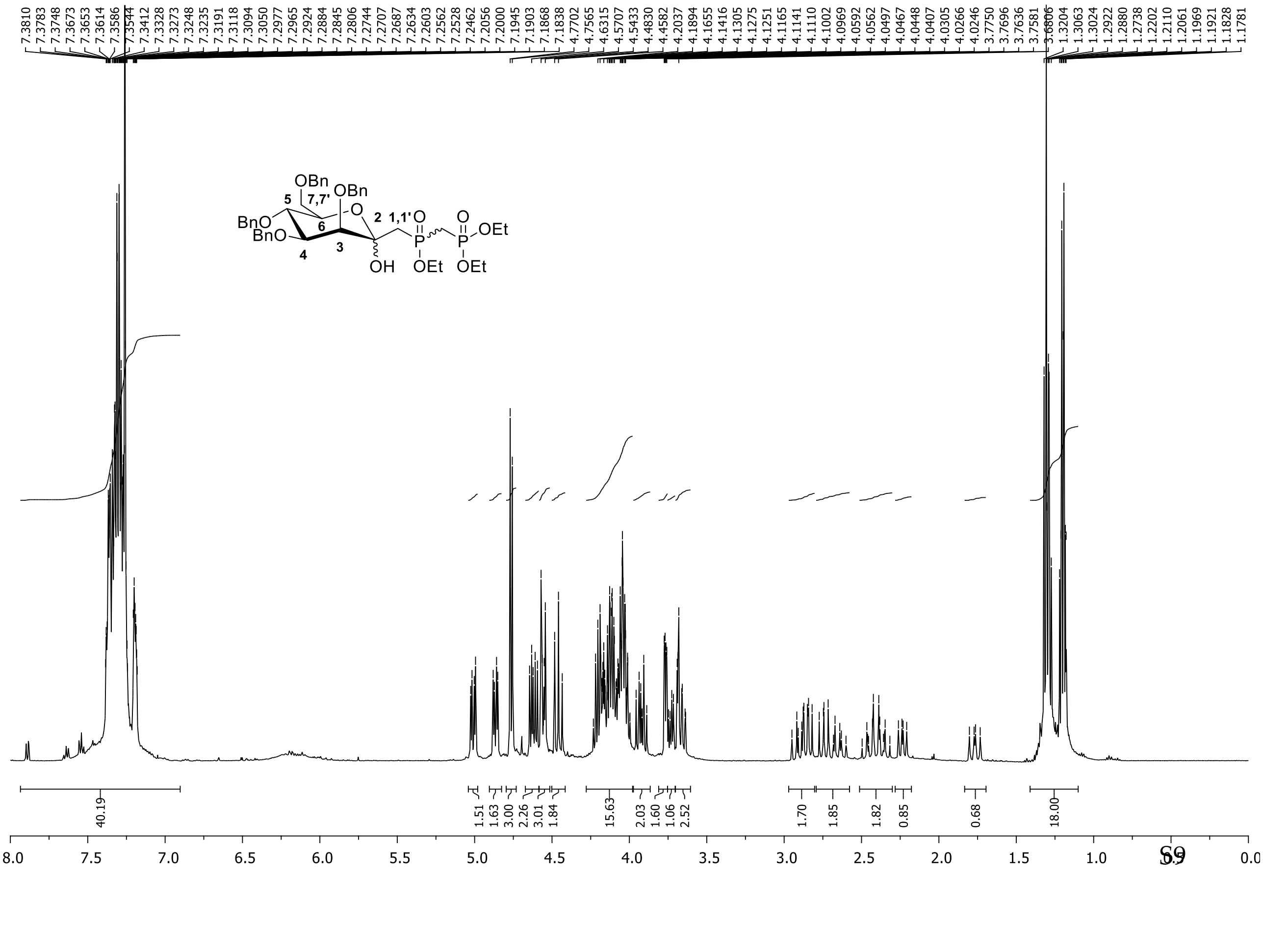

^{13}C NMR, 125 MHz ($CDCl_3$), compound **15**

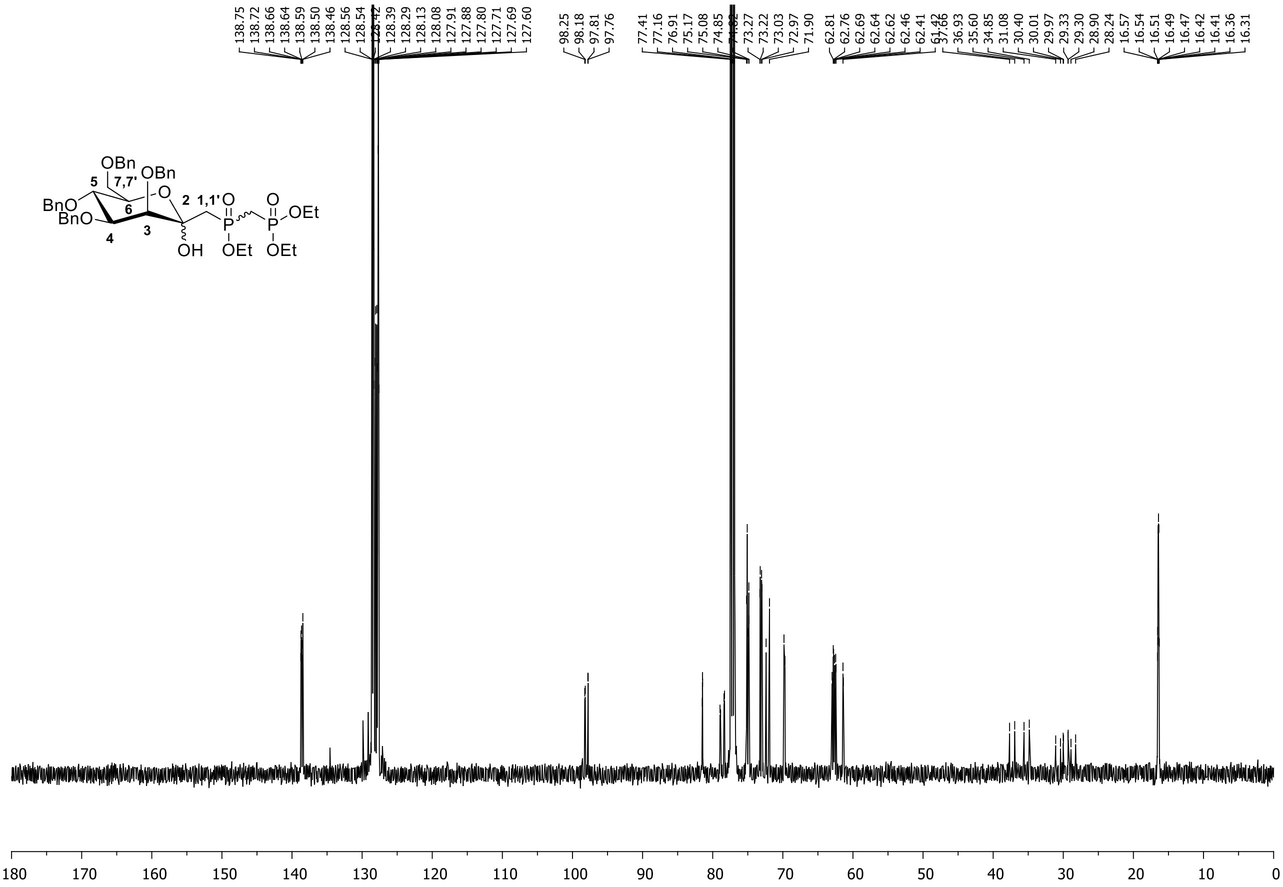

^{31}P NMR, 202 MHz ($CDCl_3$), compound **15**

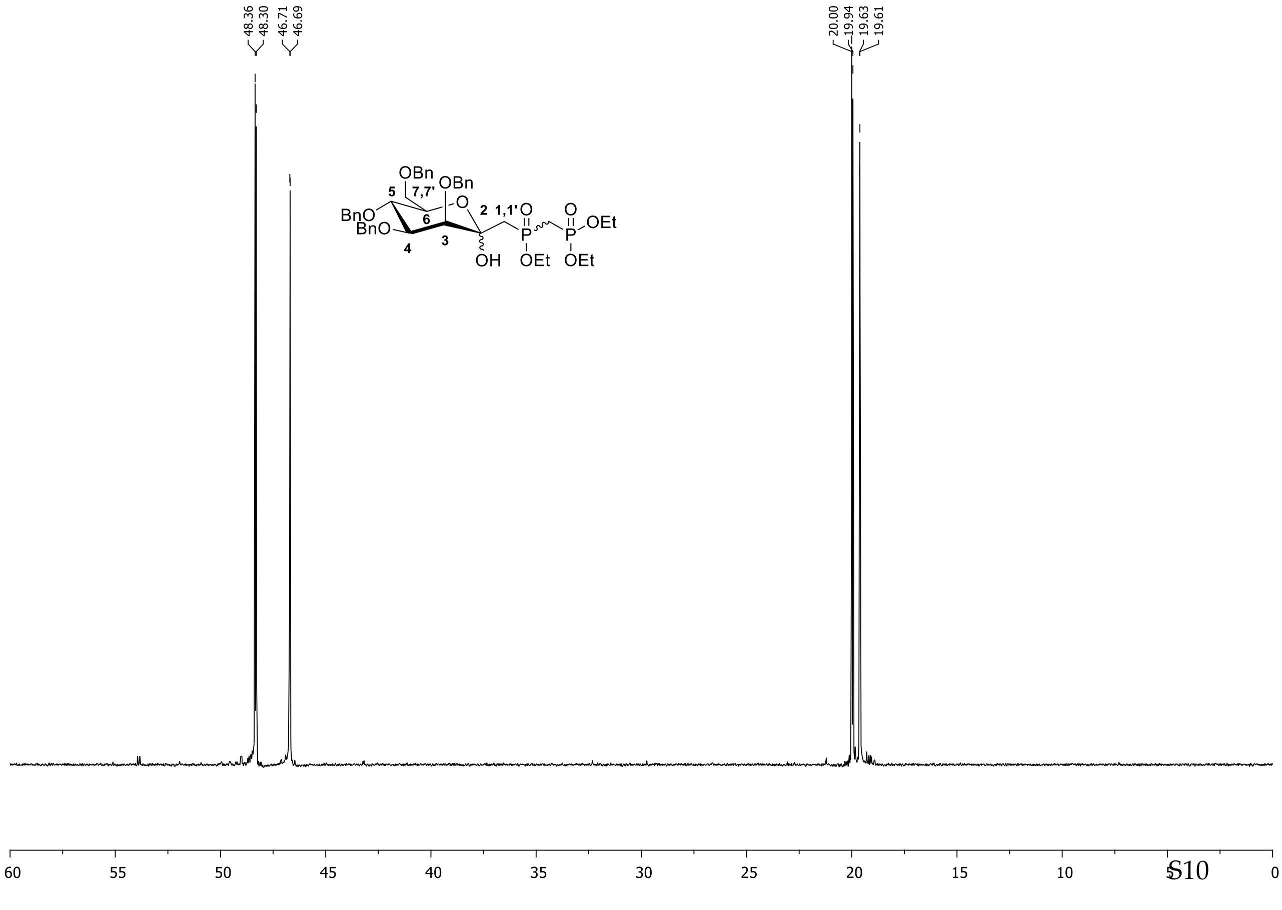

^{1}H NMR, 500 MHz ($CDCl_3$), compound **16**

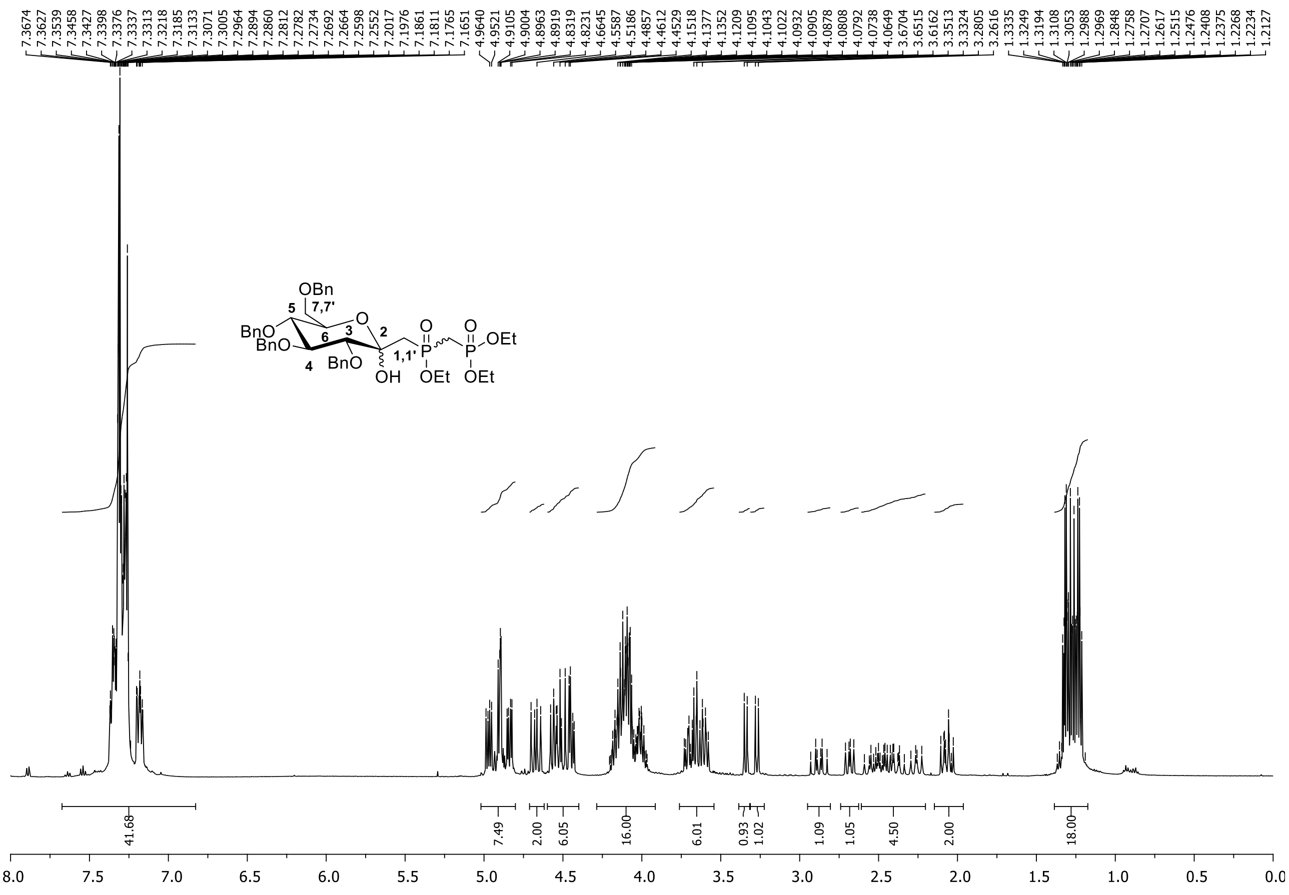

^{13}C NMR, 125 MHz ($CDCl_3$), compound **16**

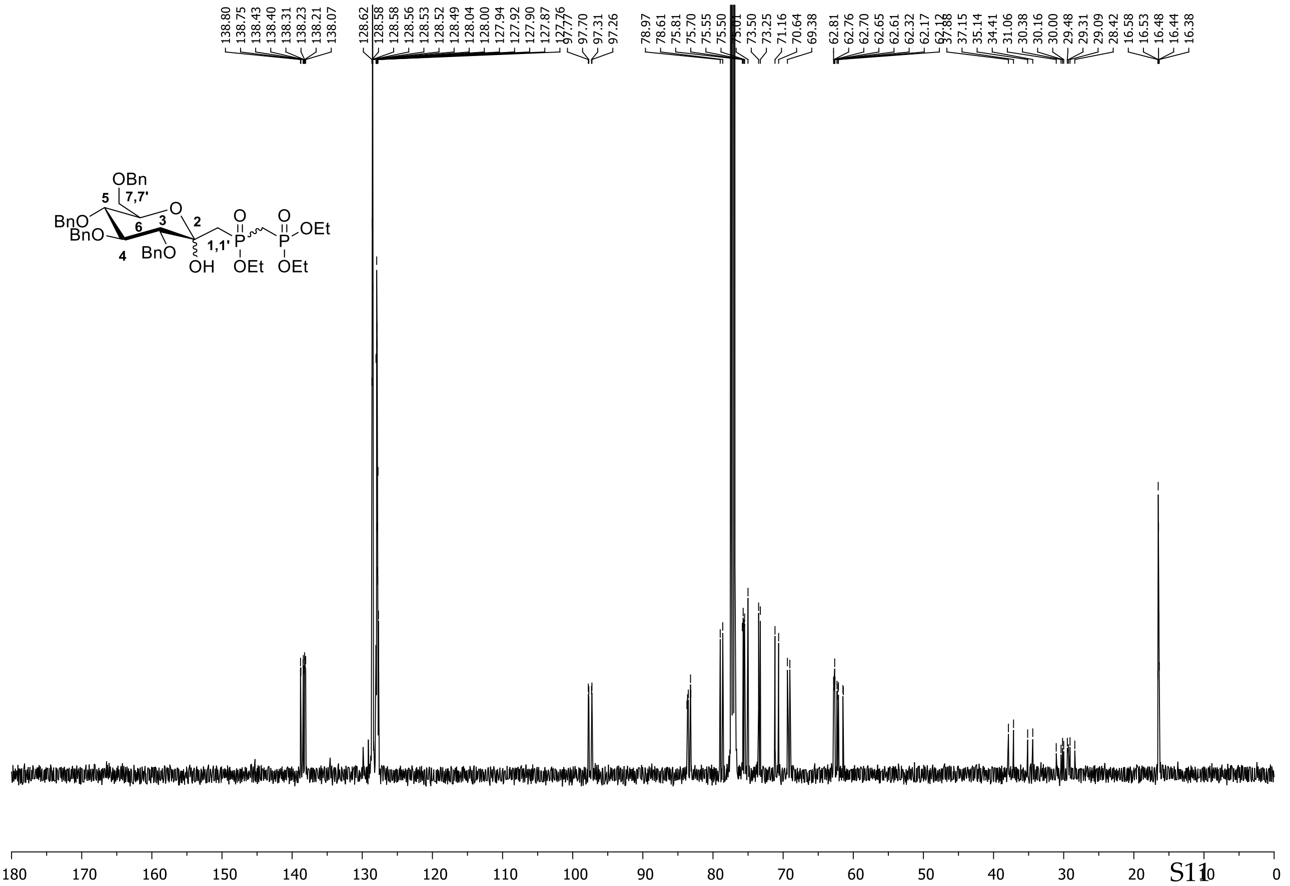

^{31}P NMR, 202 MHz ($CDCl_3$), compound **16**

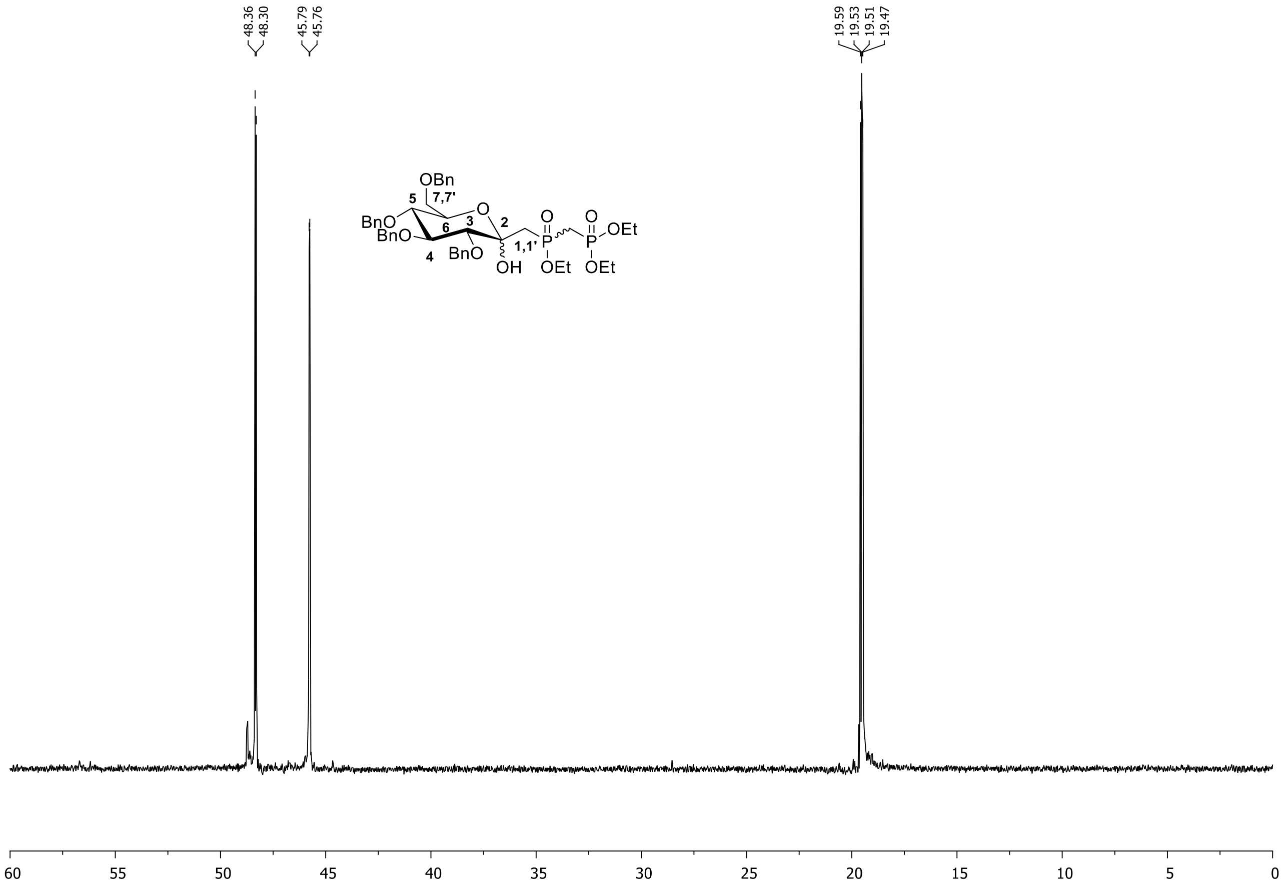

^{1}H NMR, 500 MHz ($CDCl_3$), compound **17**

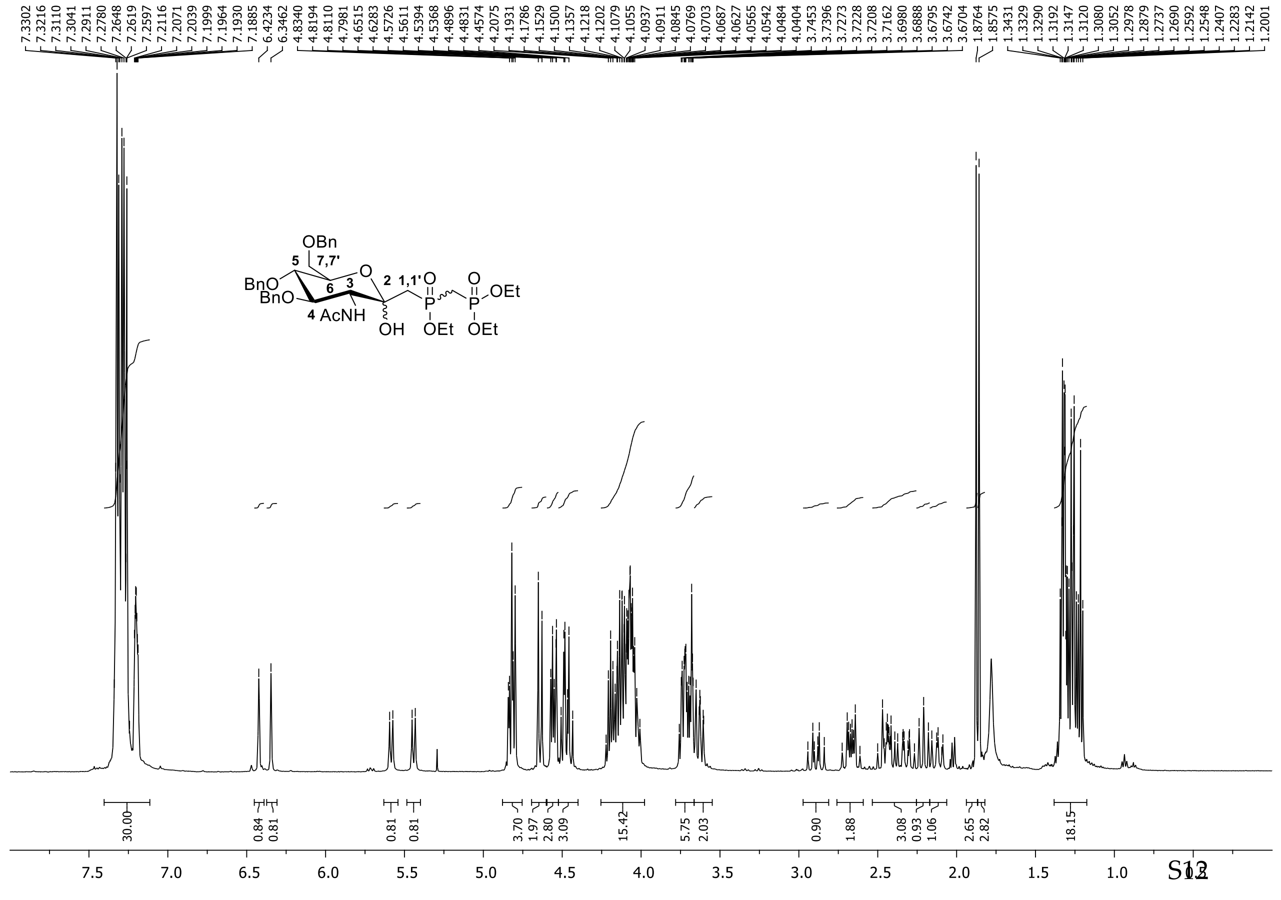

^{13}C NMR, 125 MHz ($CDCl_3$), compound **17**

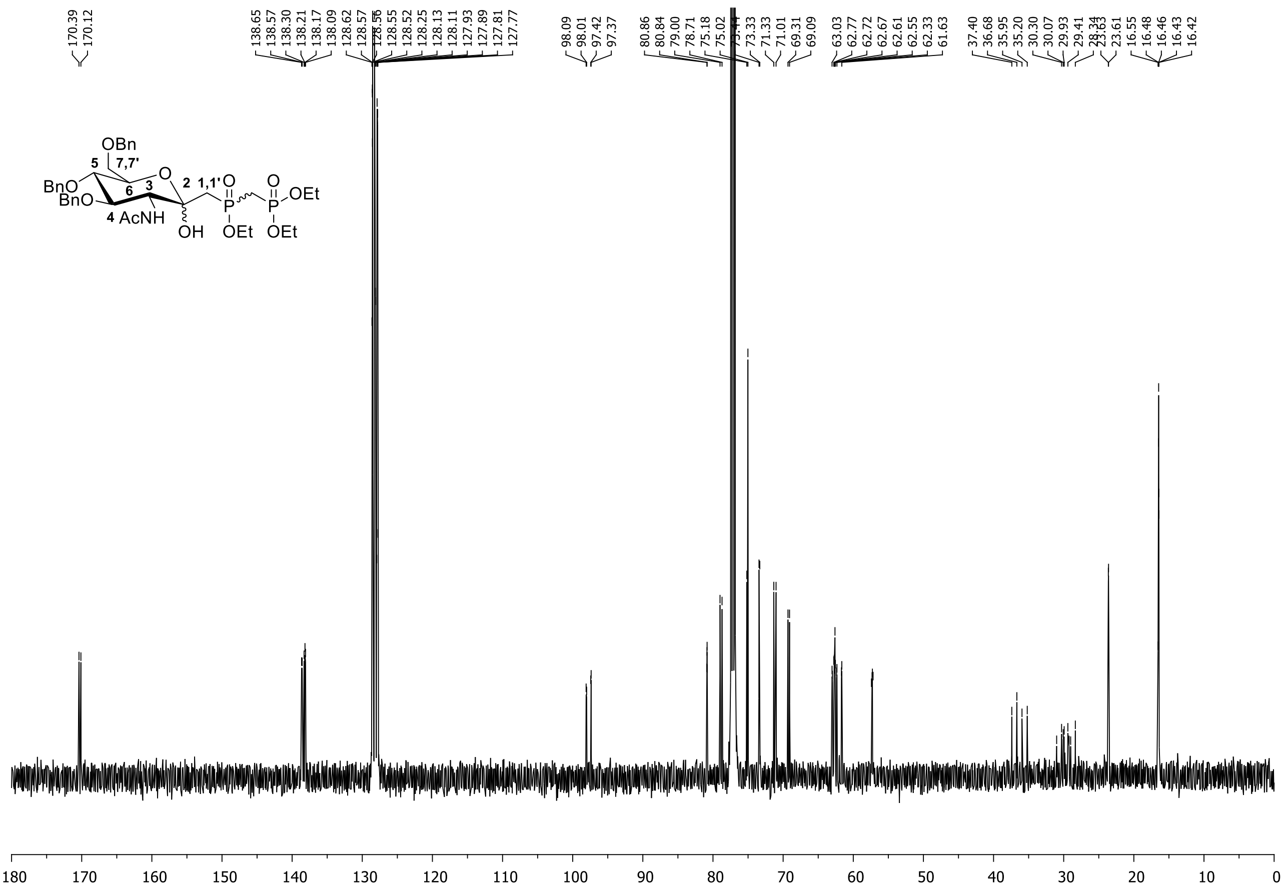

^{31}P NMR, 202 MHz ($CDCl_3$), compound **17**

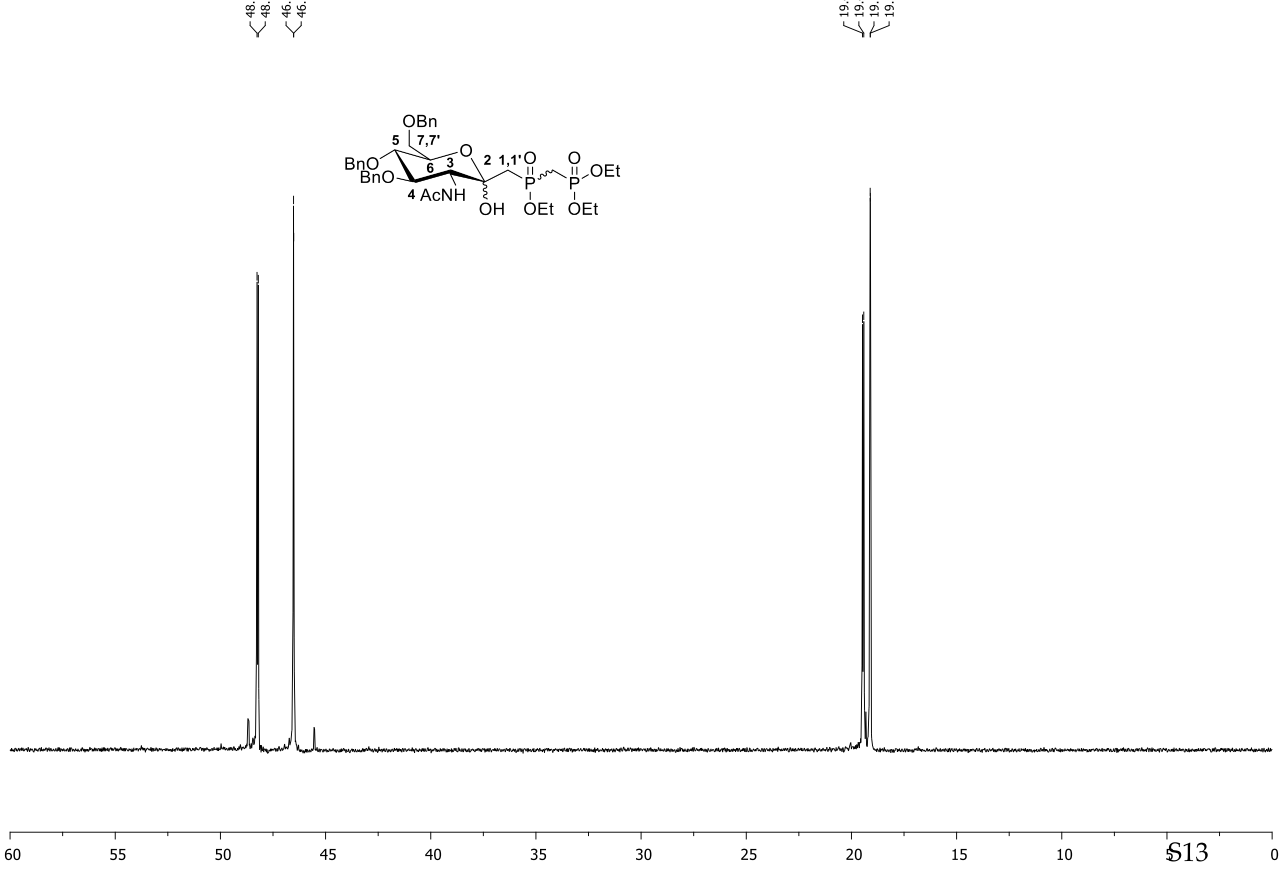

^{1}H NMR, 500 MHz ($CDCl_3$), compound **18**

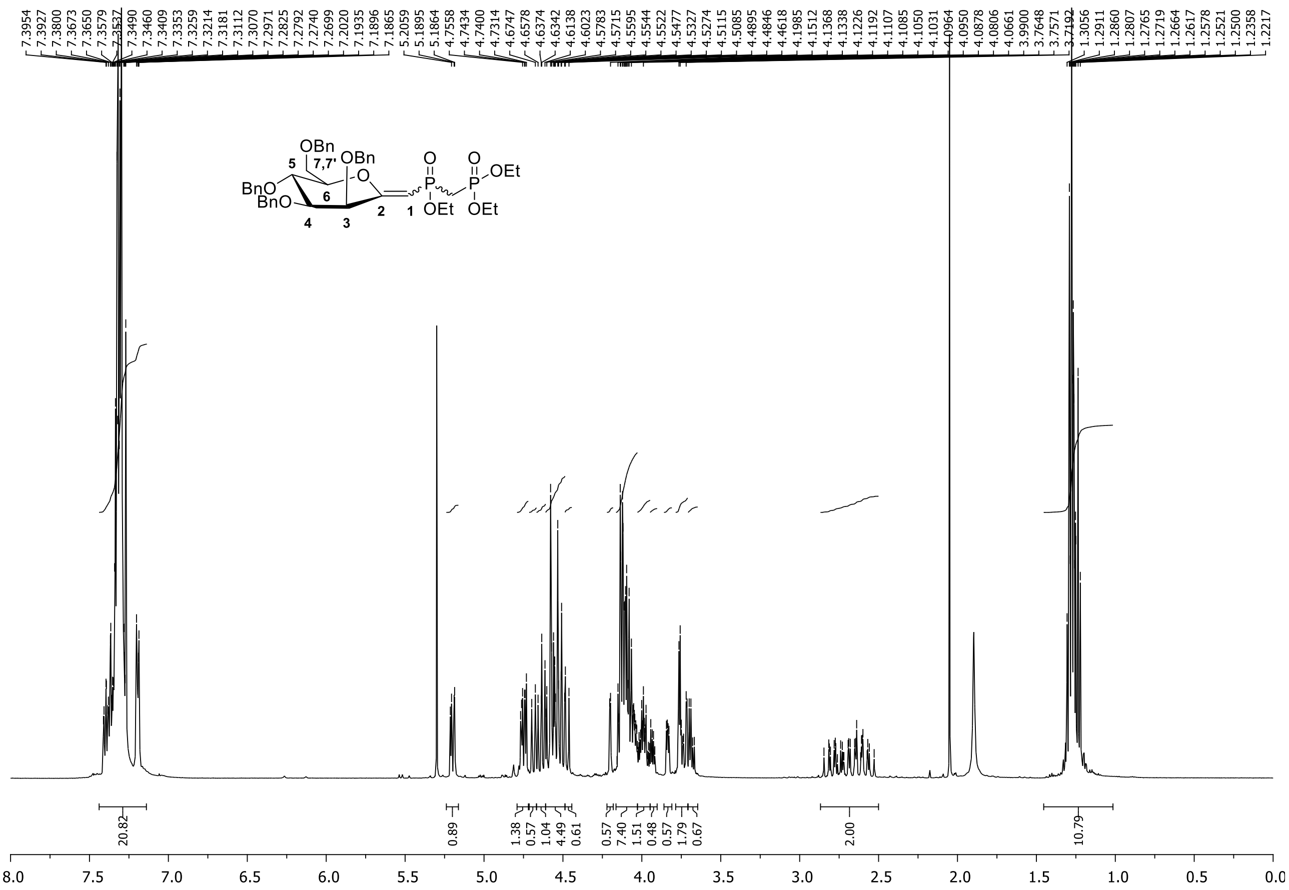

^{13}C NMR, 125 MHz ($CDCl_3$), compound **18**

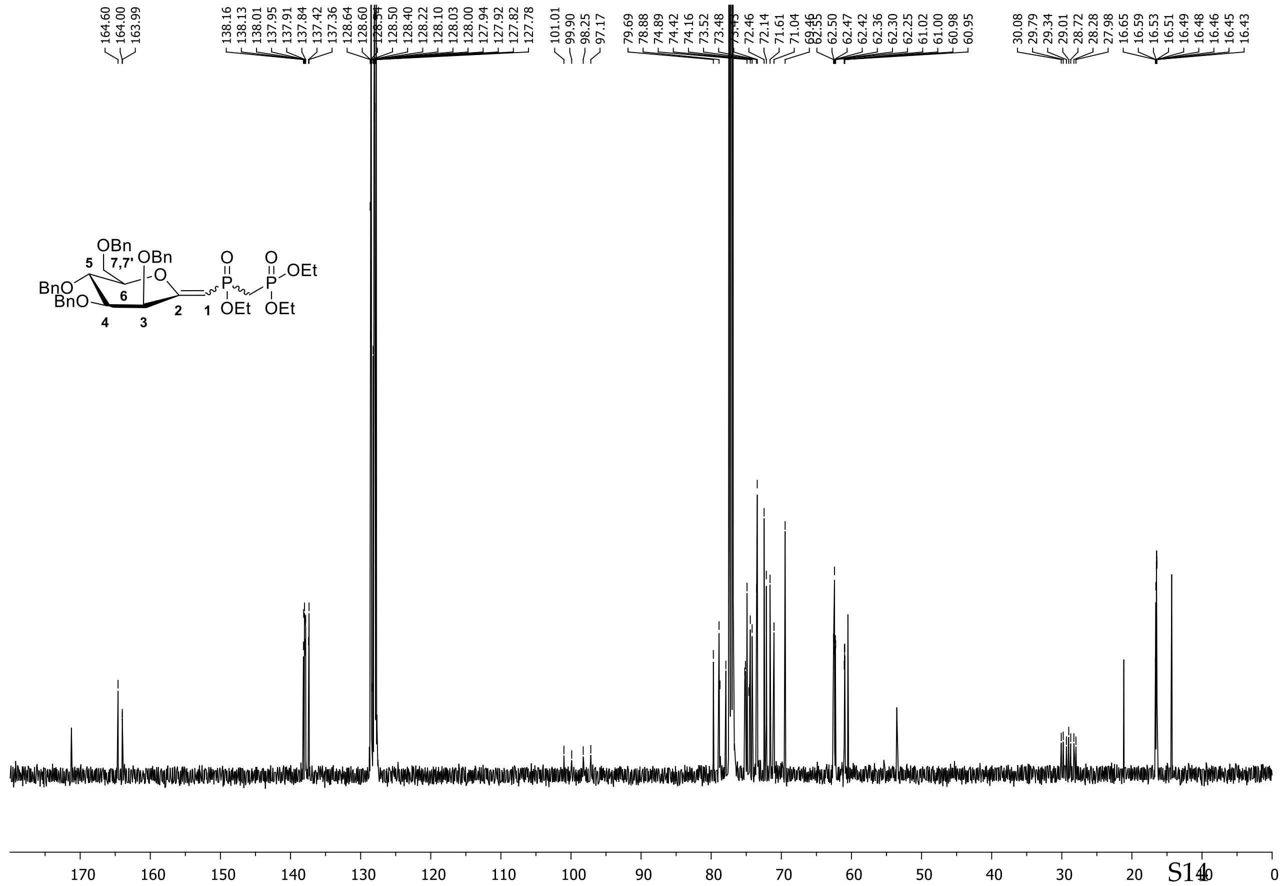

^{31}P NMR, 202 MHz ($CDCl_3$), compound **18**

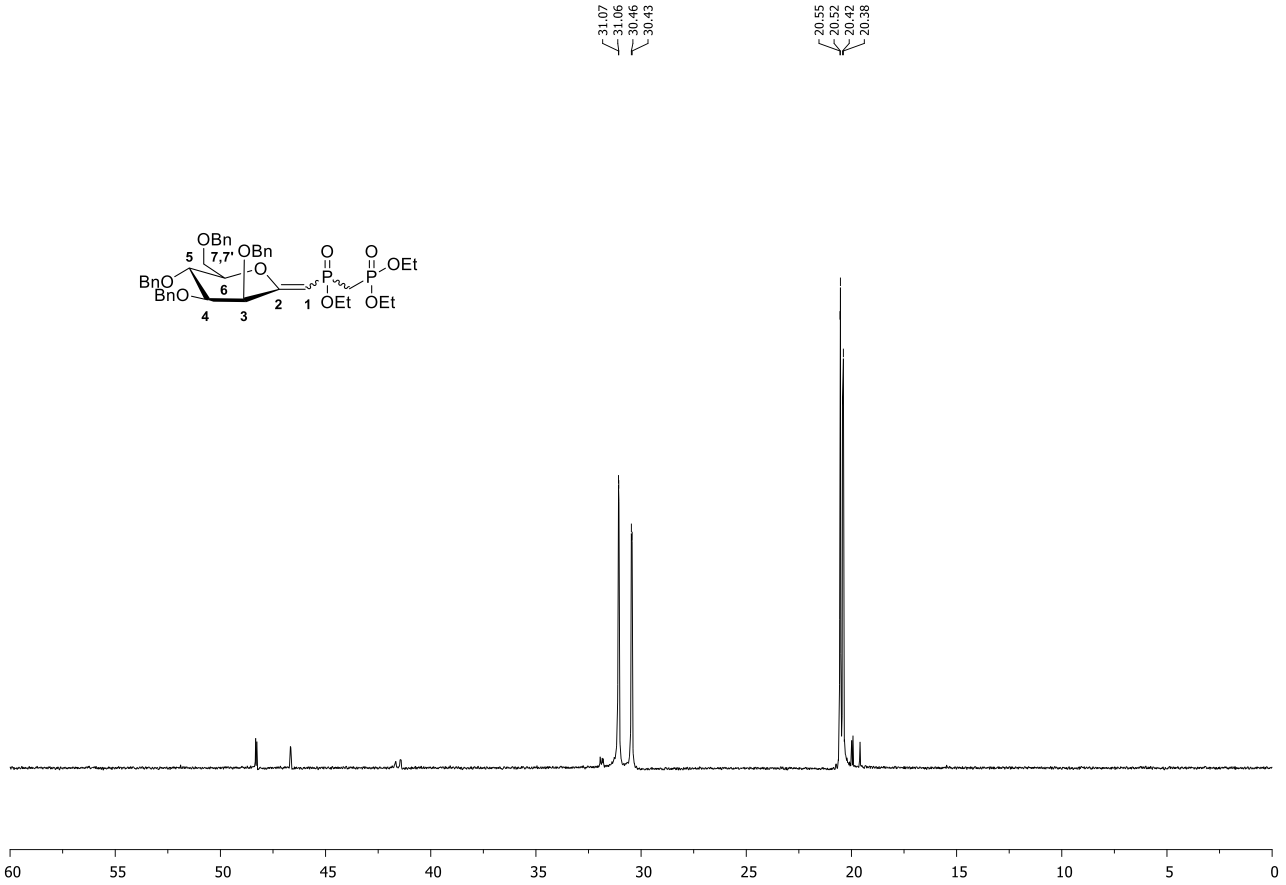

^{1}H NMR, 500 MHz ($CDCl_3$), compound **19**

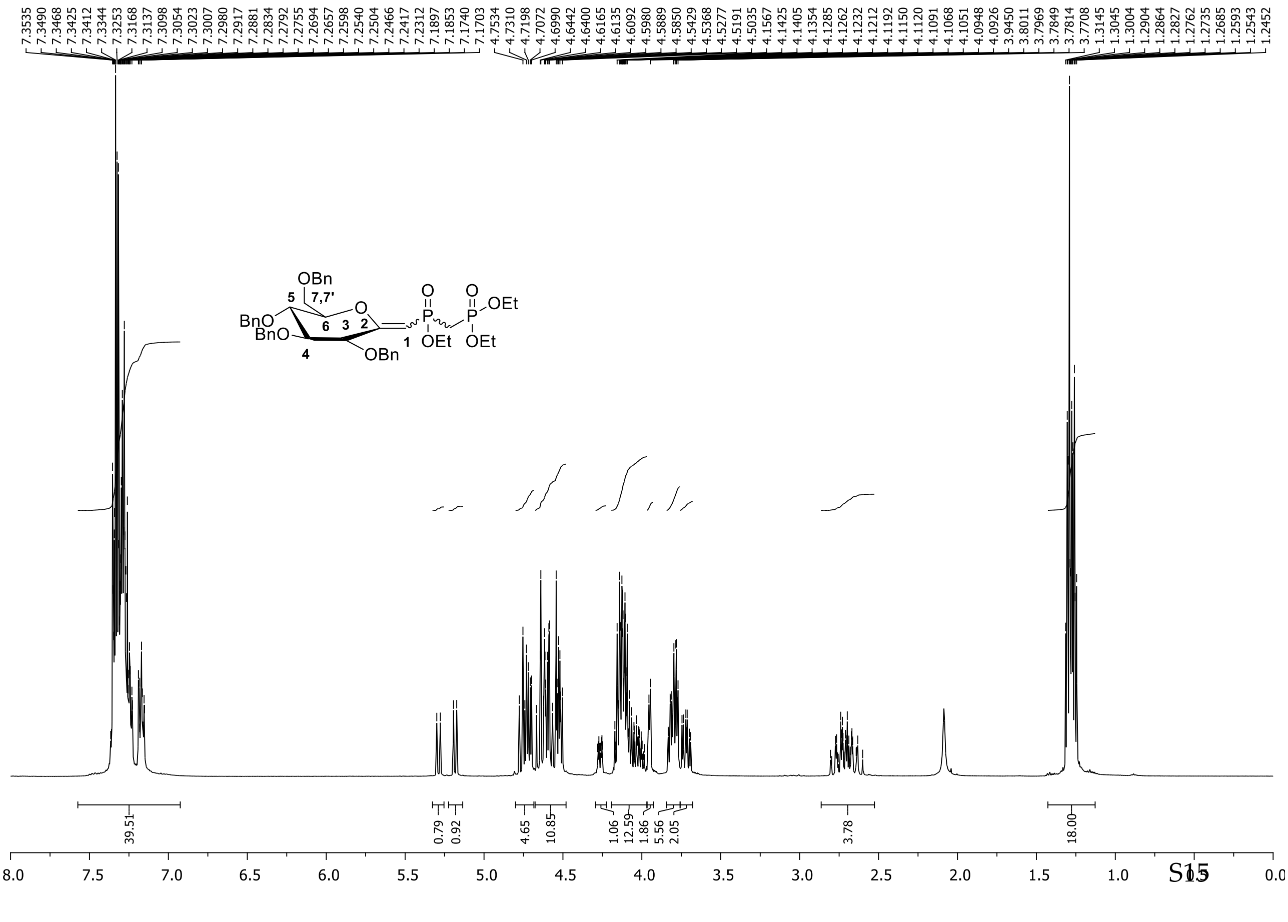

^{13}C NMR, 125 MHz ($CDCl_3$), compound **19**

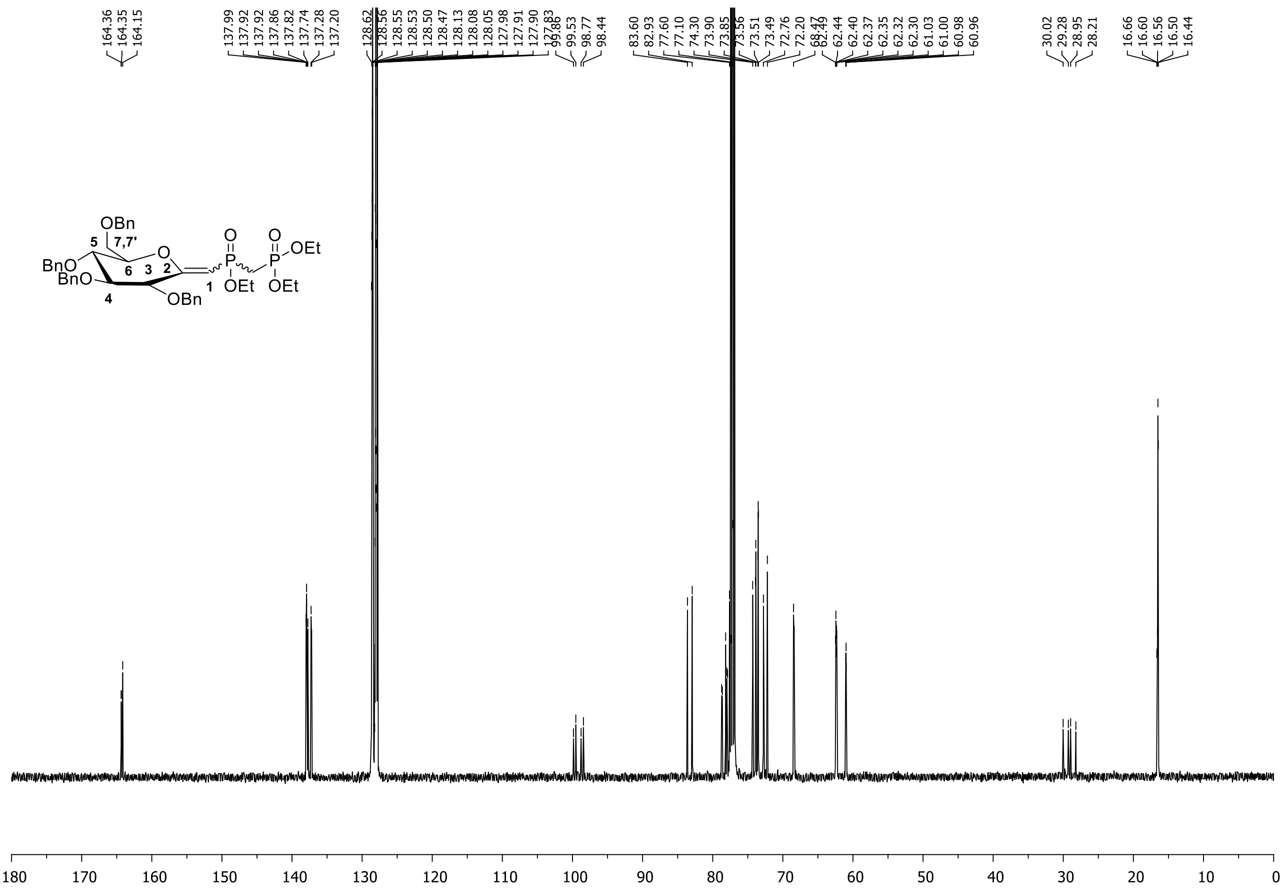

^{31}P NMR, 202 MHz ($CDCl_3$), compound **19**

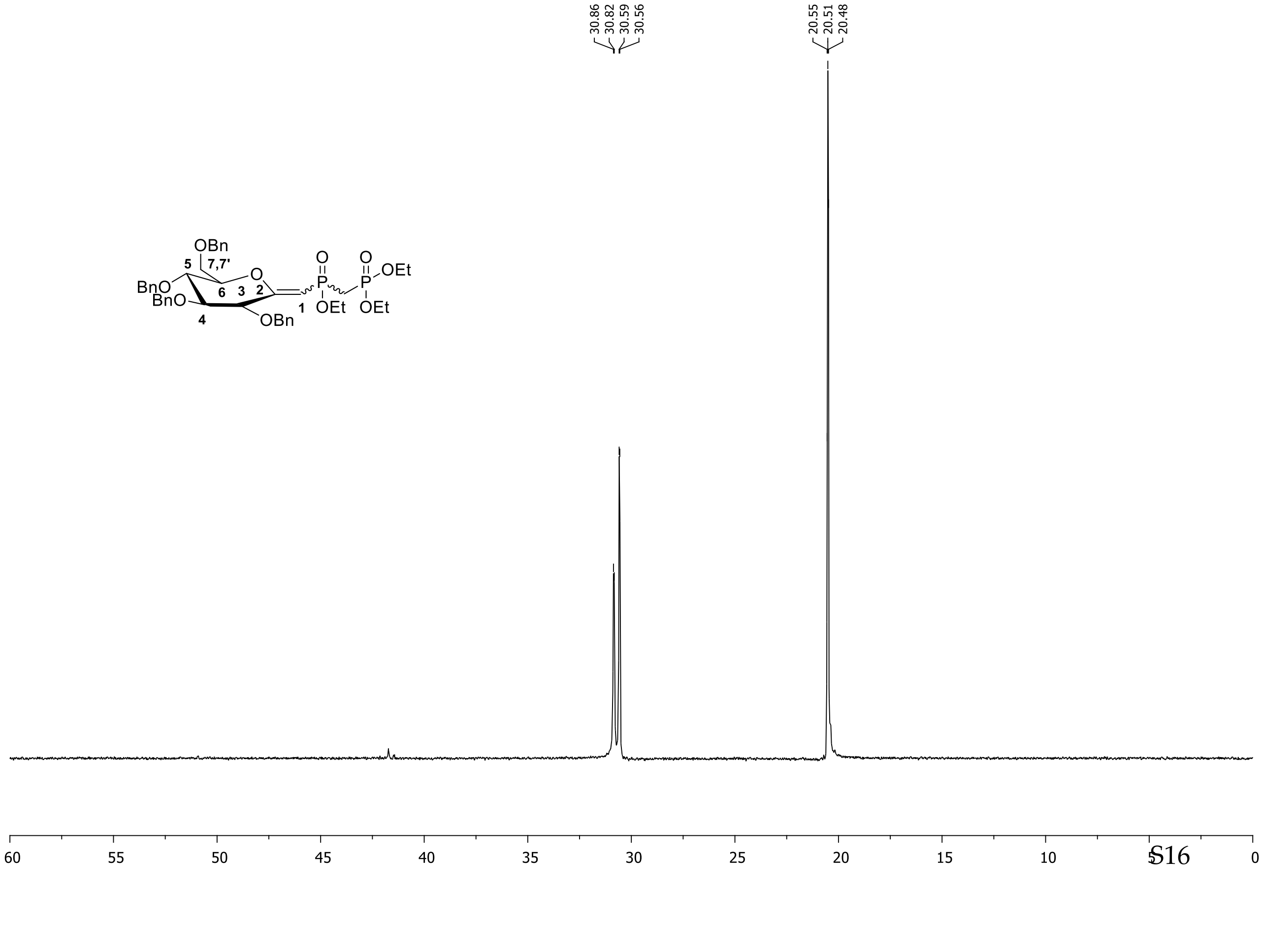

^{1}H NMR, 500 MHz ($CDCl_3$), compound **20**

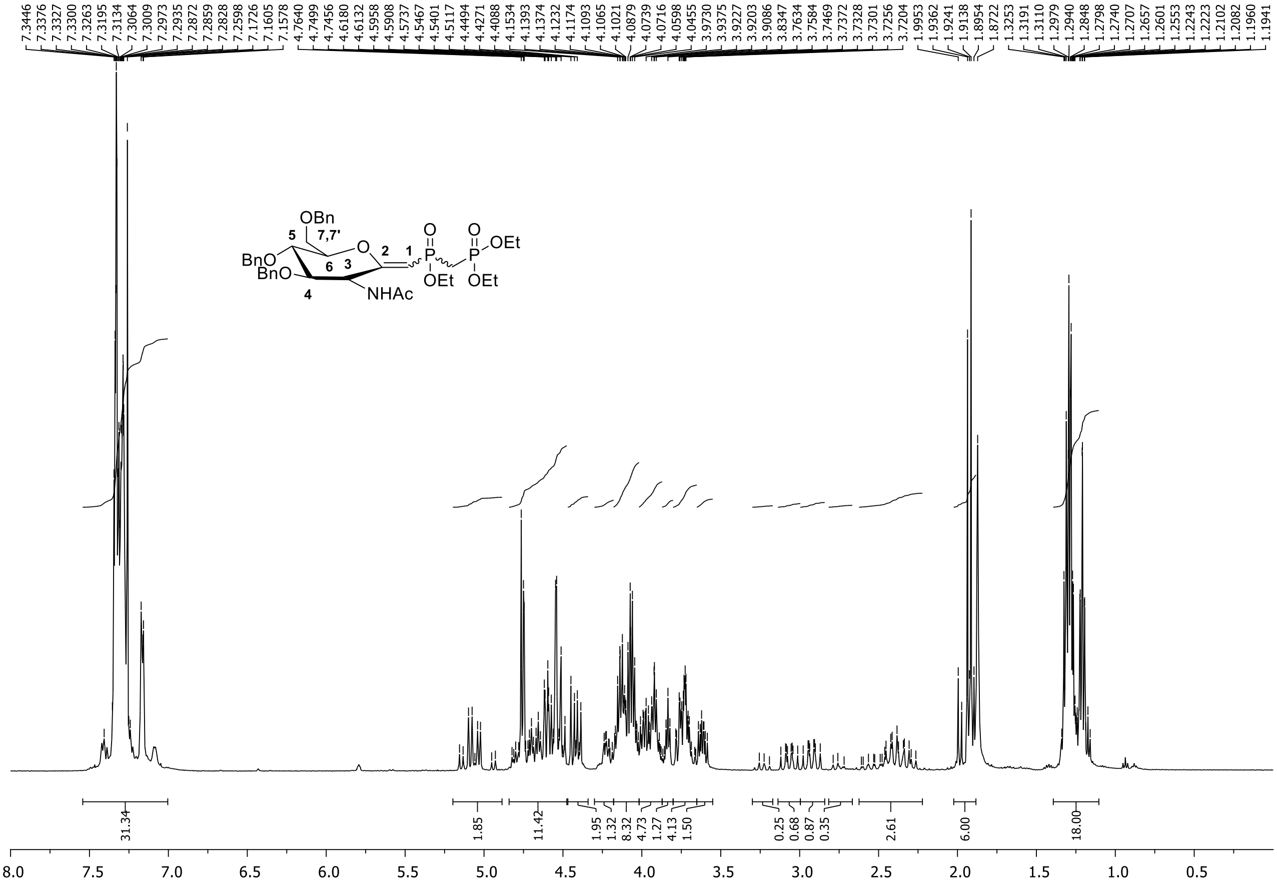

^{31}P NMR, 202 MHz ($CDCl_3$), compound **20**

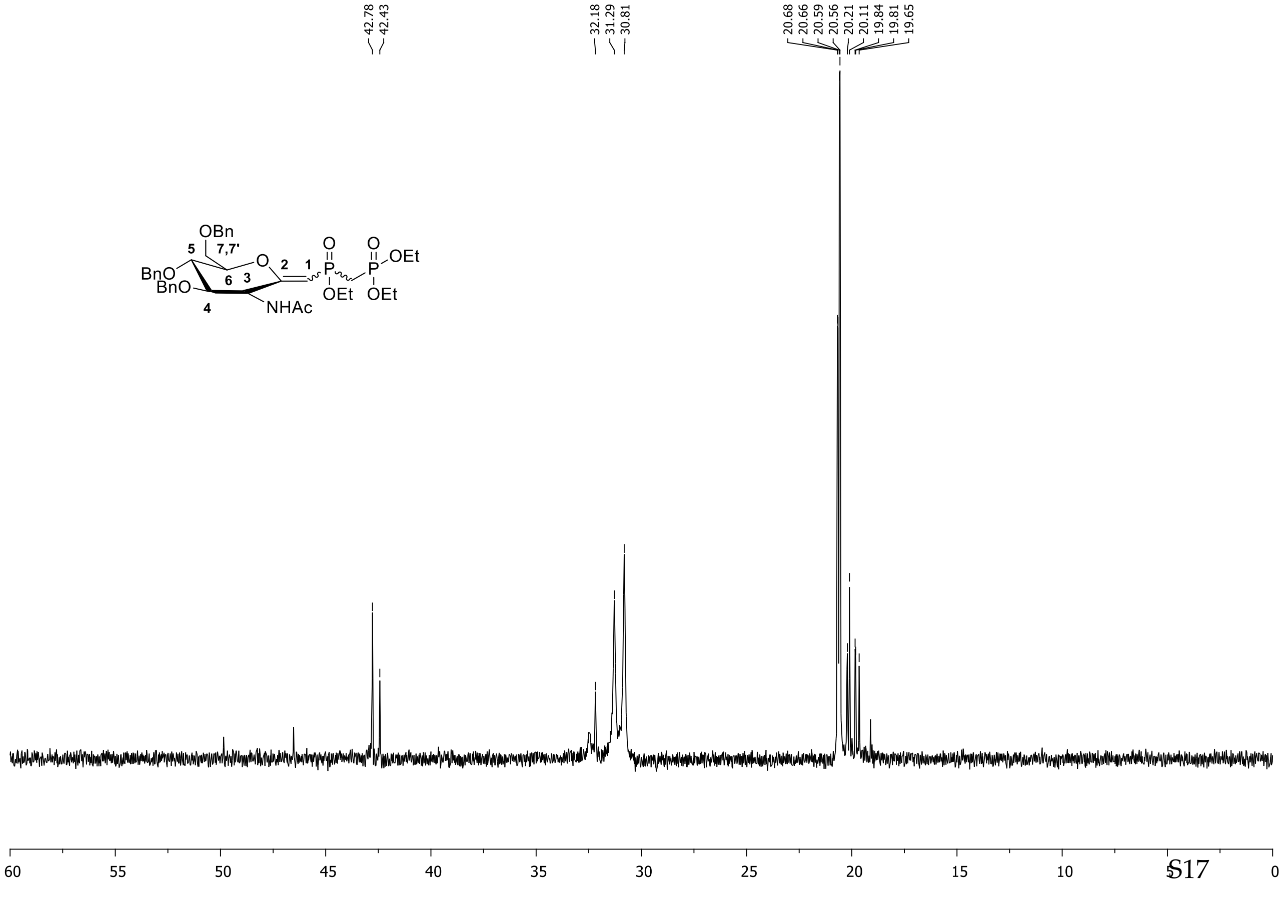

^{1}H NMR, 500 MHz (CD_3OD), compound **21**

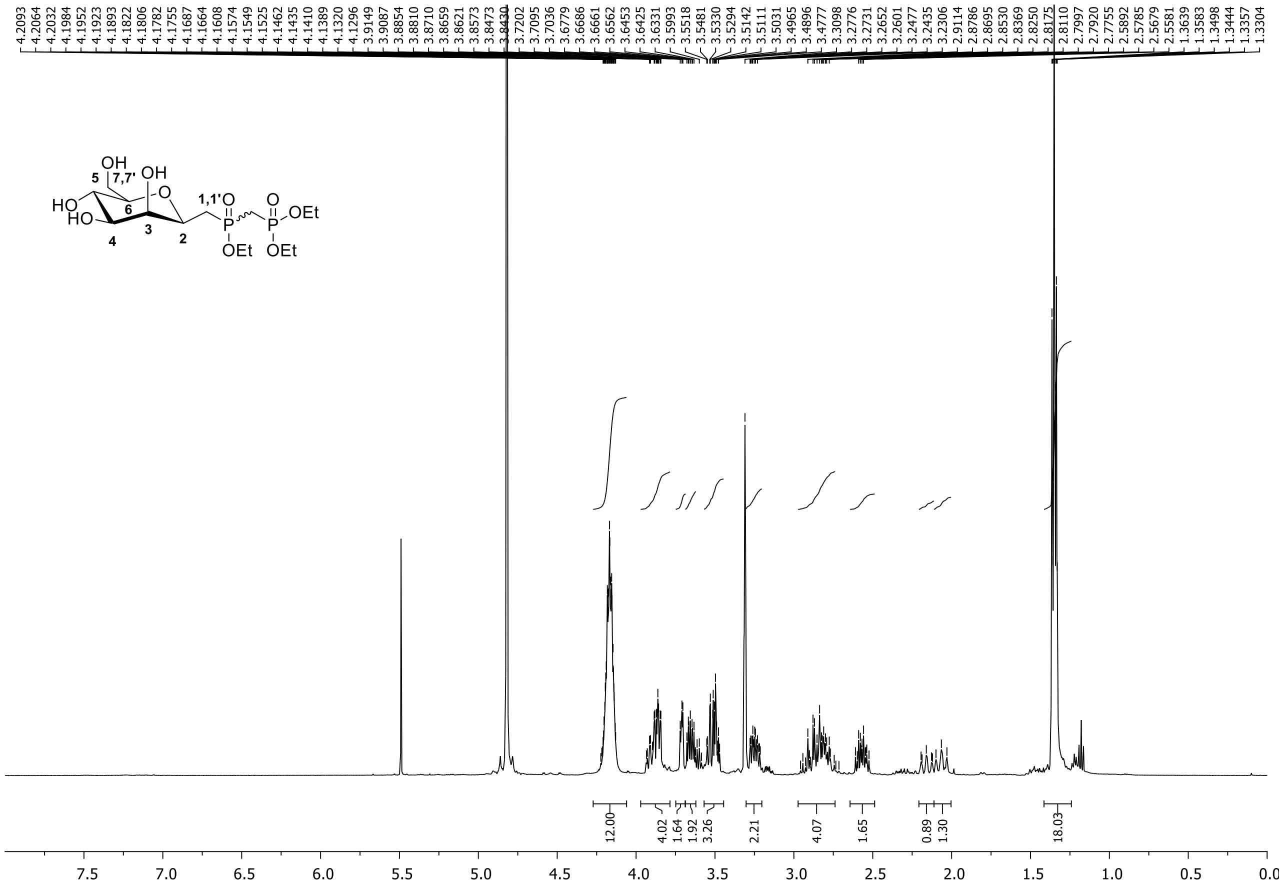

^{13}C NMR, 125 MHz (CD_3OD), compound **21**

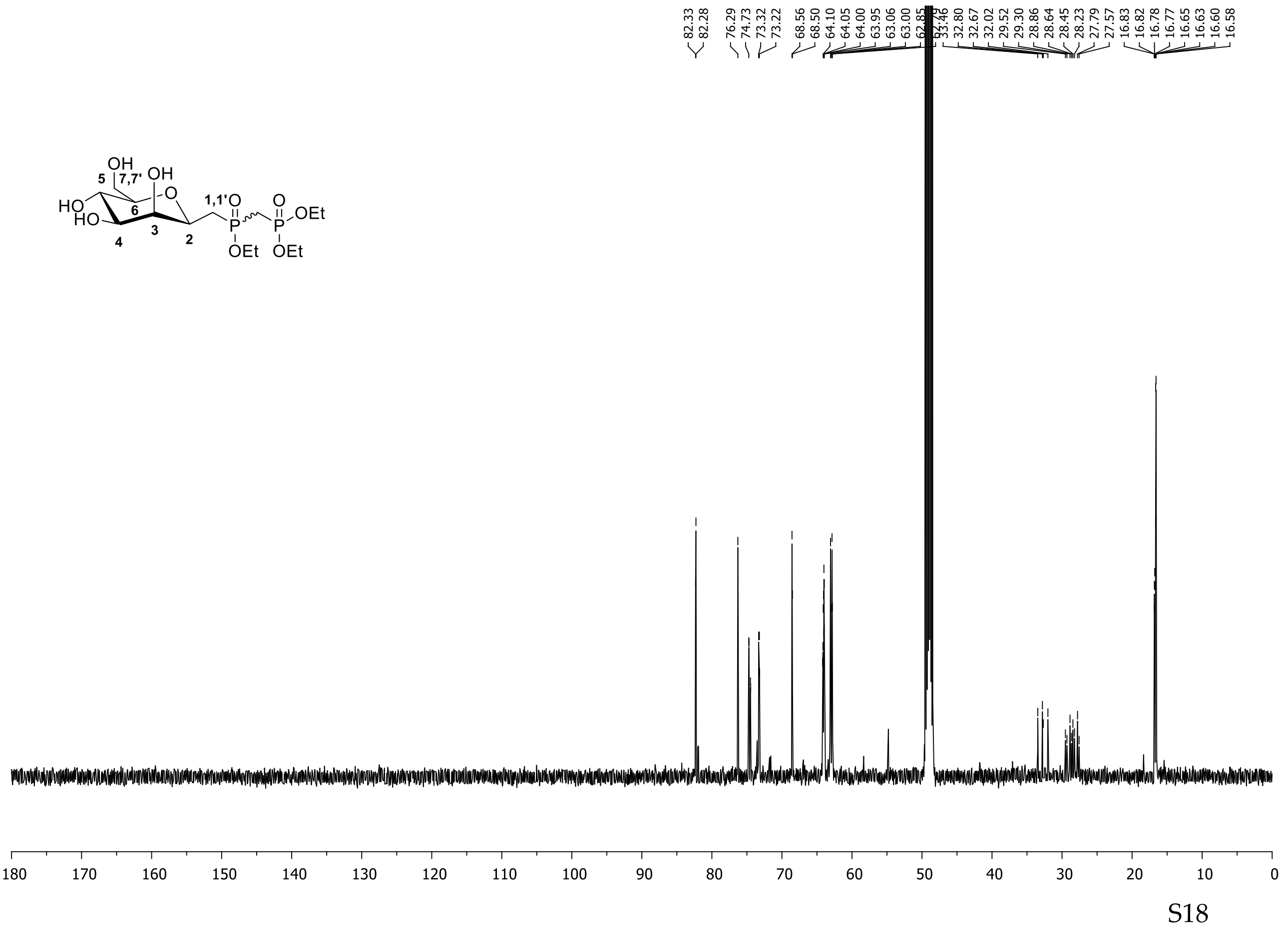

^{31}P NMR, 202 MHz (CD_3OD), compound **21**

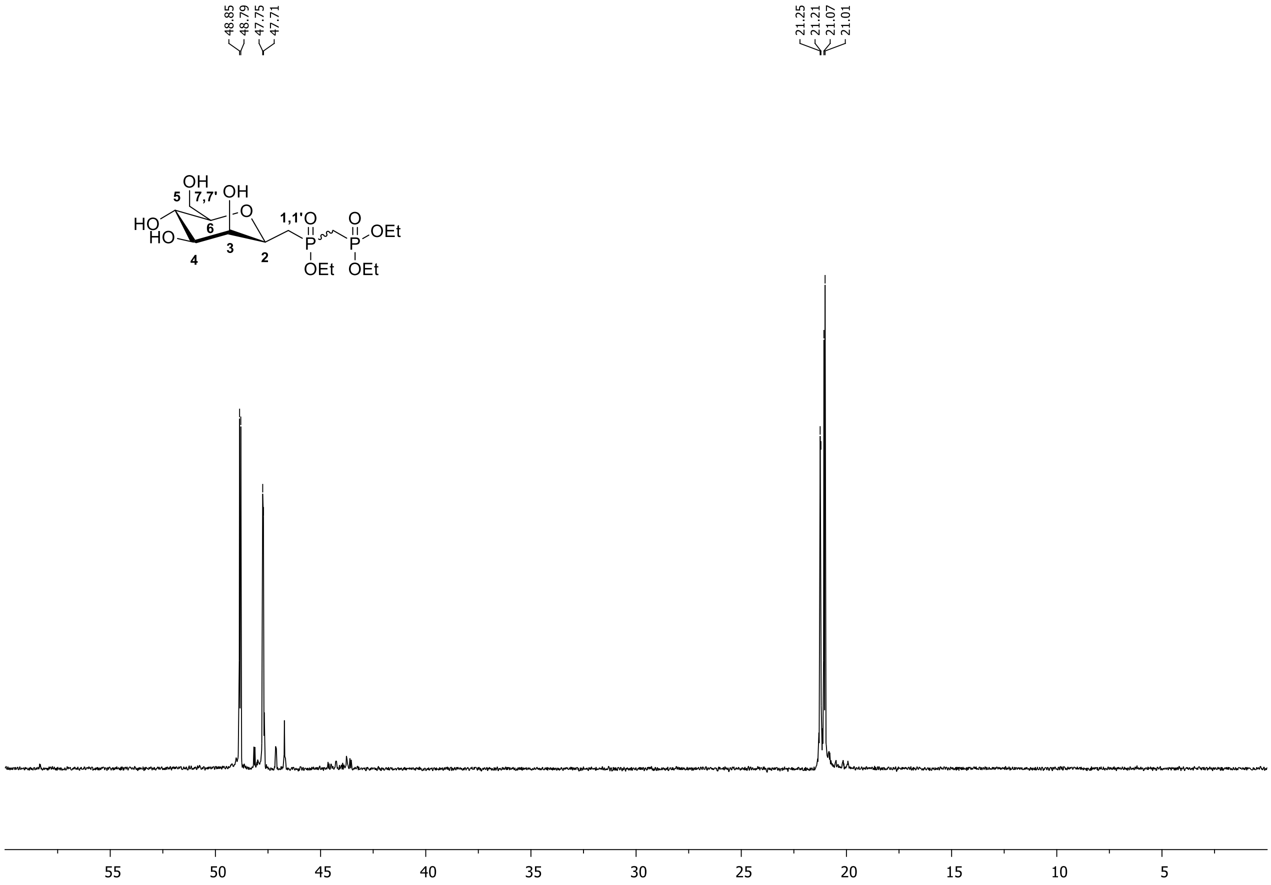

^{1}H NMR, 500 MHz (CD_3OD), compound **22**

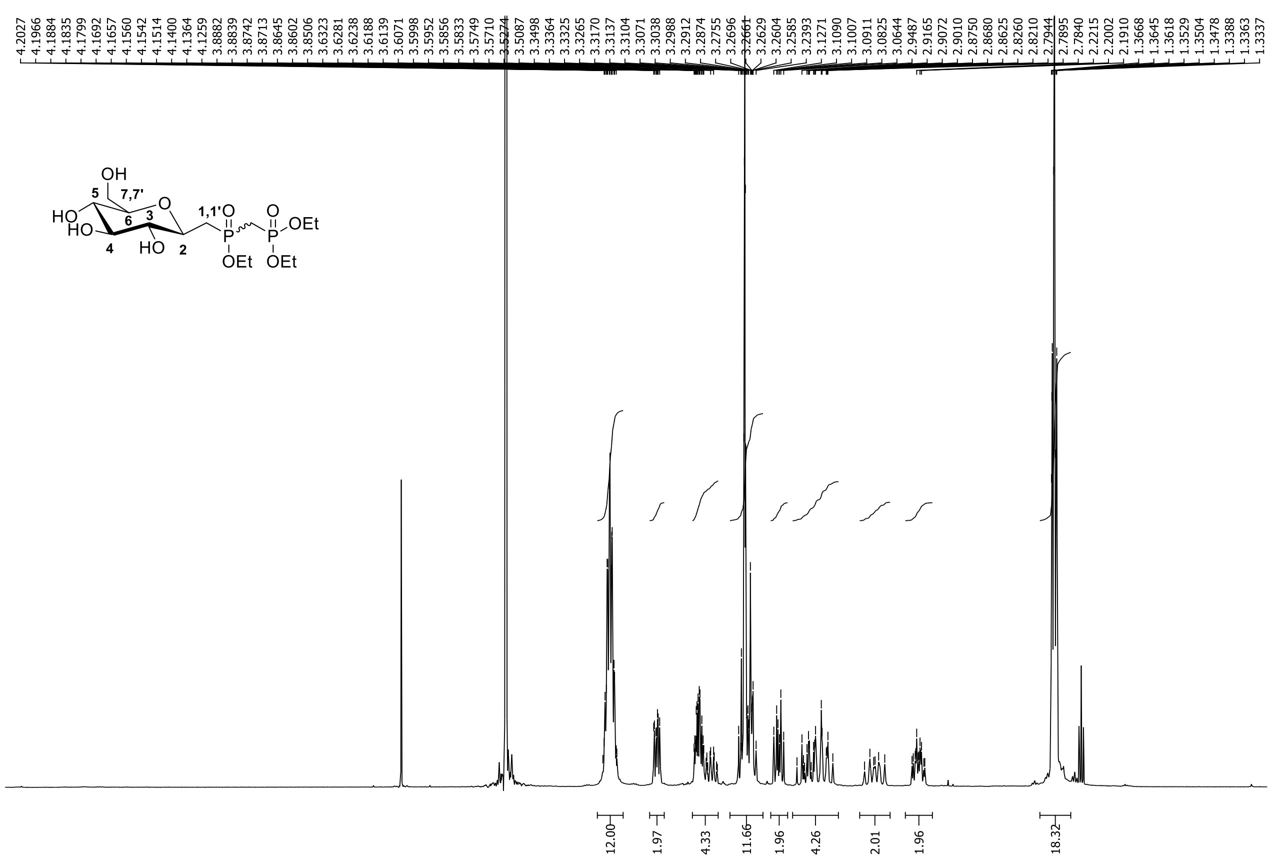

^{13}C NMR, 125 MHz (CD_3OD), compound **22**

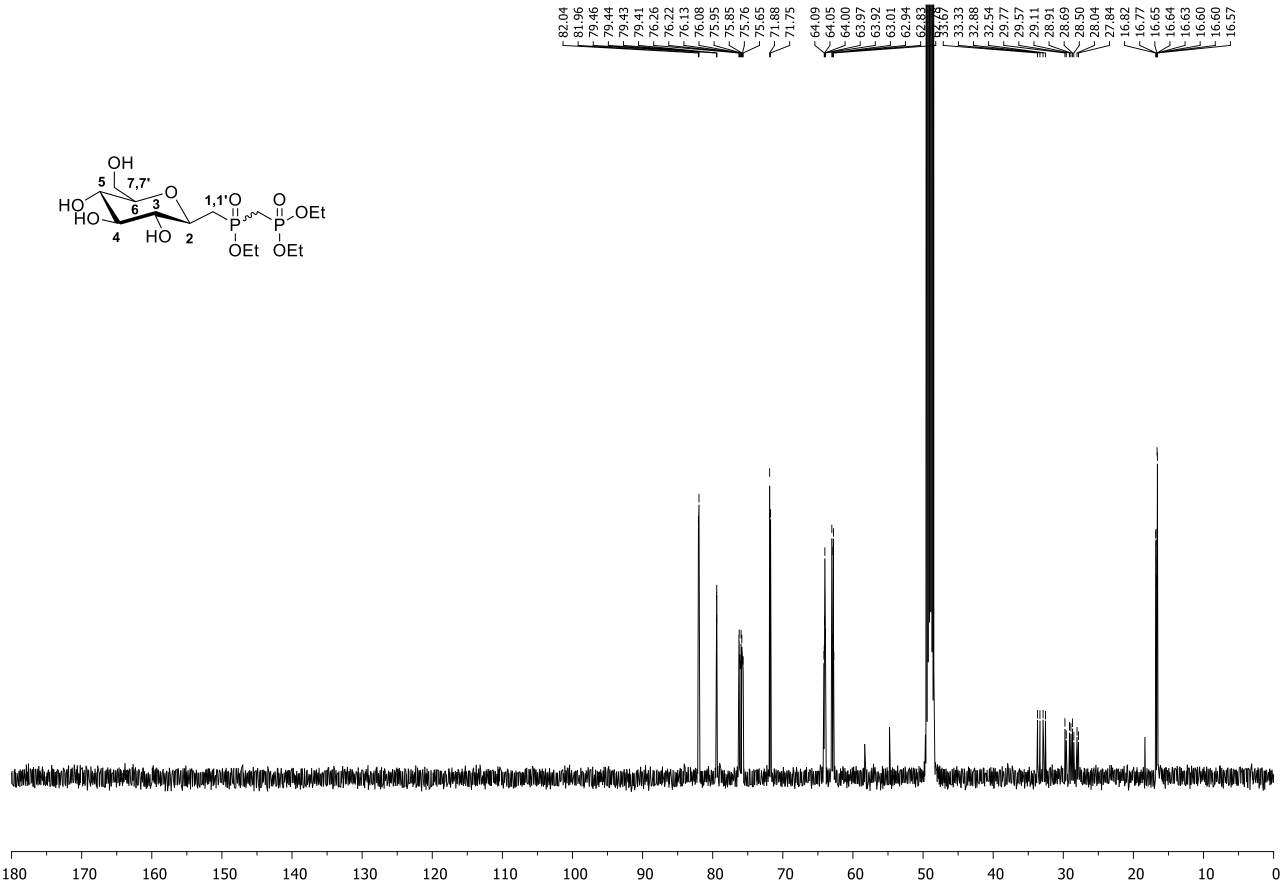

^{31}P NMR, 202 MHz (CD_3OD), compound **22**

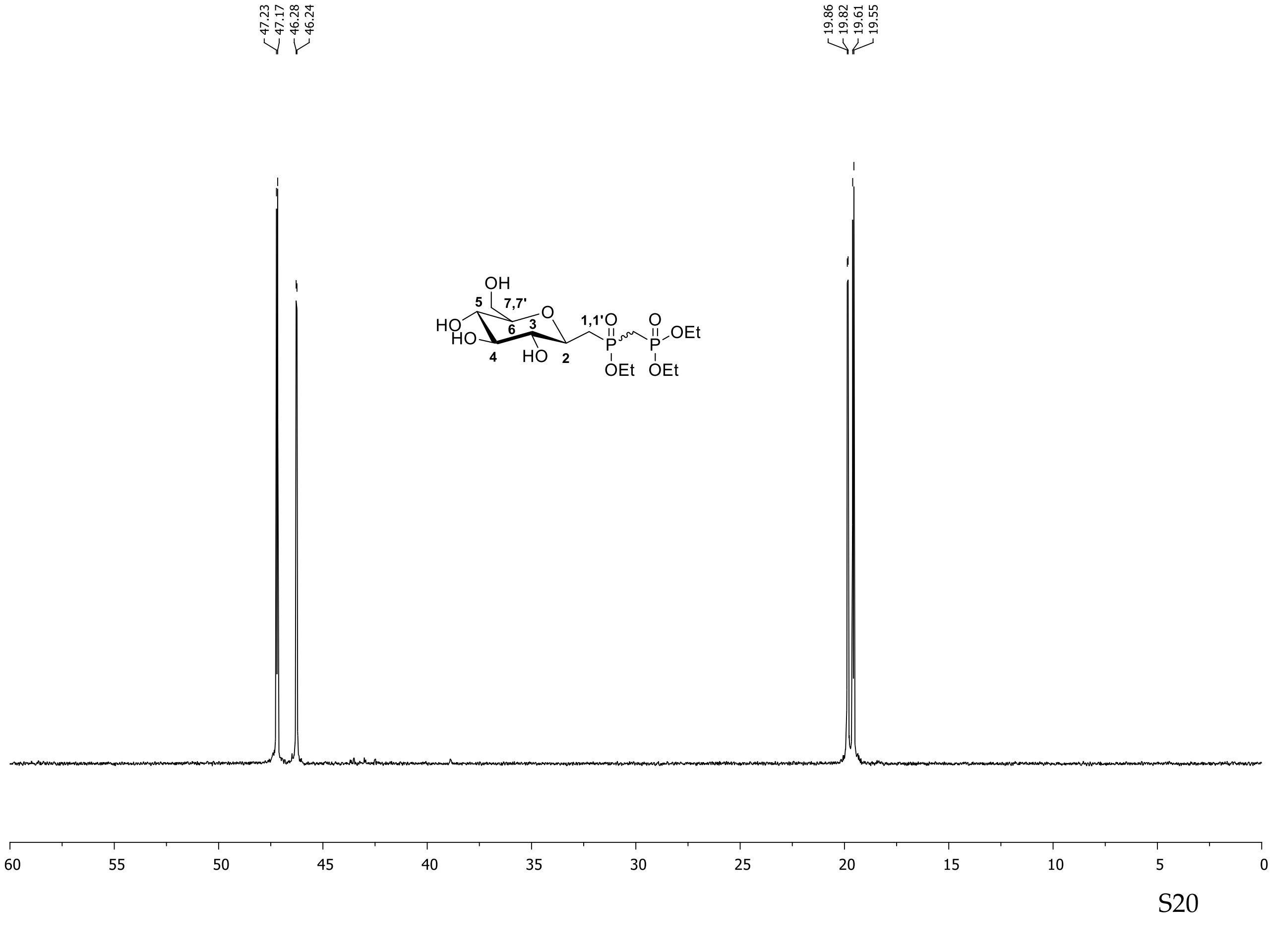

^{1}H NMR, 500 MHz (CD_3OD), compound **23**

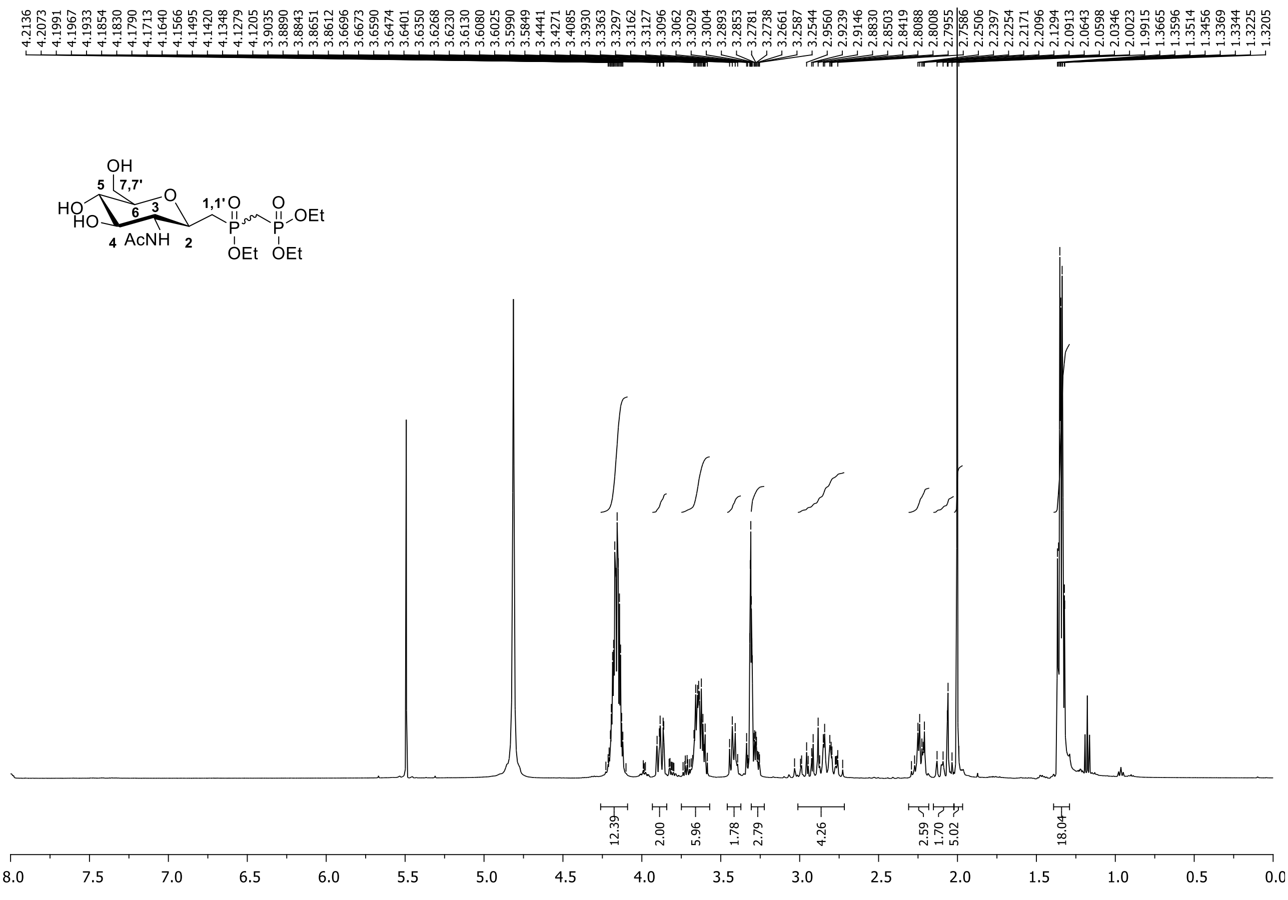

^{13}C NMR, 125 MHz (CD_3OD), compound **23**

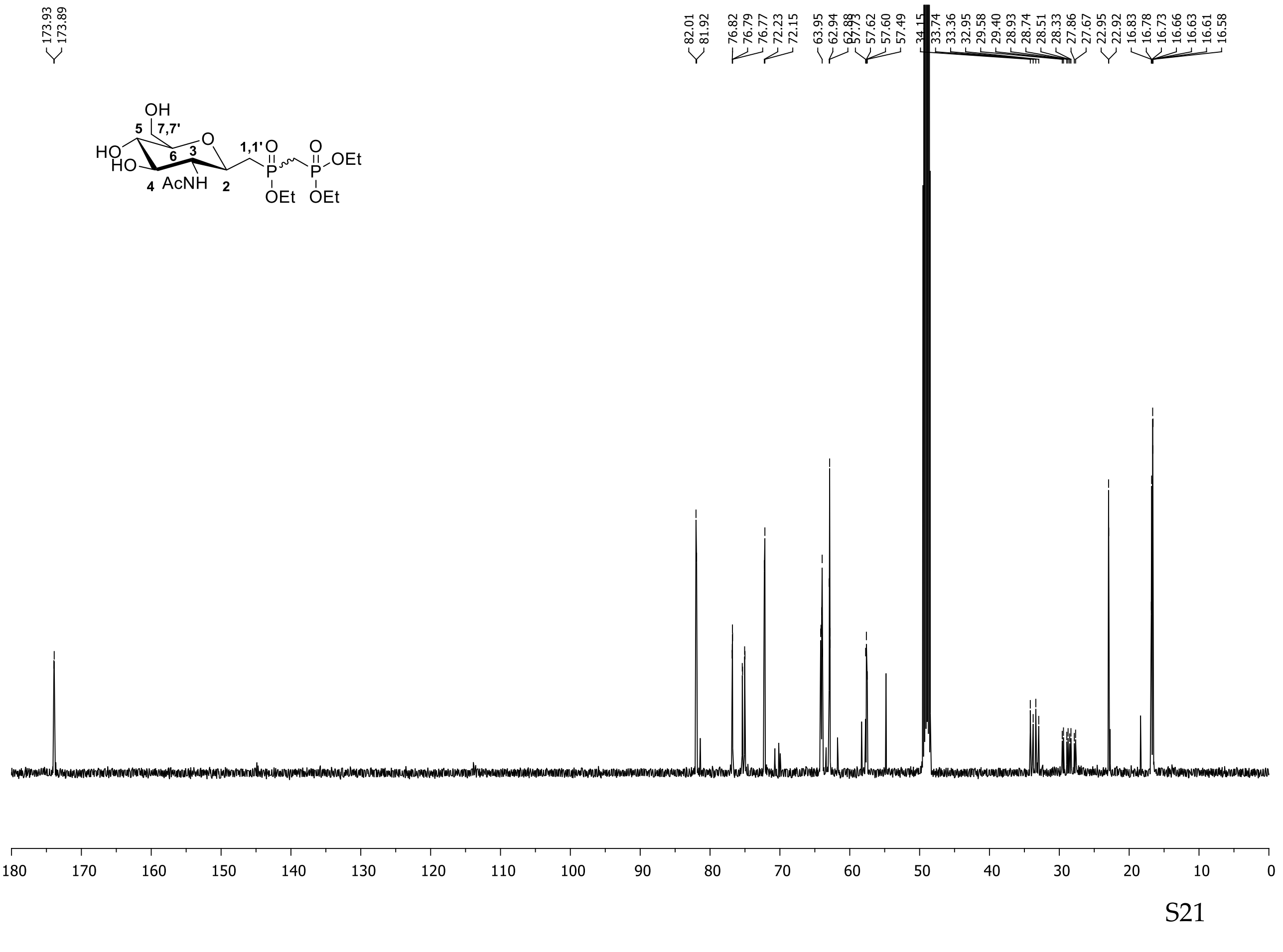

^{31}P NMR, 202 MHz (CD_3OD), compound **23**

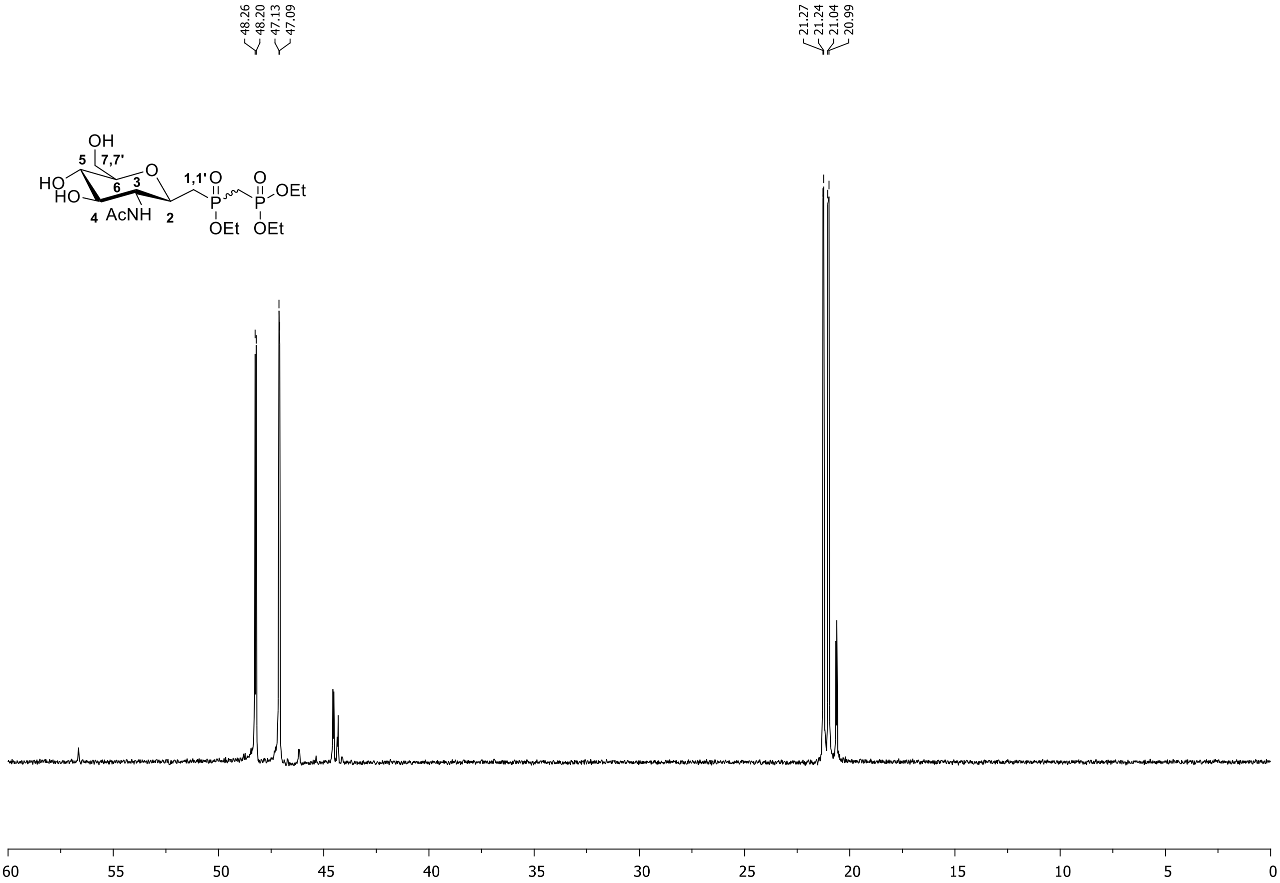

^{1}H NMR, 500 MHz ($CDCl_3$), compound **24**

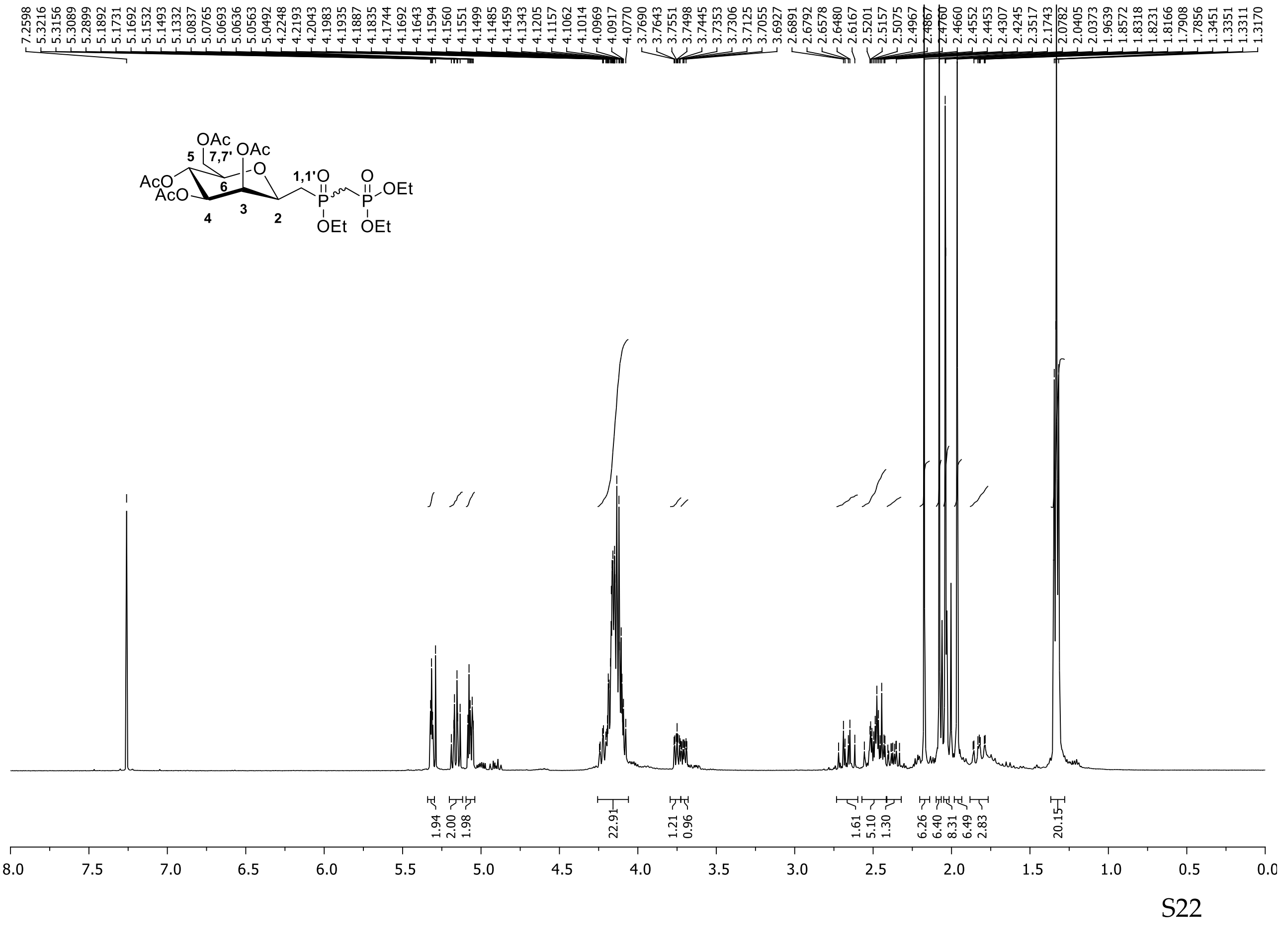

COSY and NOESY NMR, 500 MHz ($CDCl_3$), compound **24** with a pure stereogenic phosphorus

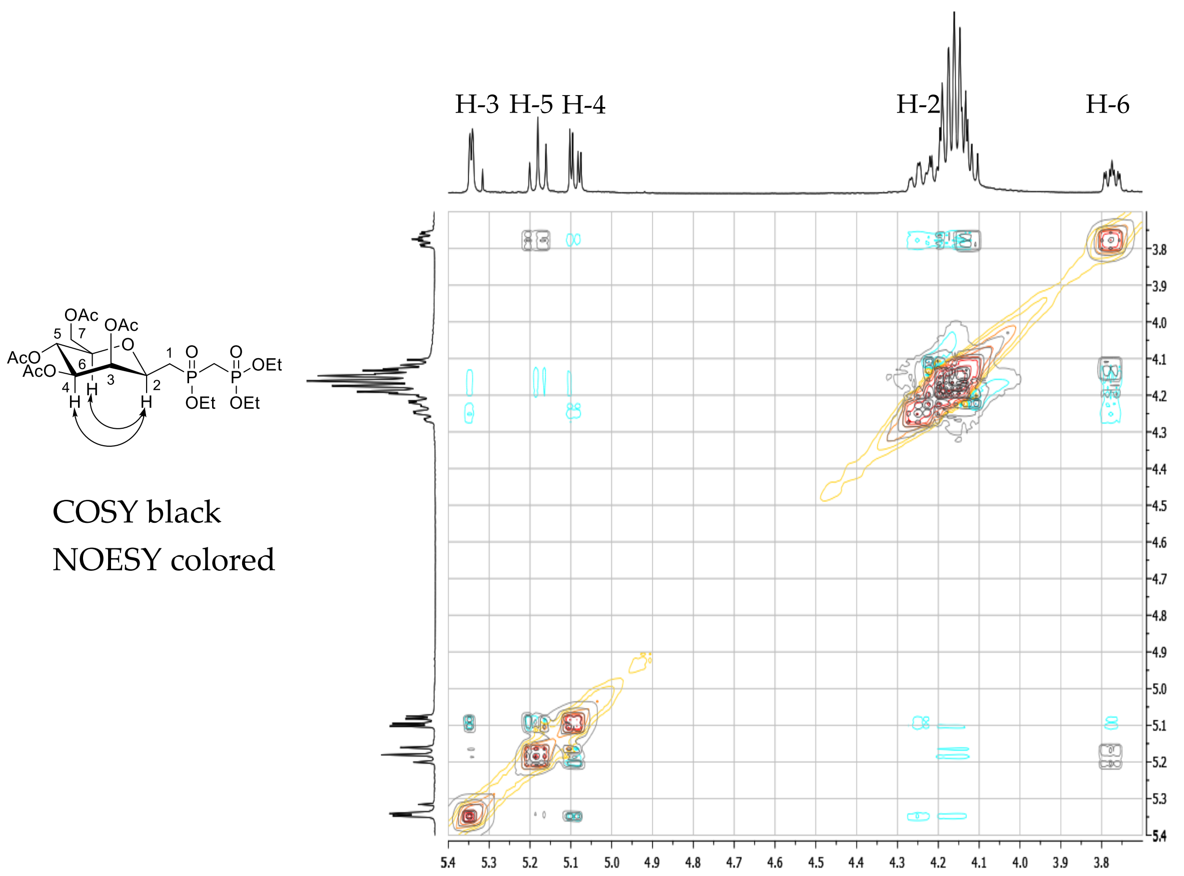

^{13}C NMR, 125 MHz ($CDCl_3$), compound **24**

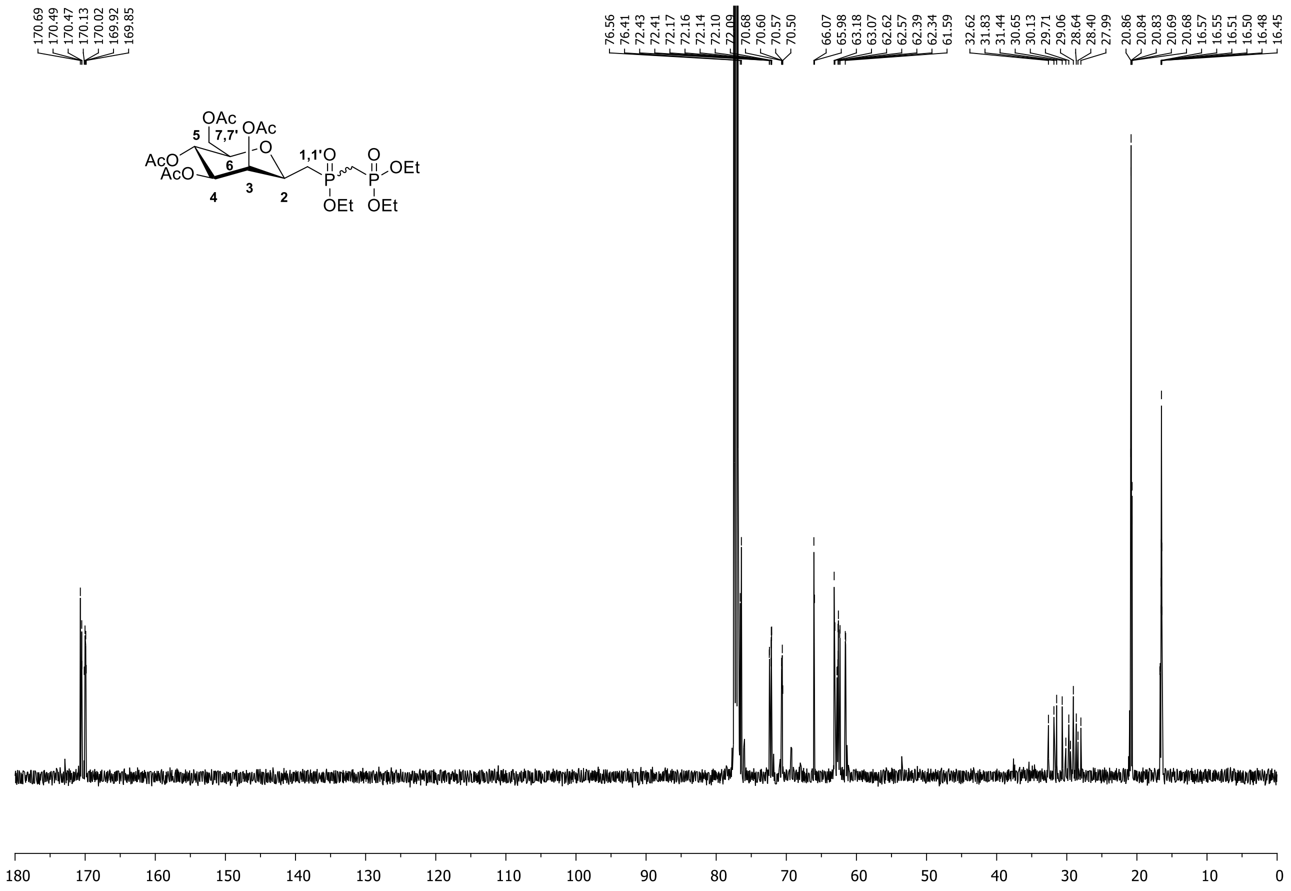

^{31}P NMR, 202 MHz (CDCl$_3$), compound **24**

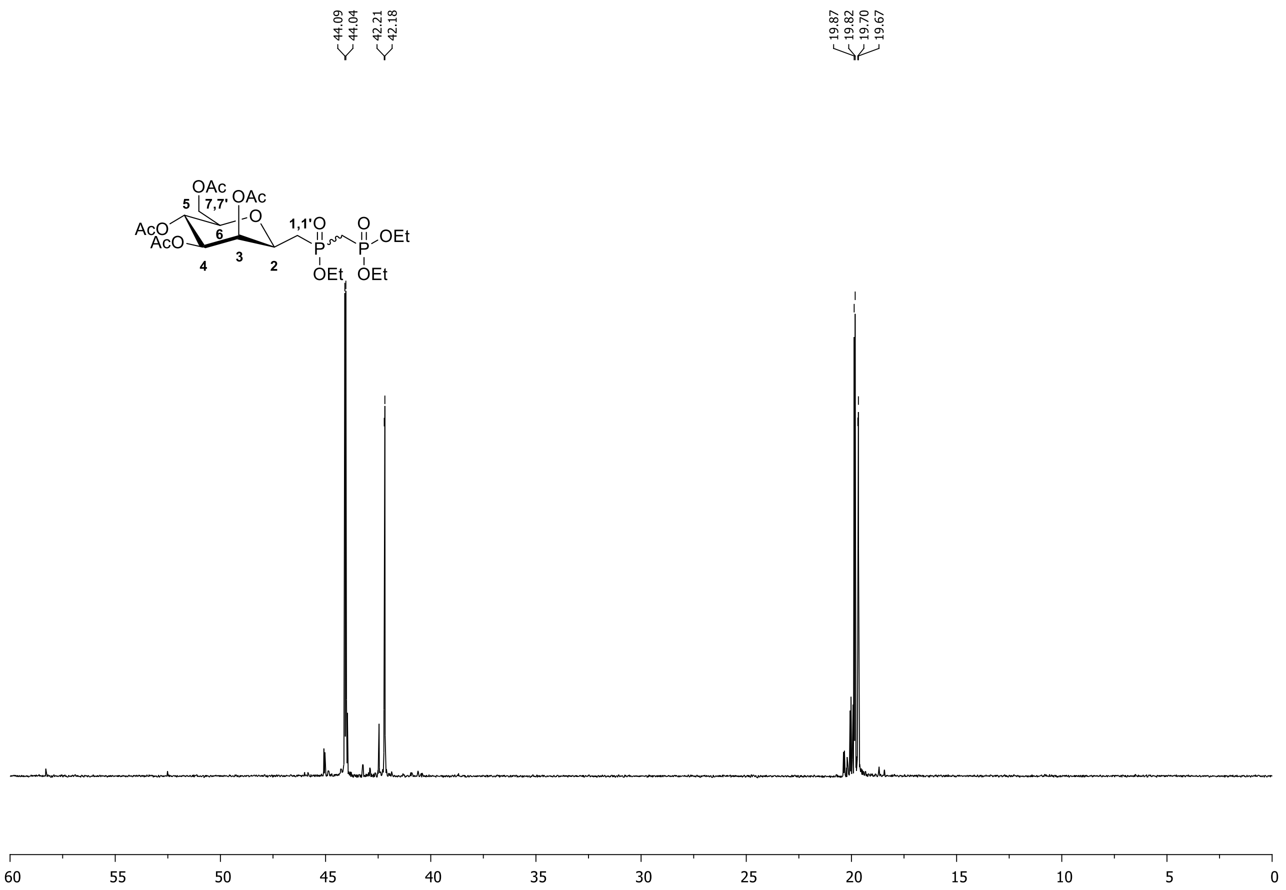

^{1}H NMR, 500 MHz (CDCl$_3$), compound **25**

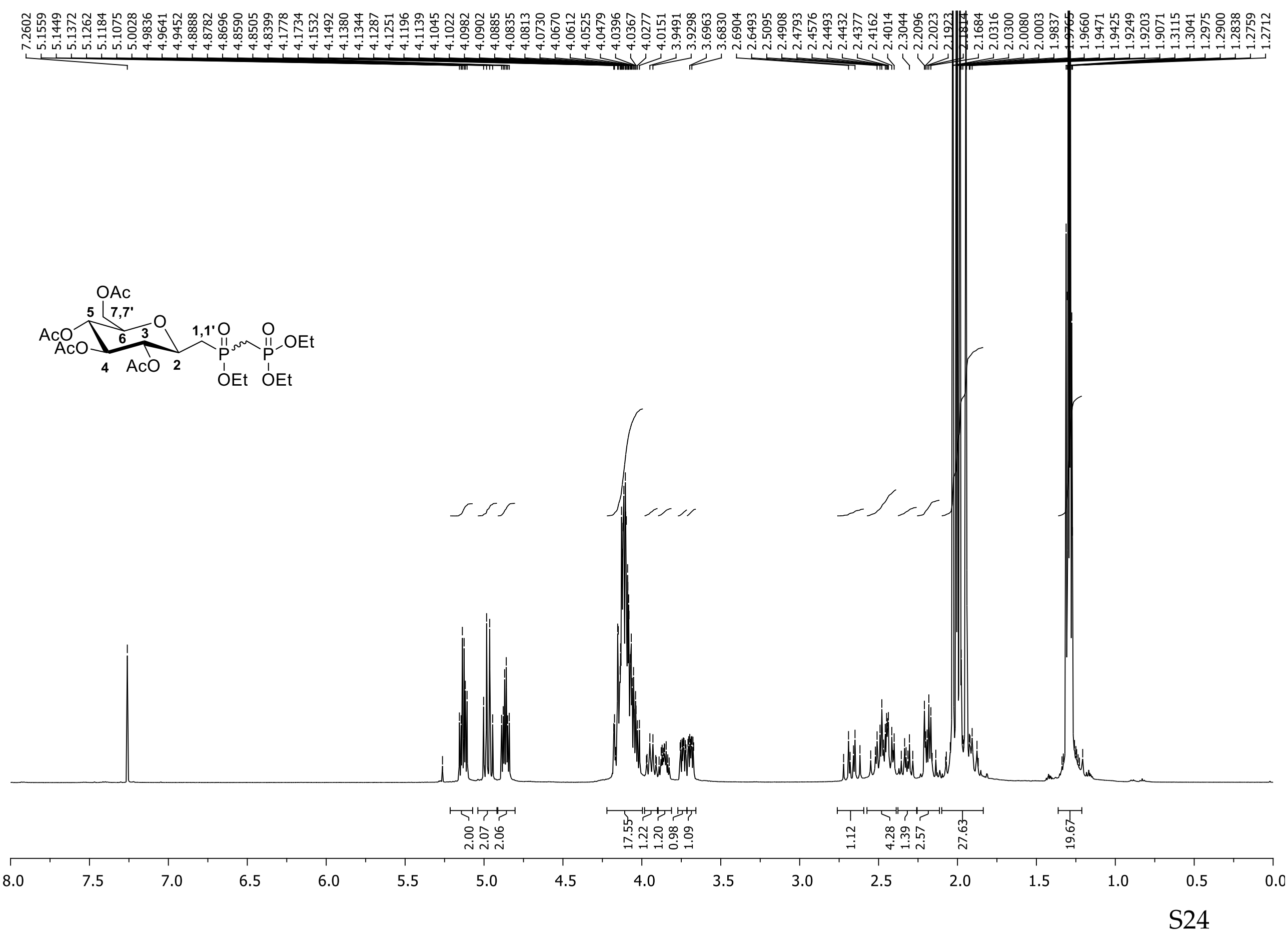

COSY and NOESY NMR, 500 MHz ($CDCl_3$), compound **25**

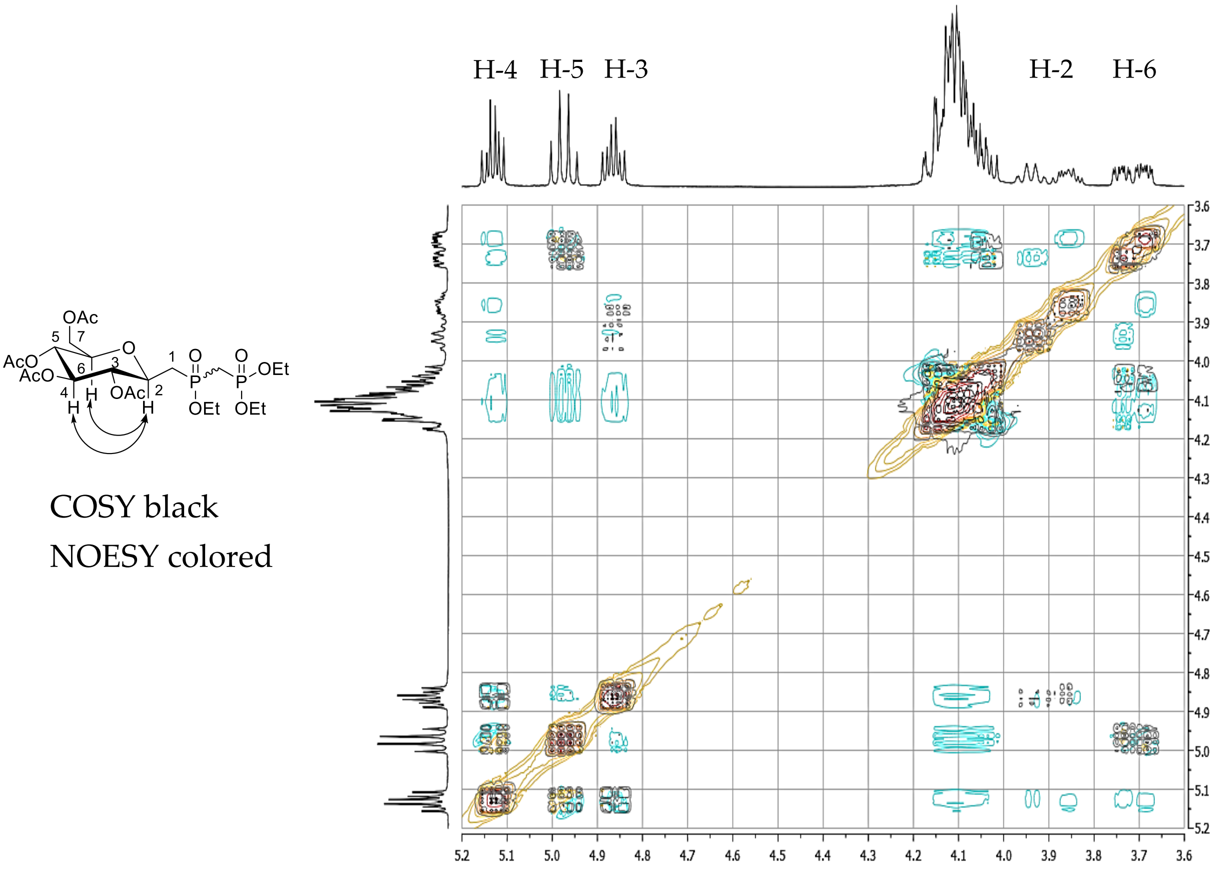

^{13}C NMR, 125 MHz ($CDCl_3$), compound **25**

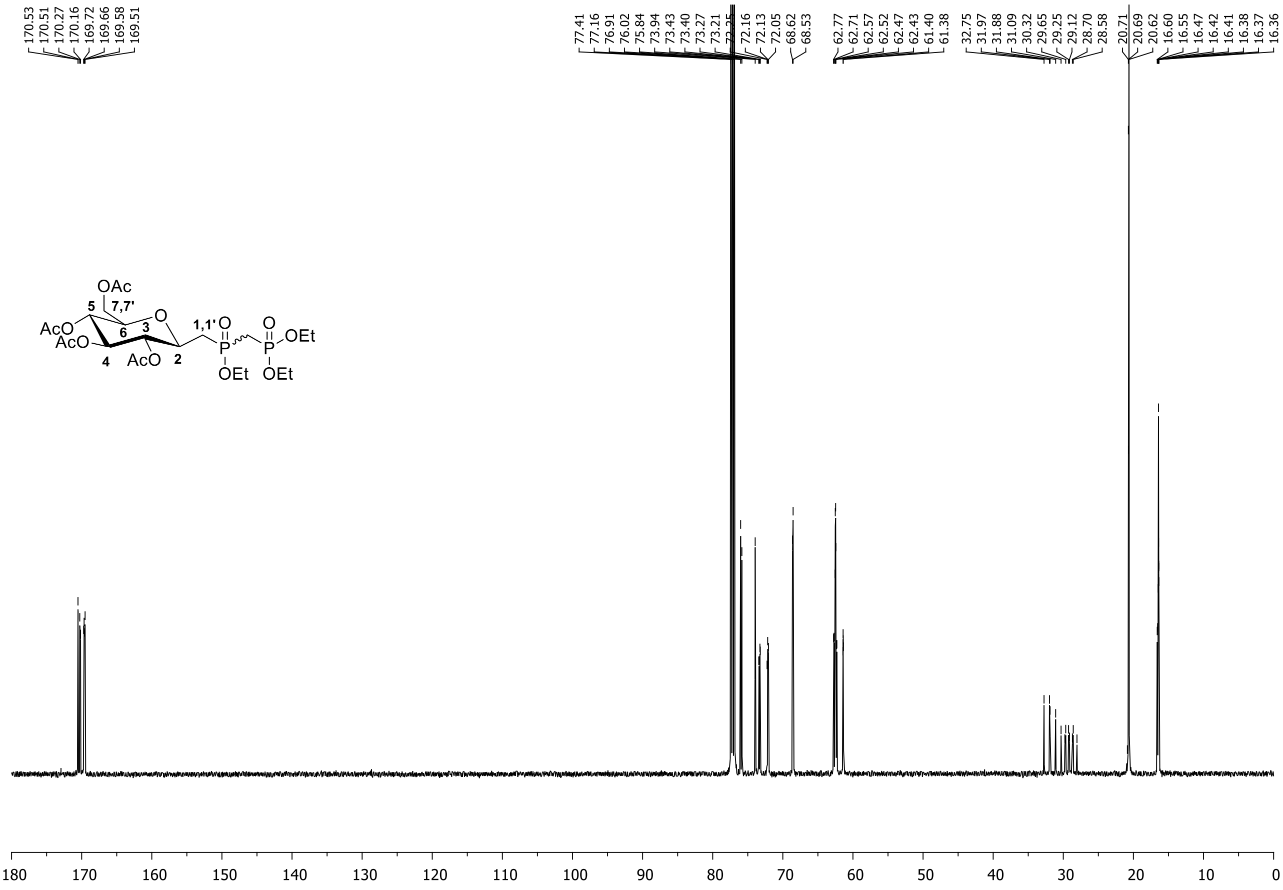

^{31}P NMR, 202 MHz ($CDCl_3$), compound **25**

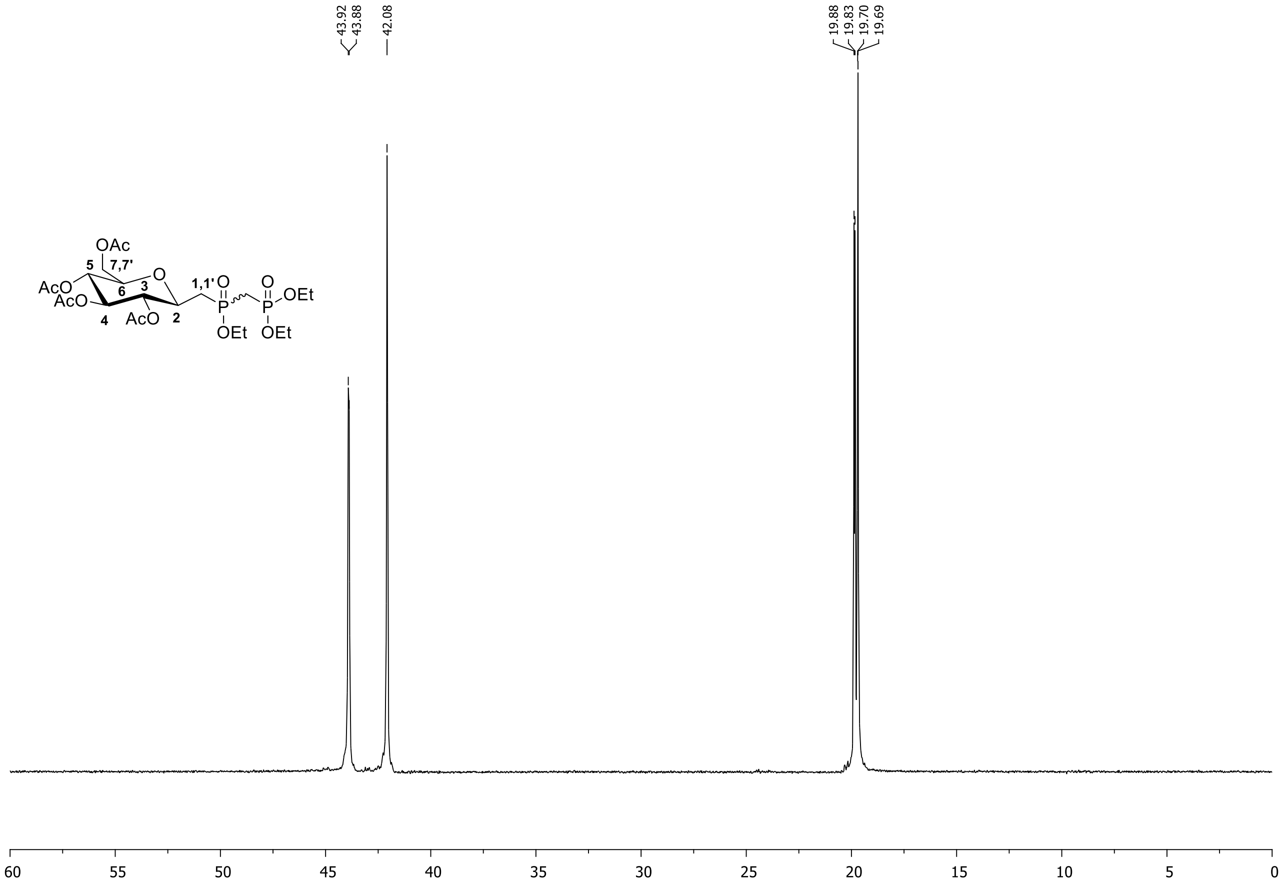

^{1}H NMR, 500 MHz ($CDCl_3$), compound **26**

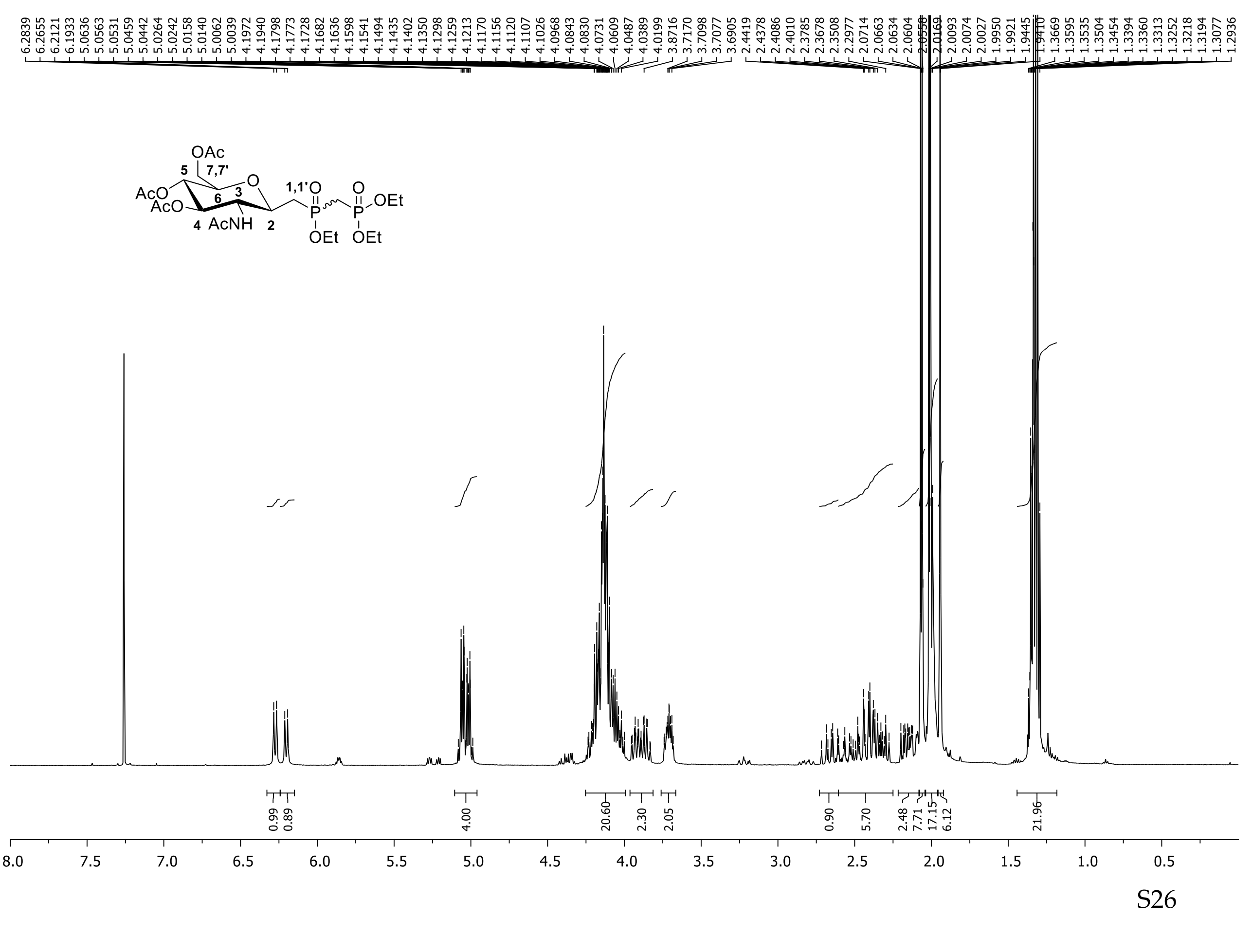

^{13}C NMR, 125 MHz ($CDCl_3$), compound **26**

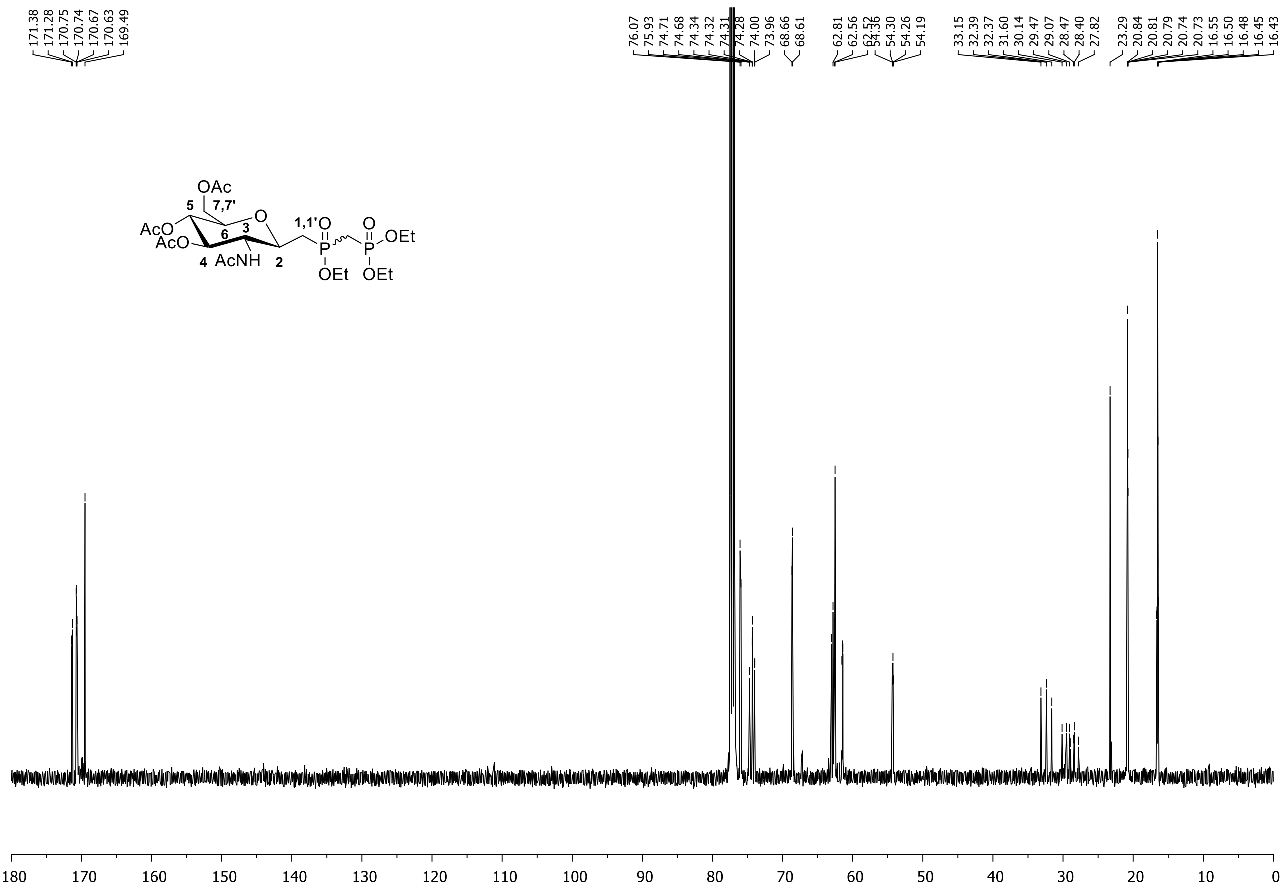

^{31}P NMR, 202 MHz ($CDCl_3$), compound **26**

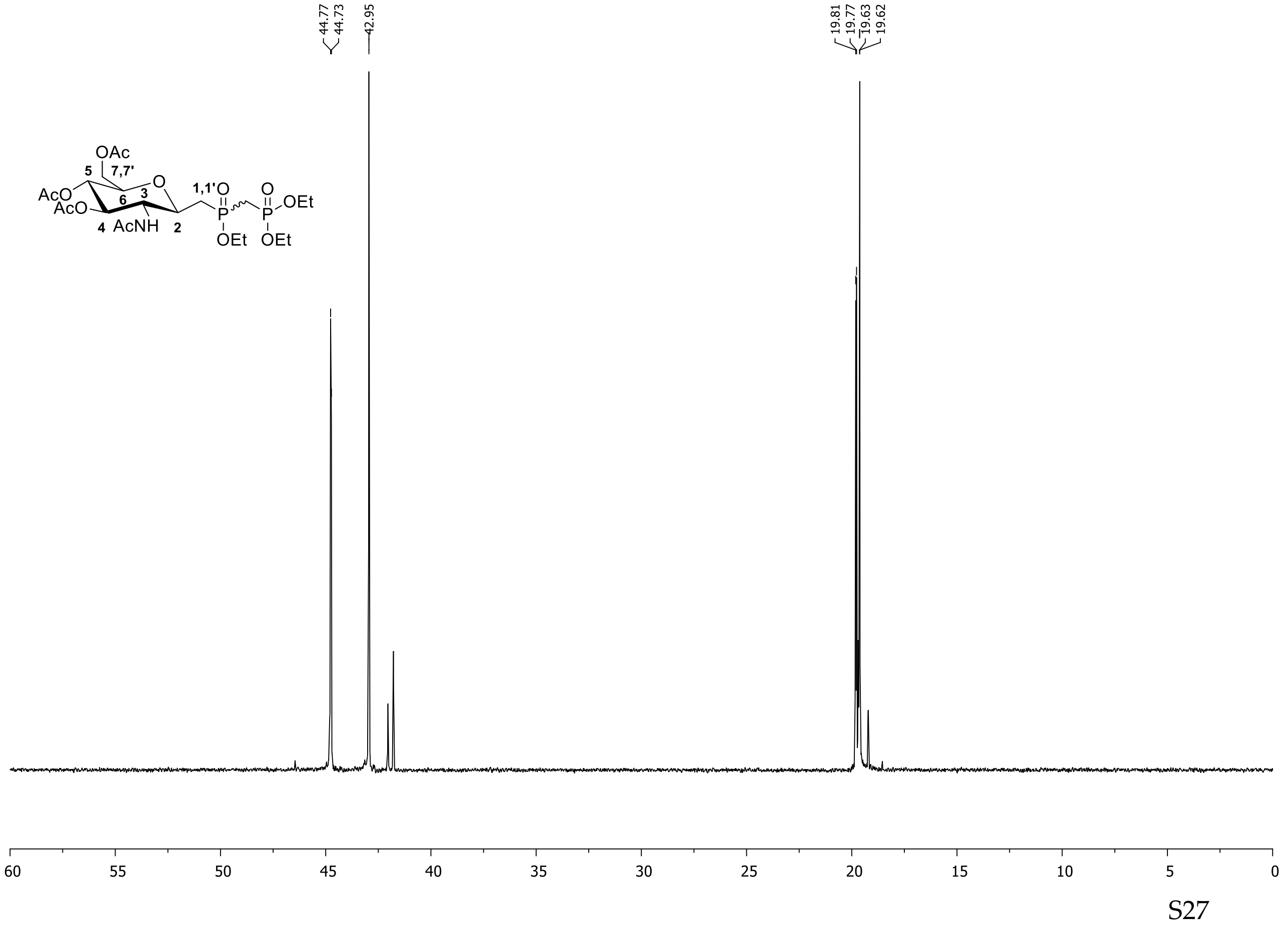

¹H NMR, 500 MHz (D₂O), compound **27**

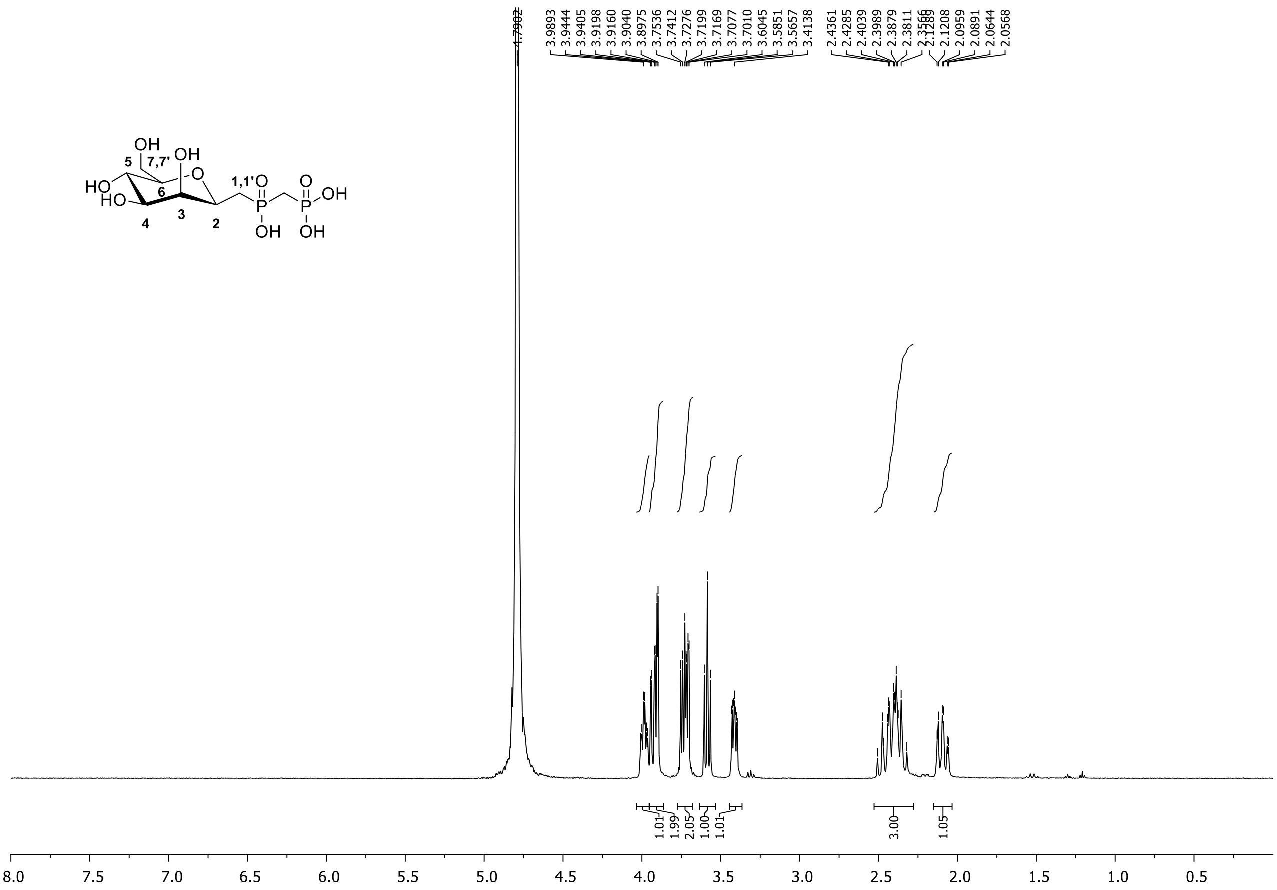

^{13}C NMR, 125 MHz (D₂O), compound **27**

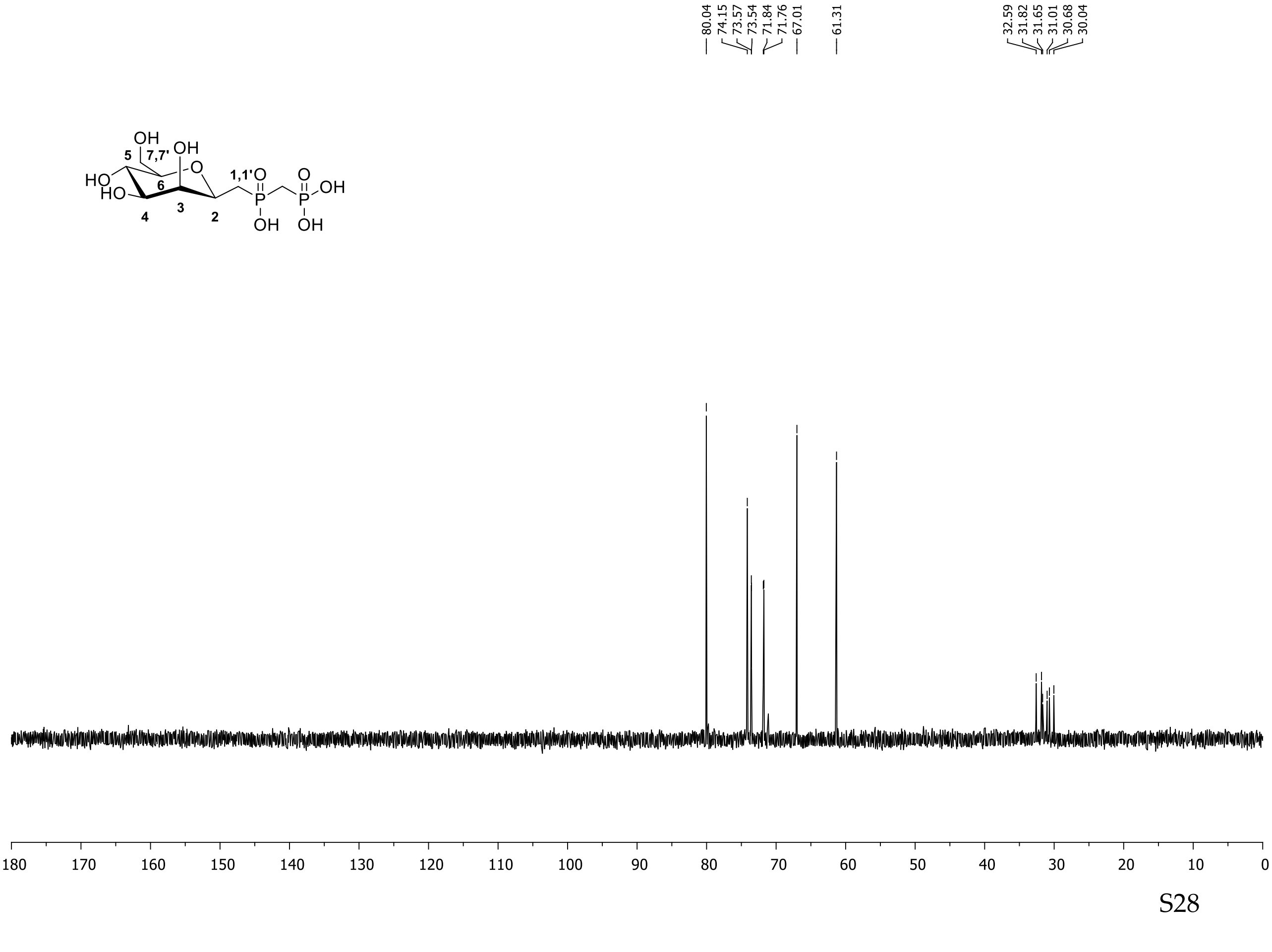

^{31}P NMR, 202 MHz (D_2O), compound **27**

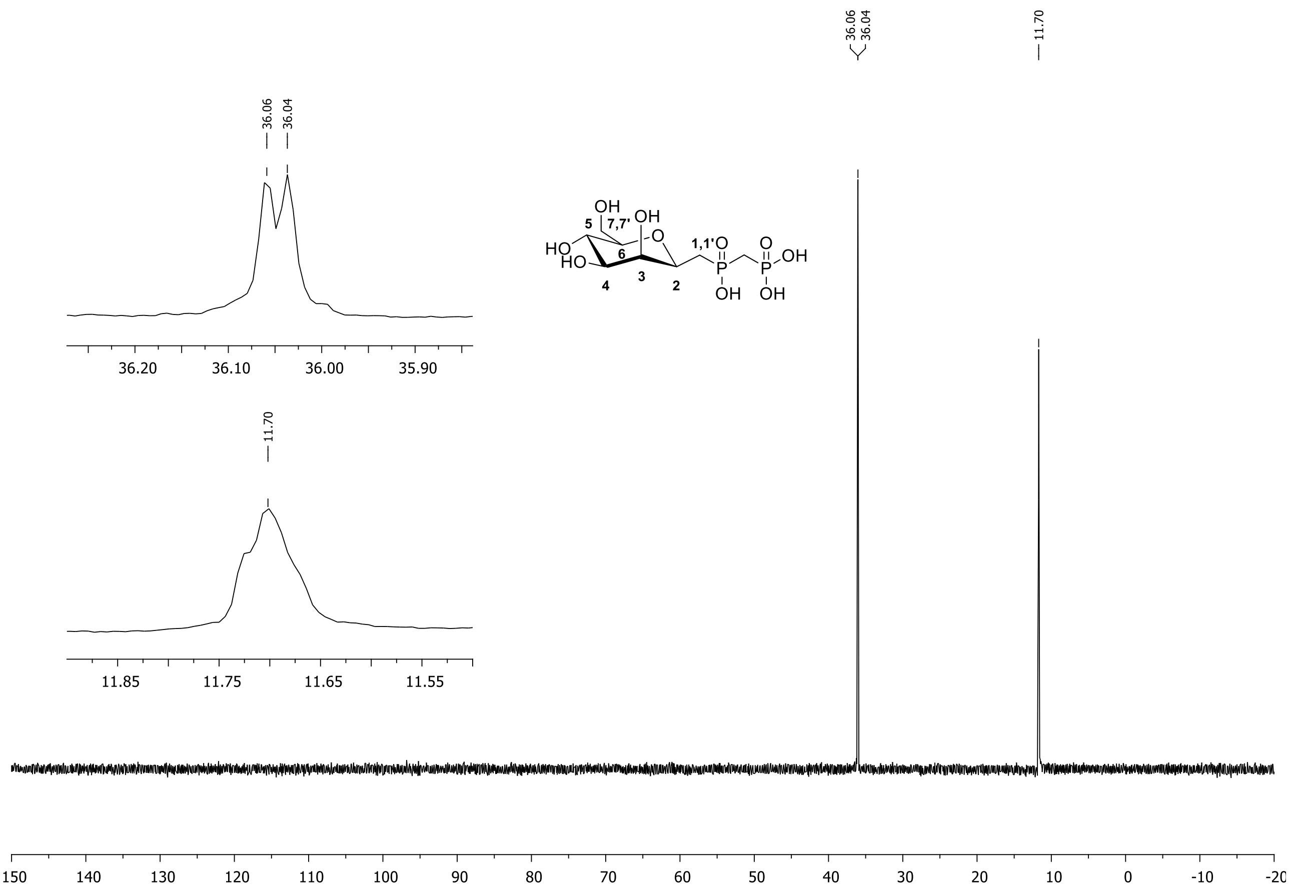

^{31}P NMR non decoupled, 202 MHz (D_2O), compound **27**

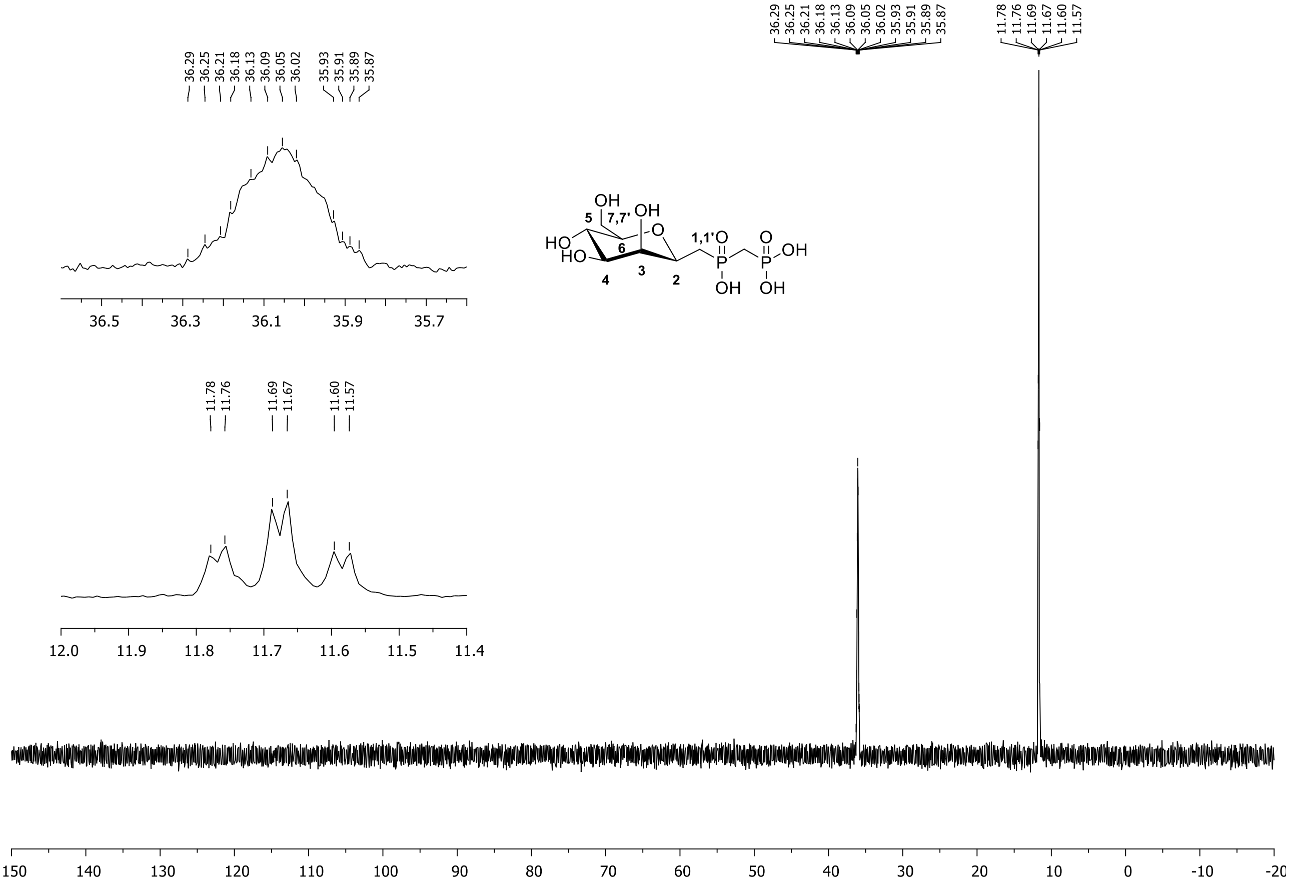

^{1}H NMR, 500 MHz (D_2O), compound **28**

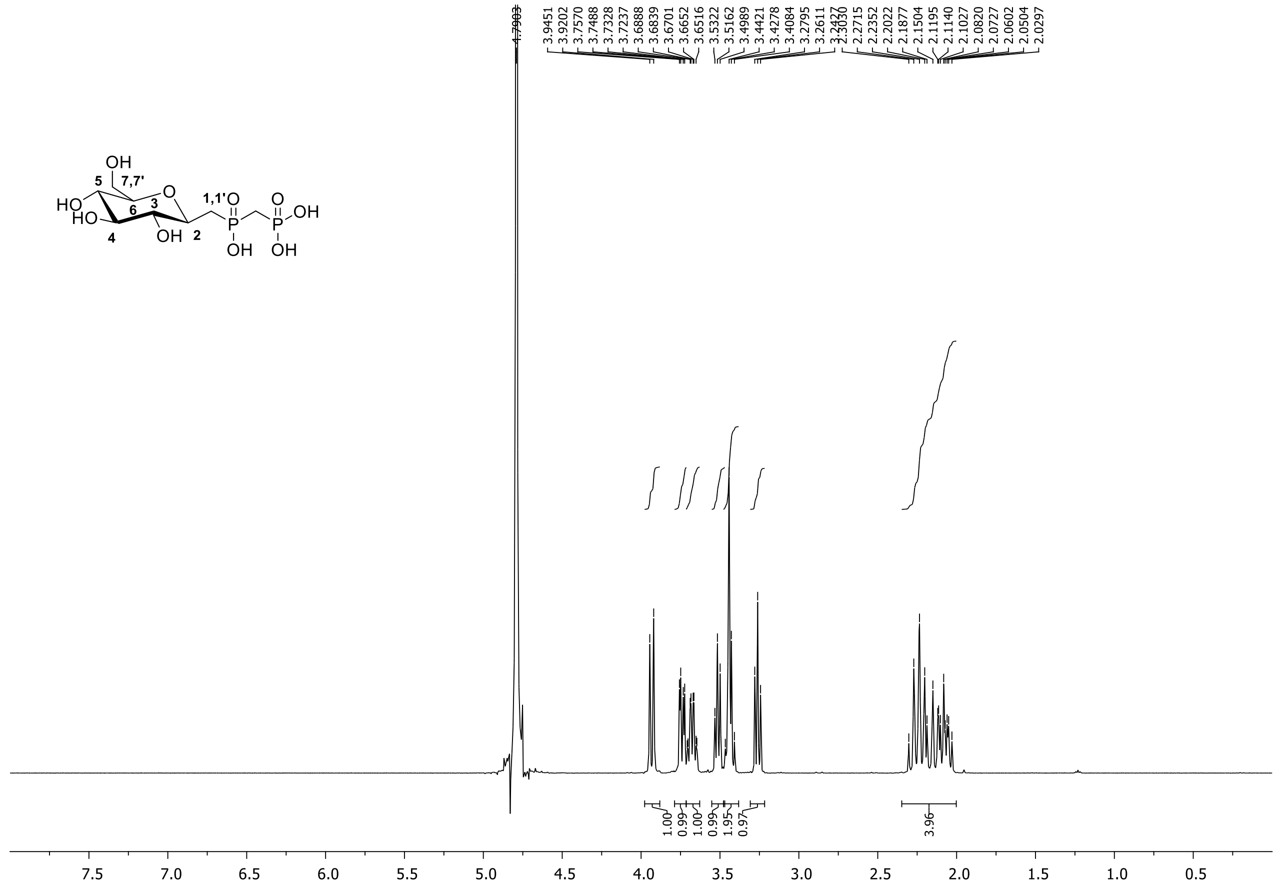

^{13}C NMR, 125 MHz (D_2O), compound **28**

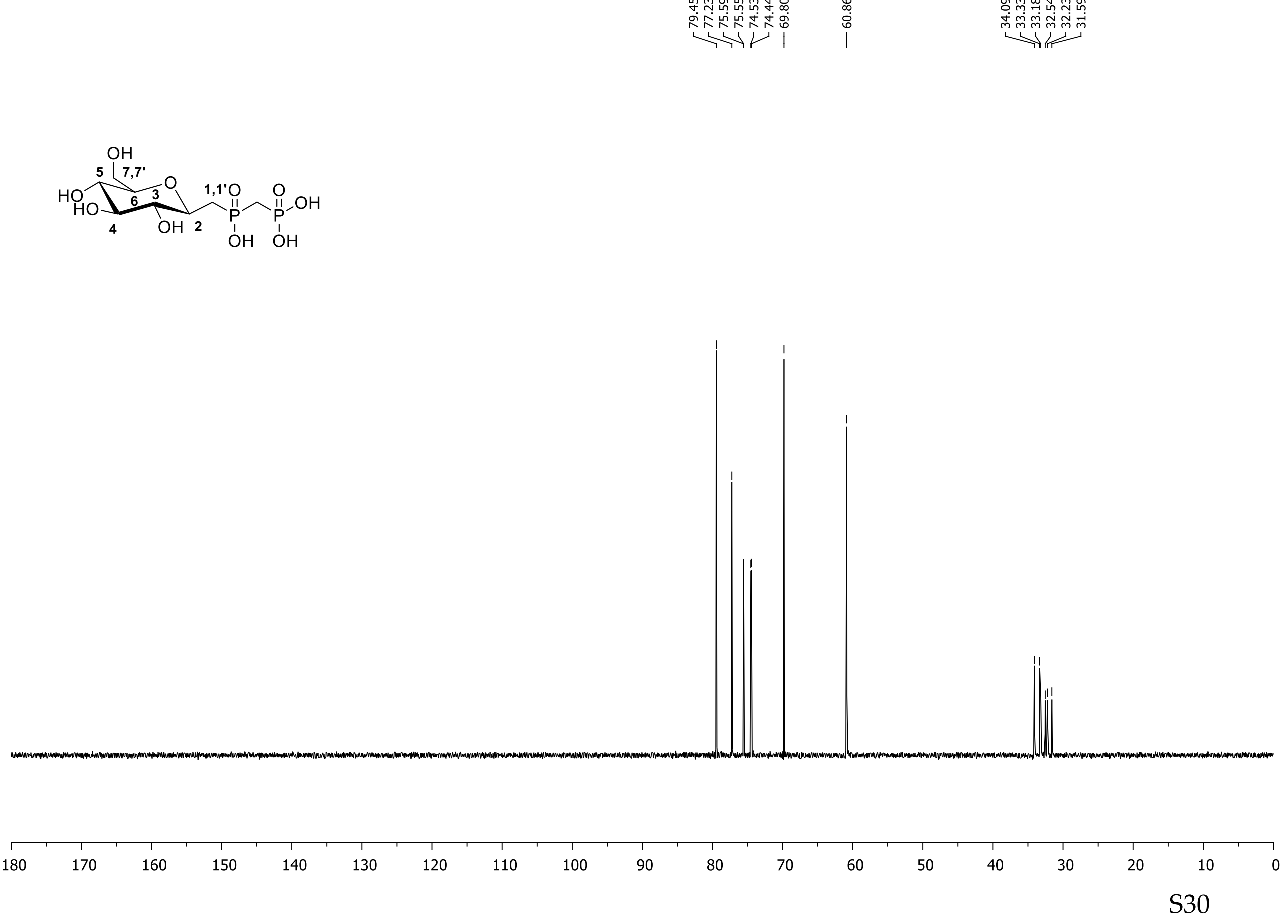

^{31}P NMR, 202 MHz (D$_2$O), compound **28**

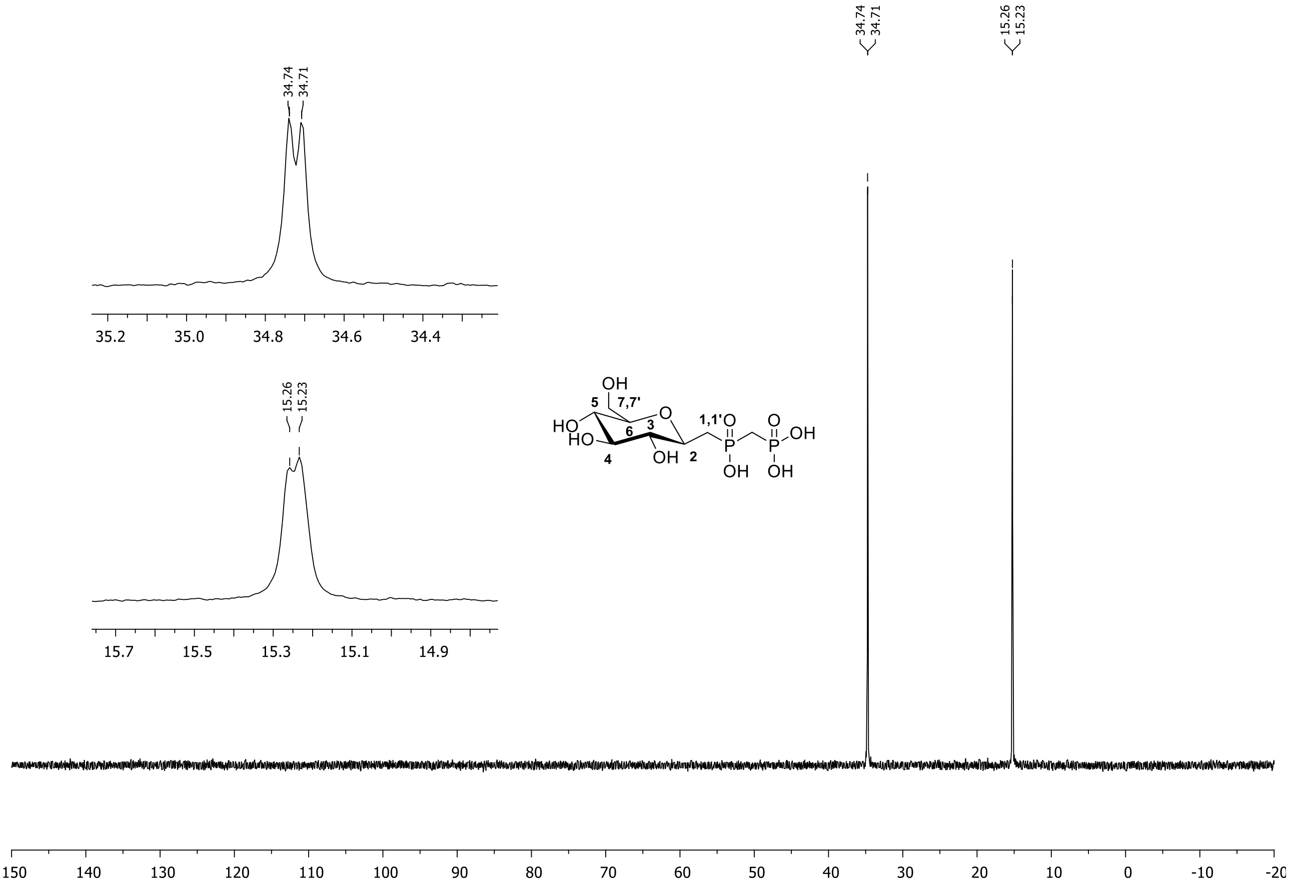

^{31}P NMR non decoupled, 202 MHz (D$_2$O), compound **28**

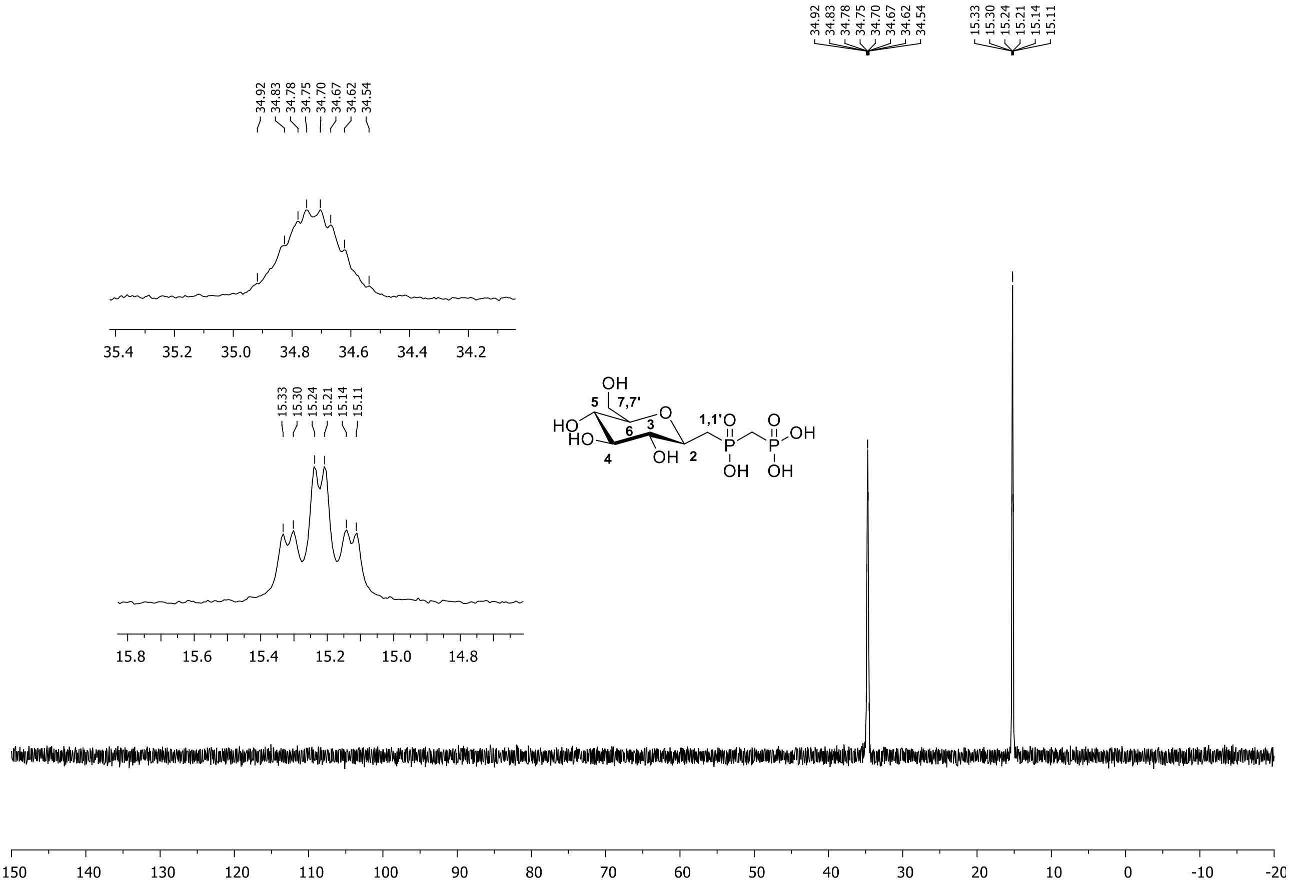

^1H NMR, 500 MHz (D_2O), compound **29**

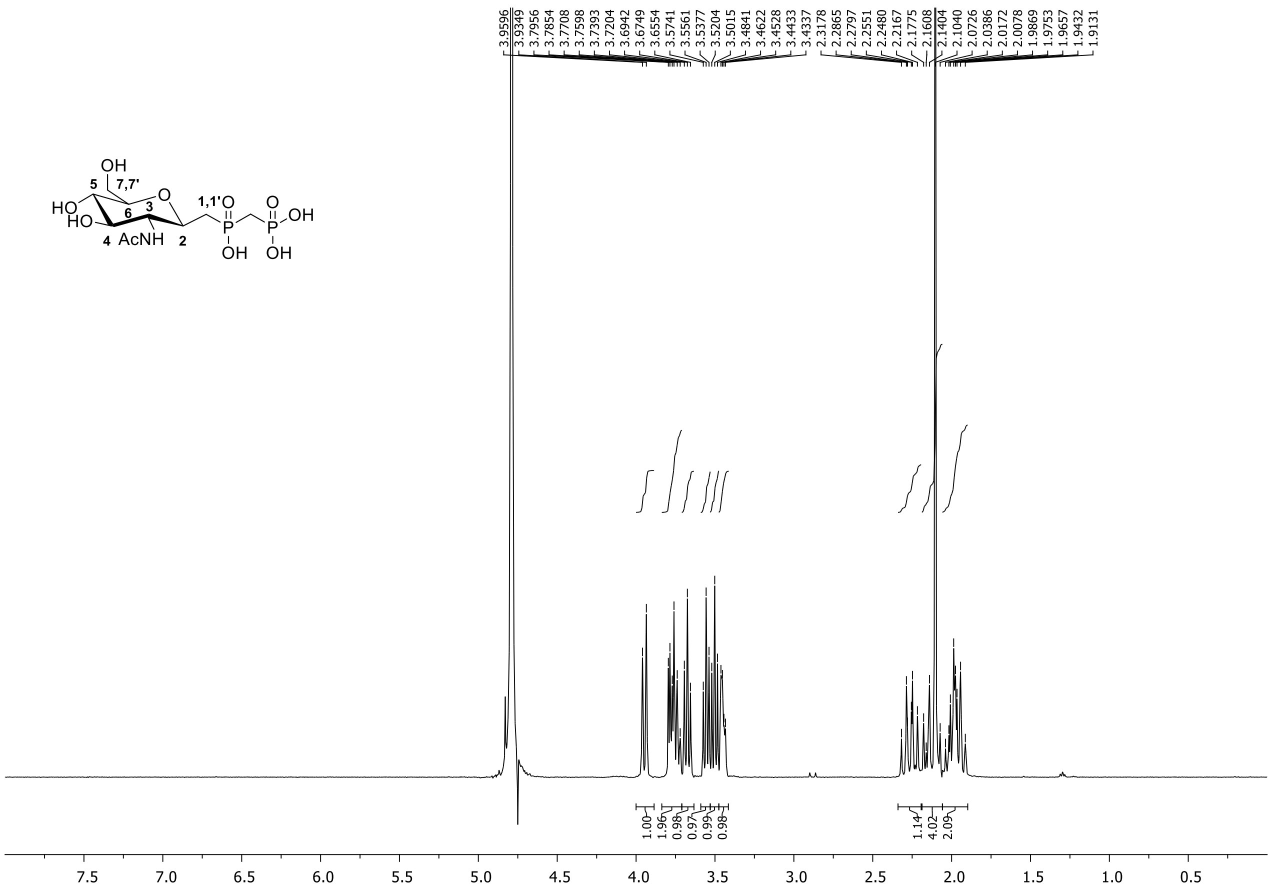

^{13}C NMR, 125 MHz (D_2O), compound **29**

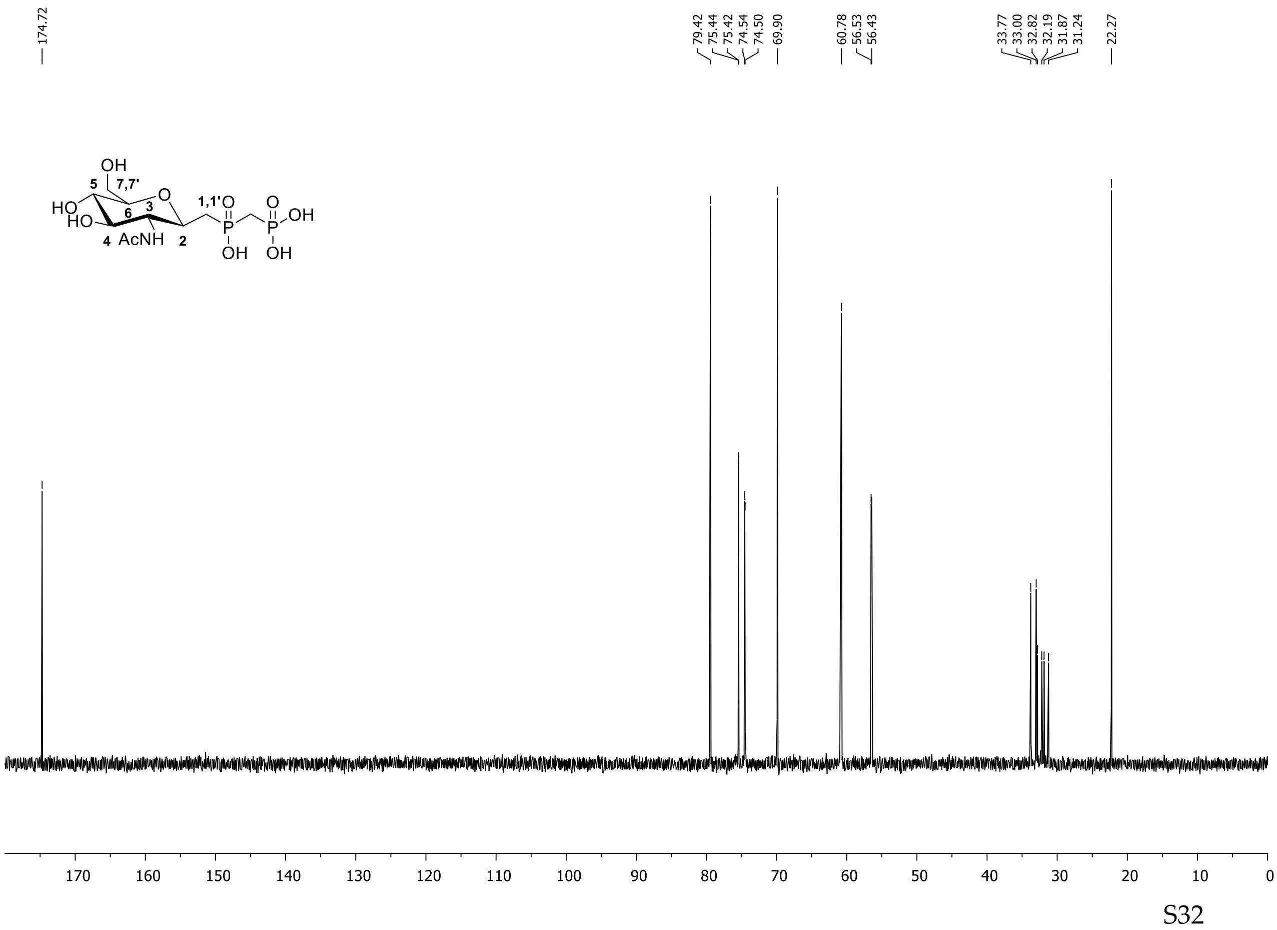

^{31}P NMR, 202 MHz (D_2O), compound **29**

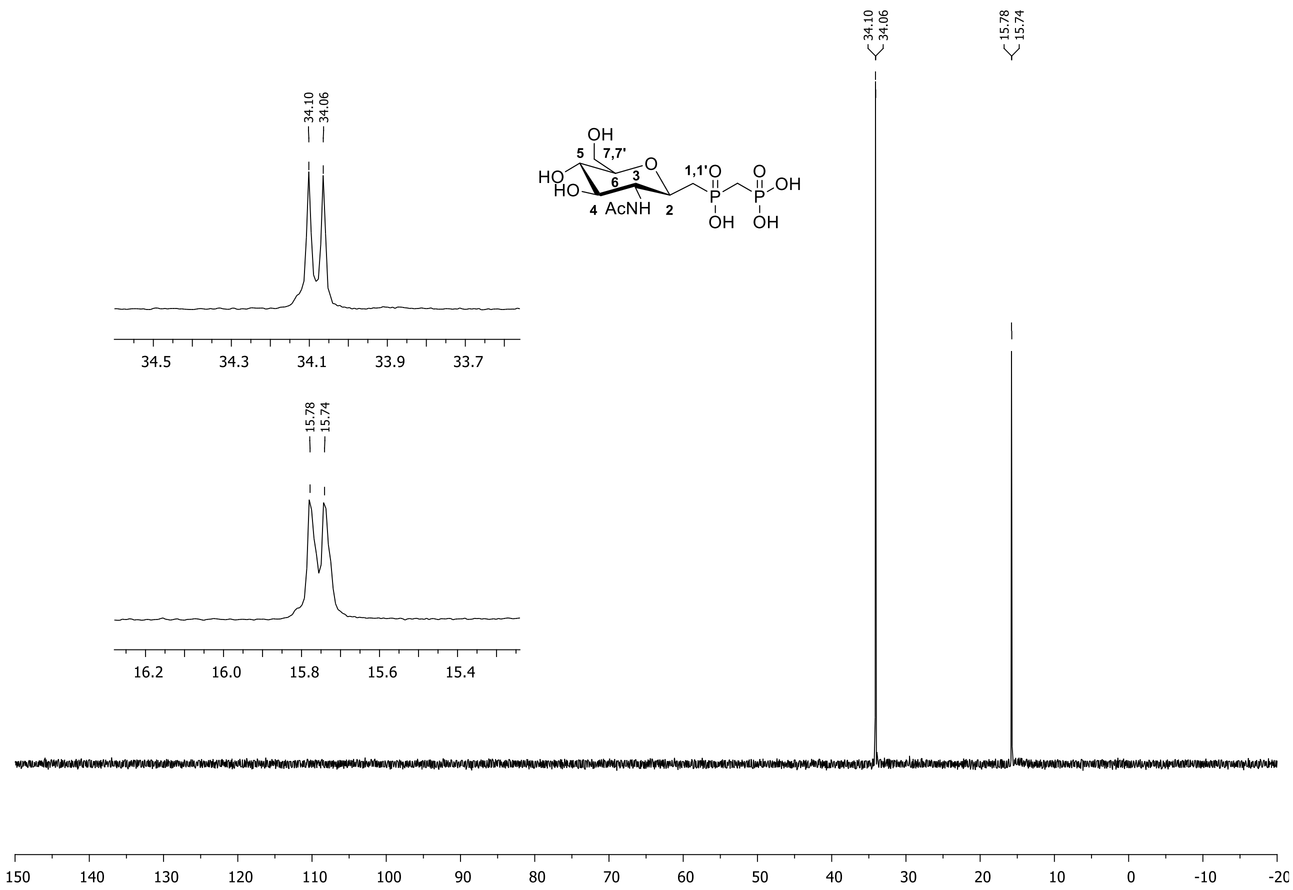

^{31}P NMR non decoupled, 202 MHz (D_2O), compound **29**

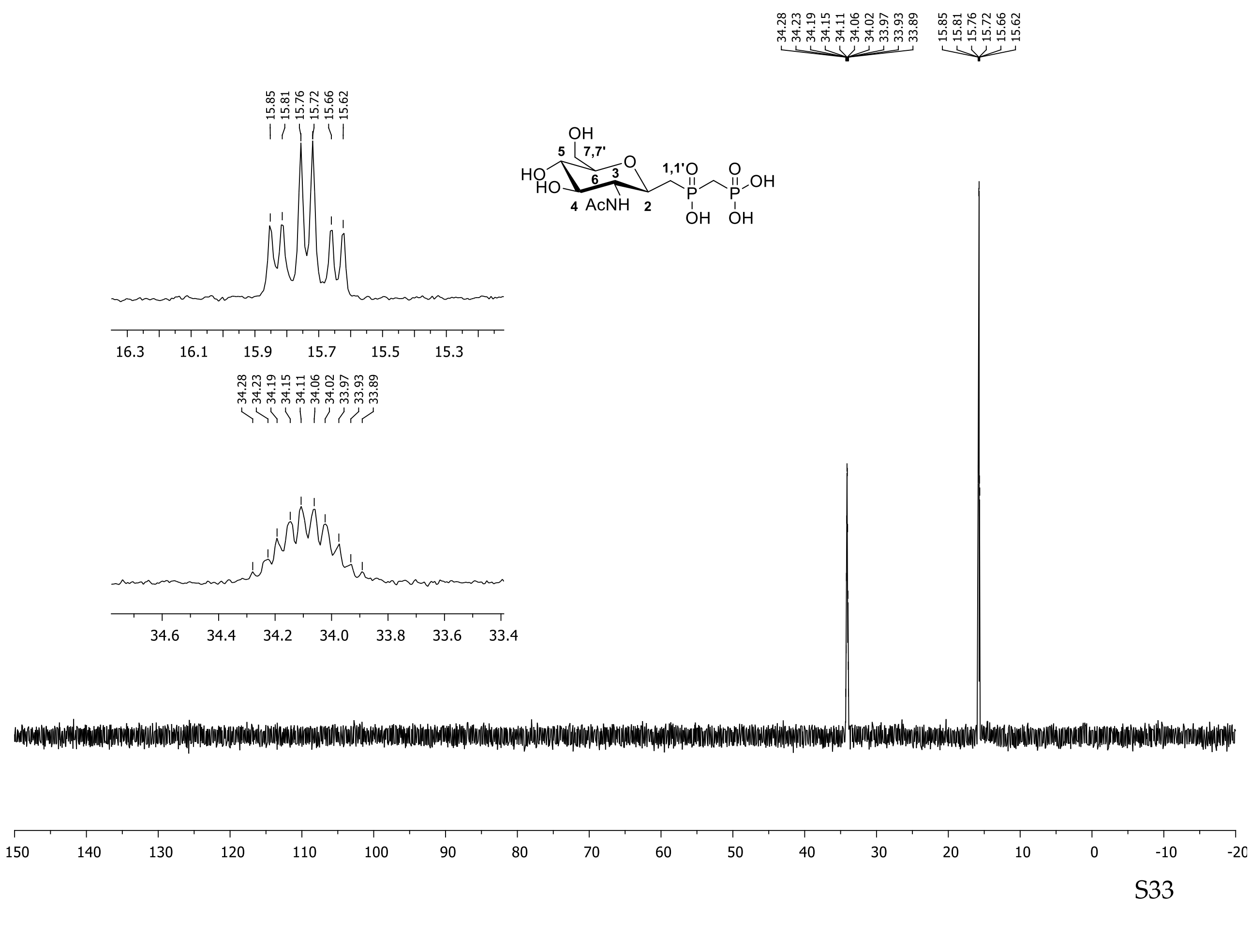

Supplement: Supplementary file 1 [file molecules-25-04969-s001.pdf]
